# Investigating the antimicrobial activity of 1-heteroaryl benzotriazole silver compounds†

**DOI:** 10.1039/d5ra01072a

**Published:** 2025-04-11

**Authors:** Ahmed Elzein, Ghadah Abdullah S. Al Jomeh, Graham J. Tizzard, Simon J. Coles, Christina N. Banti, Sotiris K. Hadjikakou, George E. Kostakis

**Affiliations:** a Department of Chemistry, School of Life Sciences, University of Sussex Brighton BN1 9QJ UK G.Kostakis@sussex.ac.uk; b UK National Crystallography Service, Chemistry, University of Southampton UK; c Biological Inorganic Chemistry Laboratory, Department of Chemistry, University of Ioannina 45110 Ioannina Greece cbanti@uoi.gr shadjika@uoi.gr; d University Research Centre of Ioannina (URCI), Institute of Materials Science and Computing Ioannina Greece

## Abstract

Five (1–5) Ag(i) compounds, derived from different *N*,*N*′-bidentate 1-heteroaryl benzotriazole ligands, were synthesised and characterised using SXRD, IR, UV-Vis, elemental analysis, ESI-MS, and NMR. Variations in ligands and counter-anions produced distinct structures and morphologies. Preliminary antimicrobial testing at the micromolar level, in DMSO, revealed that only compound 2, based on 1-(2-pyridyl)benzotriazole and triflate anion, exhibited interesting antimicrobial properties, thus marking the introduction of a new class of Ag(i) compounds in medicinal inorganic chemistry.

## Abstract 2

We report a family of Ag(i) compounds built from 1-heteroaryl benzotriazole ligands and their antimicrobial behaviour.

## Graphical abstract

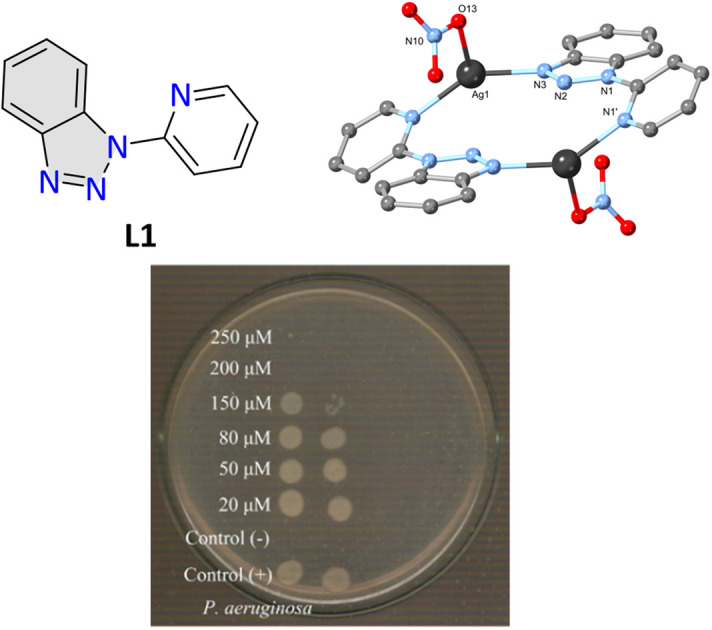

## Introduction

Metal ions are essential in biological processes and human health, with deficiencies causing various diseases. Medicinal inorganic chemistry, with a long history and significant advancements, focuses on integrating metals into medicinal compounds.^1,2^ For example, metal ions are used in diagnostics and therapeutics, such as gadolinium (Gd) in MRI and platinum (Pt) in cancer treatment.^3^ Despite their potential, metal-based agents are underexplored in clinical settings but show higher efficacy against specific pathogens than organic molecules.^3–5^ Metal–N-heterocyclic carbene (NHC) complexes, initially developed for catalytic purposes, are now valuable in drug design due to their stability and modifiability.^6–13^ These complexes, involving metals like gold (Au) and platinum (Pt), demonstrate enhanced biological activity and antitumor properties. The effectiveness of metallodrugs depends on both the metal and its ligands, with biotransformation influencing their form.^4^ Challenges such as side effects, toxicity, and drug resistance underscore the need for new compounds and currently, the investigations for new potent therapeutic agents are focusing on elements like copper (Cu) and silver (Ag).^14,15^

**Scheme 1:**
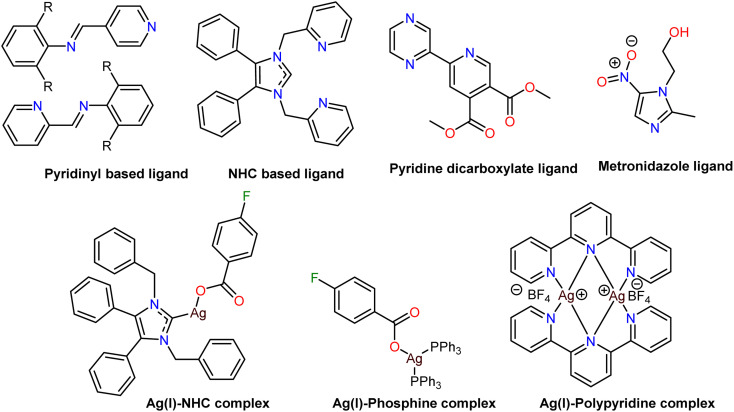
Selected examples of known ligands used in Ag chemistry and silver complexes.

Ag(i) coordination compounds have been recognised as promising therapeutics exhibiting notable biological activity,^16^ especially as antimicrobial agents. However, they display different mechanisms of action, as discussed in a recent review.^17^ These compounds are primarily built by N-heterocyclic carbenes (NHCs), phosphine-based ligands, pyridinyl Schiff base, imidazole or N-heterocycles, polypyridines, imidazole, pyridyl carbenes, pyridine carboxylate (Scheme 1).^18–20^

Ag complexes derived from coumarin and carboxylate entities show antitumor potency, while complexes built using phosphine ligands can inhibit cisplatin-resistant cell lines.^21–23^ Many silver complexes release Ag ions under specific conditions, which can penetrate and disrupt cellular components, leading to membrane damage and oxidative stress. However, current drugs, like Ag sulfadiazine, lose effectiveness quickly due to rapid Ag ion release, necessitating the development of complexes with strongly coordinating ligands to slow this process.^24–28^ Preliminary studies suggest that silver (Ag) complexes may be effective against cisplatin-resistant cancer cell lines, with the potential to overcome some resistance mechanisms.^29–31^ While more studies are needed for Ag complexes to achieve the clinical success of platinum drugs, they offer the advantage of less toxic, more economical alternatives to platinum-based chemotherapy drugs, targeting cancer cell mitochondria and activating ‘cell suicide’ processes.^32,33^ Challenges for Ag complexes include ensuring stable ion release and fully understanding their mechanisms of action; for example, silver based assemblies are especially prone to fragmentation as several studies have established this.^34–36^ However, further research into their pharmacokinetics, toxicity, and efficacy is essential to realise their potential as complementary or alternative therapies.^6–11^

**Scheme 2:**
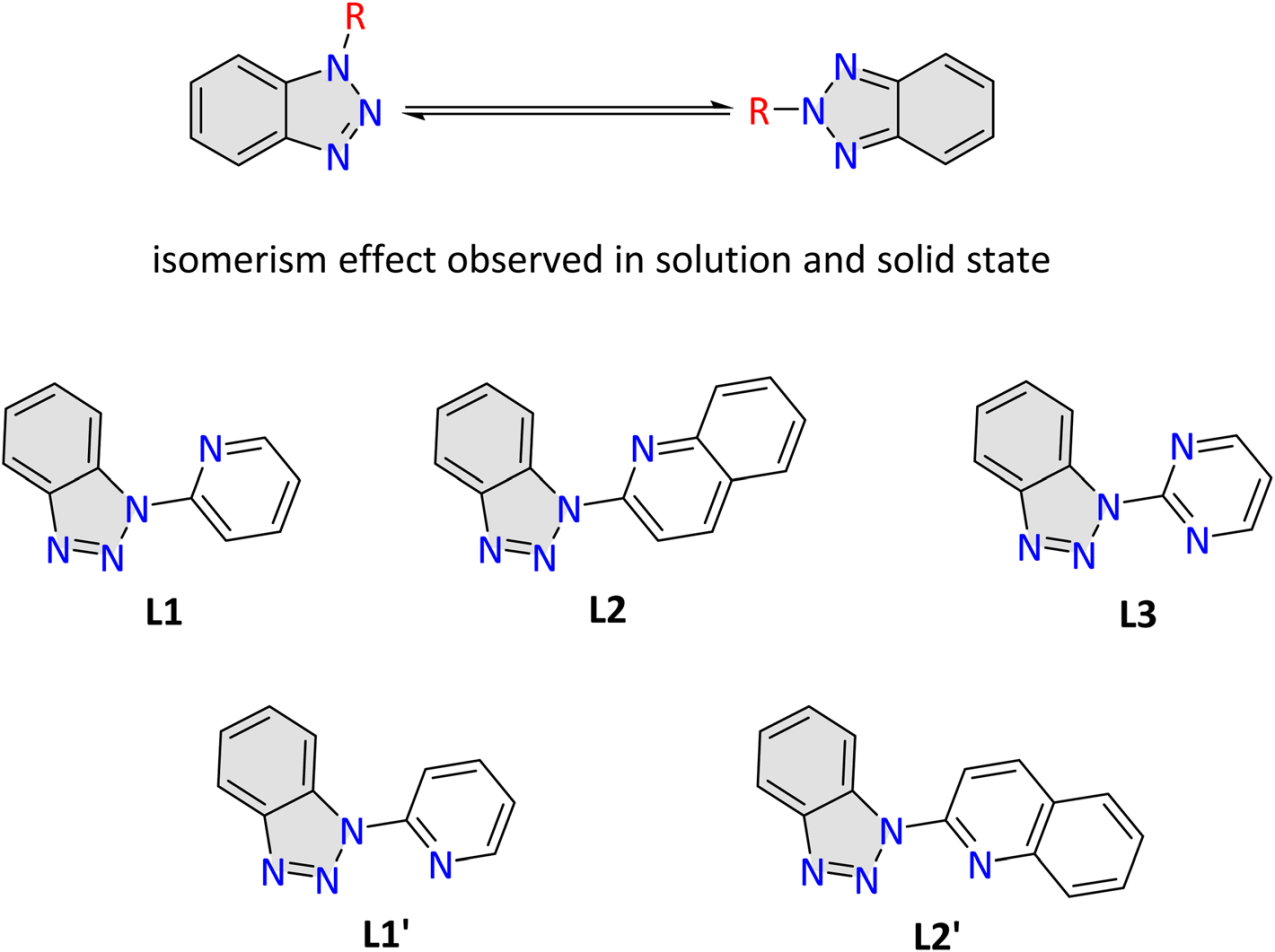
Tautomeric forms of benzotriazole (top). Benzotriazoles derivatives synthesised (middle). Different conformations of L1 and L2 (lower).

Benzotriazole (Scheme 2), an aromatic heterocyclic compound, is a versatile synthetic auxiliary with unique physicochemical properties. It is easily accessible, inexpensive, and widely used in organic and inorganic chemistry.^37^ Benzotriazole and its derivatives exhibit a broad spectrum of biological activities, including antifungal, antibacterial, antiviral, and anticancer effects.^38–41^ Halogenated synthons have often been replaced by benzotriazole surrogates, offering increased stability and reduced toxicity. In oncology, benzotriazole derivatives are effective as protein tyrosine kinase (PTK) inhibitors, competing with ATP for the adenine-binding region of PTKs, thereby inhibiting cancer cell growth by blocking phosphorylation signal transduction and abnormal PTK expression. Additionally, benzotriazoles are useful in medicinal applications such as corrosion inhibitors and UV stabilisers, showcasing their versatility beyond pharmacology.^39,42–46^

Inspired by the remarkable antimicrobial and antitumor properties of silver compounds and the diverse pharmacological effects and potent inhibition of protein tyrosine kinases exhibited by benzotriazoles, we hypothesised that the synergy of these two entities would offer the possibility of developing innovative and highly effective treatments for various challenging diseases; thus, we were driven to explore the therapeutic potential of Ag–benzotriazole-based complexes. Recent work identified that the complexation reactions of 1-substituted benzotriazole ligands with silver salts promote an isomerism effect (Scheme 2, upper).^37^ This phenomenon has been explicitly studied and observed in solutions when R is H or an alkyl group.^47^ The proportions of the two isomers are sensitive to changes in solvent polarity but independent of temperature. Therefore, we decided to limit our investigation to rigid frames (R = aryl group), including heteroatoms that promote chelation or bridging effects and chose the three frames L1, L2 and L3 (Scheme 2, middle). These three frames provide a topologically identical coordination environment; thus, the formation of equivalent topological systems is anticipated.^48^ Ligand L1 and studies on its coordination chemistry have been initially reported by Steel,^49^ whereas recently, several groups, including some of us,^50–52^ have used this ligand for synthesising complexes with applications in catalysis,^53–57^ or fluorescence.^58^ Ligand L2 was also initially reported by Steel, and others, possessing a quinoline moiety; thus, possible monitoring with fluorometers within cells is applicable.^55^ However, from these studies, it became apparent that both ligands may adopt different conformations (Scheme 2, lower) L1′ and L2′, which is possible *via* rotation of the aryl group.^50,55^ To ensure or discard the impact of the chelation effect in the observed biological activity, we incorporated ligand L3; which was previously introduced by Ferraro and Bortoluzzi^58^ Also, we chose two Ag salts: nitrate, a strongly binding anion that promotes chelation effects, and triflate, a weakly bridging binding anion.^59–61^ Thus, we produced a family of five compounds formulated as [Ag_2_(L1)_2_(NO_3_)_2_] (1), [Ag_2_(L1)_2_(OTf)_2_] (2), [Ag(L2)_2_(NO_3_)] (3), [Ag_2_(L2)_2_(OTf)_2_] (4) and [Ag_2_(L3)_2_(OTf)_2_] (5). Compound 1 has already been reported by Steel in the parent paper.^49^ To the best of our knowledge, this study represents the first in-depth biological investigation of this class of Ag compounds, highlighting their potential therapeutic applications.

## Results

### Ligand synthesis

Following already known protocols, the 1-heteroaryl substituted benzotriazole ligands can be synthesised using commercially available chemicals in one high-yielding step, avoiding column chromatography.^56^ The reaction incorporates a nucleophilic aromatic substitution of the corresponding bromo-analogue. The ligands were characterised by NMR (^1^H, ^13^C), IR, UV-Vis, and HR-MS (with ESI as ion source) see ESI.† The reaction yield for the ligands is 99% (L1), 83%(L2) and 99% (L3). Interestingly, single X-ray diffraction studies confirmed the structure of L2 and excluded the presence of the second conformer L2′. We recorded the ^1^H NMR data in different deuterated solvents to identify tautomers. The spectrum profile in d_6_-DMSO is different compared to CDCl_3_ however, no tautomer could be detected (Fig. S1†).

### Complexes synthesis

**Scheme 3:**
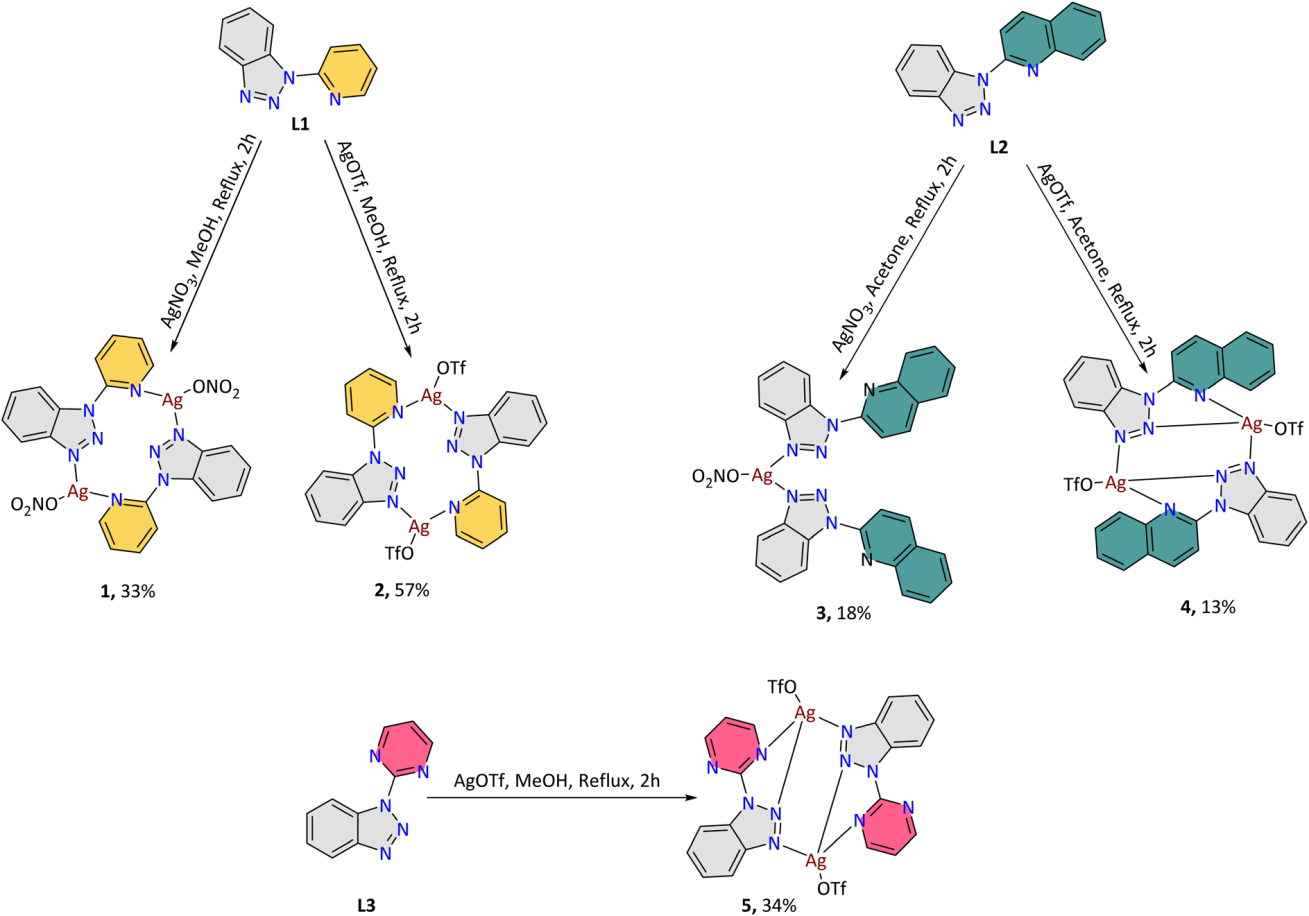
The synthetic procedures followed for the synthesis of the Ag family reported in this work. The yields refer to the isolated crystalline material.

With the L1–L3 ligands in hand and bulk, we performed complexation reactions to synthesise the corresponding Ag compounds. We screened parameters such as concentration, time (2 to 24 h), solvent (MeOH, acetone, CH_3_CN), metal : ligand ratio (3 : 1 to 1 : 3) and temperature (25 °C, 50 °C, 75 °C) to identify the optimum conditions as: AgX : ligand, where X = NO_3_ or OTf in a molar ratio 1 : 1 in MeOH (15 ml). The metal salt and ligand were dissolved in methanol, and after 1 h of reflux, the solution was filtered, and the filtrate was kept for slow evaporation. Shiny, colourless crystals were collected in low to good yields between four and fourteen days (Scheme 3) and were subsequently characterised by single-crystal X-ray diffraction. All samples underwent elemental analysis, confirming accuracy and closely matching the expected values. For all complexation reactions, a significant amount of colourless white precipitate was collected during filtration. Given the low yield of the final product, we conducted ^1^H-NMR and elemental analysis to determine the speciation of the precipitate. To our delight, the results showed the absence of other species and closely matched the crystalline form (Fig. S2† and table for elemental analysis); therefore, by combining the crystals and the precipitates, we achieved a total yield of approximately 60% for all compounds (based on the metal salt), which is considered satisfactory.

### Complex characterisation in solid state

**Fig. 1:**
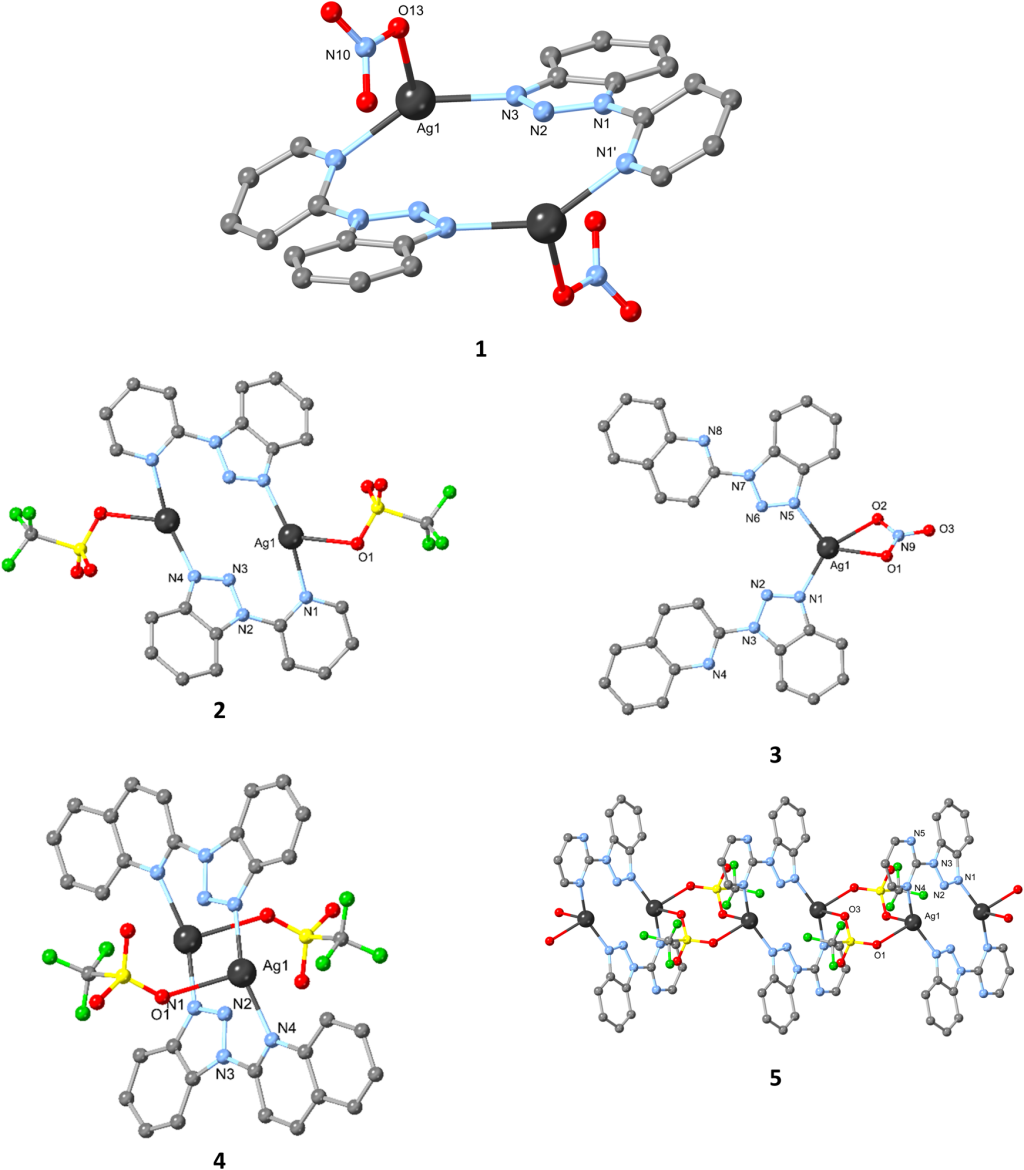
Crystallographic representation of the Ag compounds reported in this work. Colour code; Ag (dark grey), O (red), S (yellow), N (blue), C (grey), F (green).

Compound 1 was synthesised using silver nitrate and the L1 ligand. The structure, already reported in the literature,^49^ crystallises in the triclinic *P*1̄ space group, and its asymmetric unit consists of one Ag(i) centre, one L1 molecule, and one nitrate anion. Each ligand L1 coordinates to two Ag(i) centres (Fig. 1 and Scheme S1†). Notably, the angle of the planes the pyridine and benzotriazole entities form is set at 33.799°, signifying moderate deviation from planarity. The nitrate anion acts as a terminal ligand. As a result, compound 1 can be described as a dimer (Fig. 1) with the Ag⋯Ag distance set at 4.5917(52) Å. Each metal centre possesses a T-shaped geometry and a (N_2_O) coordination environment. The Ag–N [2.240(2)Å N^benzo^ and 2.273(2) Å N^py^] and Ag–O [2.590(2) Å] bond distances are in the normal range.

Compound 2 was synthesised using silver triflate and the L1 ligand. The compound crystallises in the triclinic *P*1̄ space group, and its asymmetric unit consists of one Ag(i) centre, one L1 molecule, and one triflate anion. The triflate anion acts as a terminal ligand, and each ligand L1 coordinates to two Ag(i) centres, similarly observed to 1, thus forming dimeric entities (Fig. 1) with the Ag⋯Ag distance being set at 4.3773(25) Å. Notably, the angle of the planes the pyridine and benzotriazole entities form is set at 36.097°, implying that the use of the triflate anion further enhances deviation from planarity. Each metal centre possesses a T-shaped geometry and a (N_2_O) coordination environment. The Ag–N [2.2047(9)Å N^benzo^ and 2.2839(10)Å N^py^] and Ag–O [2.5989(10) Å] bond distances are in the normal range. However, by expanding the permittable Ag–O bond,^62–64^ then a second Ag–O bond can be identified [2.6469(20) Å], thus the triflate anion acting as a bridge that extends the dimers into one dimension, however this notion will not be further taken into account.

Compound 3 was synthesised using silver nitrate and the L2 ligand. The compound crystallises in a polar monoclinic *Ia* space group, and its asymmetric unit consists of one Ag(i) centre, surprisingly two L2 molecules, and one nitrate anion. A possible explanation for this surprising phenomenon is a crystallization effect, potentially influenced by aromatic interactions between adjacent entities. Each ligand L2 coordinates to one Ag(i) centre (Fig. 1). The ligand's configuration in 3 recalls the crystallographically characterised L2, in which the nitrogen N atom of the quinoline moiety points towards the phenyl group of the benzotriazole entity. The angle of the planes between the quinoline and benzotriazole entities is set at 2.031 and 4.427°, indicating a minimal deviation from planarity. The nitrate anion acts as a terminal chelating ligand. As a result, compound 3 can be described as a monomer (Fig. 1). Each Ag centre possesses a distorted T-shaped geometry and a (N_2_O_2_) coordination environment. The Ag–N [2.2580(99) Å and 2.3107(100) Å N^benzo^] and Ag–O [2.4395(91) and 2.4327(97) Å] bond distances are in the normal range.

Compound 4 was synthesised using silver triflate and the L2 ligand. The compound crystallises in triclinic *P*1̄ space group, and its asymmetric unit consists of one Ag(i) centre, one L2 molecule, and one triflate anion. The triflate anion acts as a terminal ligand, whereas ligand L2 coordinates to two Ag(i) centres. In this case, the nitrogen N atom of the quinoline moiety points towards the triazole phenyl group of the benzotriazole entity and creating a six membered chelated ring. The angle of the planes between the quinoline and benzotriazole entities is set at 19.108°, indicating significant deviation from planarity, compared to 3. As a result, compound 4 can be described as a dimer (Fig. 1) with the Ag⋯Ag distance set at 4.2965(211) Å. Each Ag centre possesses a distorted T-shaped geometry and a (N_2_O) coordination environment. The Ag–N [2.2656(100) Å N^quino^ and 2.2225(112) Å] and Ag–O [2.5522(114) Å] bond distances are in the normal range.

Compound 5 was synthesised using silver triflate and the L3 ligand. The compound crystallises in the monoclinic *P*2_1_/*c* space group, and its asymmetric unit consists of one Ag(i) centre, one L3 molecule, and one triflate anion. Each ligand L3 coordinates to a total of two Ag(i) centres, thus forming dimeric entities (Fig. 1) with the Ag⋯Ag distance set at 4.4215(5) Å. The angle of the planes the pyrimidine and benzotriazole entities form is set at 13.902°, signifying minimal deviation from planarity. The triflate anion acts as a bridging ligand. As a result, compound 5 can be described as a dimeric entity that extends to one dimension *via* the triflate anion. Each metal centre possesses a distorted tetrahedral geometry and a (N_2_O_2_) coordination environment. The Ag–N [2.2281(29)Å N^benzo^ and 2.2948(36) Å N^pyri^] and Ag–O [2.5588(26) and 2.5777(30) Å] bond distances are in the normal range. By comparing structures 2 and 5, the major difference lies in the deviation of the benzotriazole and pyridine (36.097°) or pyrimidine (13.902°) rings. This structural variation may be responsible for enabling the triflate group to act as a bridging entity, linking the AgL_3_ units to form one-dimensional chains (Table 1).

**Table 1:** Summarising table for important structural features observed in compounds 1–5

Entry	Compound	Morphology	Dimensionality (*D*)	Coordination number/geometry	Ag⋯Ag distance (Å)	Ligand (deviation from planarity of aryl and benzotriazole entities)
1	1	Dimer	0	N_2_O/3/T-shaped	4.5917(52)	33.799°
2	2	Dimer	0	N_2_O/3/T-shaped	4.3773(25)	36.097°
3	3	Monomer	0	N_2_O_2_/4/T-shaped	None	2.031° & 4.427°
4	4	Dimer	0	N_2_O/3/T-shaped	4.2965(211)	19.108°
5	5	Dimer	1	N_2_O_2_/4/tetrahedral	4.4215(5)	13.902°

### Complex characterisation in solution

To explore the correlation between the solid-state structure and solution behaviour of compounds 1–5, we first investigated whether the selected ligands are subject to tautomerism, a phenomenon influenced by the solvent. Hence, ^1^H NMR data were recorded for the ligands in various deuterated solvents (Fig. S1†). Although the spectral profile in d_6_-DMSO differed significantly from that in CDCl_3_, no tautomers were detected (Fig. S1†). Notably, the same ^1^H NMR profile was observed in data recorded at elevated temperatures (70 °C) and after storing the NMR tubes at room temperature for ten days, indicating that tautomerism is not favoured in either solvent. With the complexes in hand, we attempted to elucidate if they retain the morphology observed with single X-ray diffraction in solution. We recorded ^1^H NMR, UV-Vis and ESI-MS spectra, and conducted diffusion NMR studies at a specific concentration 0.012 M. Most silver-based drugs, such as silver sulfadiazine, function by releasing Ag(i) ions that infiltrate cell membranes and disrupt their normal function. However, their efficacy diminishes rapidly due to the swift release of these ions. Despite the samples (1–5) being kept from light, air, and excessive heat, the ^1^H NMR data proved ambiguous, as only minor shifts were observed for compounds 3 and 4 (ESI Fig S2†). These data suggest the presence of a “free” ligand and possibly the presence of other species in the solution. ESI-MS data for compounds 1–5 validate the formation of monomeric and dimeric species; a characteristic peak with corresponding isotropic distribution can be identified for both species (Table S2 and corresponding figures in ESI† for each complex). Then, we recorded diffusion ^1^H NMR data using a coordinating solvent (d_6_-DMSO) and a specific concentration (0.012 M) and compared them with the free ligands (corresponding figures in ESI† for each complex). The data suggest the existence of several distinct species, along with various fragmentation patterns, which could reflect the complexity of the sample and the diverse chemical interactions taking place. While the data did not provide definitive confirmation of a singular dominant species, it points to a dynamic system with multiple molecular components and fragmentation pathways. Lastly, we attempted to record the CV of compound 1 (Fig S4†) in the same solvent that the NMR and biological studies (see below) were carried out. The initial effort to record the cyclic voltammetry of compound 1 at 0.012 M in DMSO with 0.1 M NH_4_ClO_4_ provided inconclusive data, and an attempt to substitute the supporting electrolyte with NH_4_PF_6_ supporting electrolyte afforded colloidal Ag, therefore we did not attempt to record the CV data for the remaining compounds.

### Bacterial studies. Antibacterial effect of compounds on the growth of microbial strains

The stability of the compounds was evaluated over a 48 hour period in DMSO solution using UV-Vis spectroscopy (5 × 10^−5^ M). The UV-Vis data for all five compounds show notable variations in the absorbance profiles, particularly at the maximum peak around ∼300 nm—1 (306 nm), 2 (306 nm), and 5 (300 nm), as well as at 315 nm and 331 nm for compounds 3 and 4, compared to the free ligand both at time zero and throughout the 48 hour period. When comparing the UV-Vis spectra of the free ligands and their respective complexes, the absorbance at 300 or 315/331 nm nearly doubles at time zero, with a slight decrease in intensity observed over time, suggesting reasonable stability under these conditions. This difference suggests interactions between the ligands and silver entities, indicating the presence of one (or more) complex(es) with an unknown identity(ies) and formula(e). Such changes may point to dynamic equilibrium processes or adjustments occurring within the system as time progresses. The chosen 48 hours period corresponds to the maximum incubation time for bacterial strains. (Fig. S5†). The antimicrobial activity of compounds 1–5 and their respective ligands (L1–L3) was evaluated against Gram-negative strains *Pseudomonas aeruginosa* and *Escherichia coli*, as well as Gram-positive strains *Staphylococcus epidermidis* and *Staphylococcus aureus*. The growth of microbial strains was assessed based on the Minimum Inhibitory Concentration (MIC), Minimum Bactericidal Concentration (MBC), and Agar Diffusion Method (Inhibition Zone, IZ). MIC is the lowest concentration required to inhibit bacterial growth, while MBC is the concentration needed to eliminate 99.9% of the bacterial inoculum. The Inhibition Zone (IZ) is the diameter (in mm) of the area where bacterial growth is inhibited by the compound.^65,66^ The results were compared to those for AgNO_3_ and AgOTf.^67^ MIC values for the Ag(i) compounds ranged from 55.9 to 250 μM against Gram-negative bacteria and 75.9 to 250 μM against Gram-positive bacteria (entries 1–10, Table 2). At a concentration of 250 μM, the ligands exhibited no activity against any of the bacterial strains (entries 6–8, Table 2).

The coordination compounds built with L1 ligand (1 and 2) demonstrate the highest antimicrobial activity among all tested compounds. The MIC values for 1 and 2 range from 60.5 to 130.8 μM, while those for compound 2 range from 55.9 to 118.8 μM against Gram-negative and Gram-positive bacteria (entries 1 and 2, Table 2). For ligand L2, the Ag(i) compounds 3 and 4 demonstrate lower activity than those involving ligand L1. Compounds 3 and 4 demonstrate activity solely against the bacterial strain *P. aeruginosa*, with MIC values of 215.3 and 166.4 μM, respectively (entries 3 and 4, Table 2). However, no activity is observed for the remaining bacterial strains up to the tested concentration of 250 μM (entries 3 and 4, Table 2). For 5, the MIC values range from 89.8 to 230.9 μM. Ag(i) compounds built with L1 ligand exhibit greater activity than those with L2 and L3. Additionally, the counter anion, whether NO_3_ or OTf, appears to have no impact on the observed reactivity.

Compound 1 demonstrates comparable activity against Gram-negative bacteria with that of AgNO_3_, but for Gram-positive bacteria, compound 1 exhibits 1.6- and 1.3-fold greater activity than that of AgNO_3_. Notably, for 2, its activity is 1.1-, 1.7-, 1.7-, and 1.4-fold stronger than AgNO_3_ against *P. aeruginosa*, *E. coli*, *S. epidermidis*, and *S. aureus*. The remaining compounds display lower activity compared to AgNO_3_ or AgOTf.

### Minimum bactericidal concentration testing

The range of MBC values for the compounds falls between 150 and greater than 250 μM (entries 11–20, Table 2). The lowest MBC value of 150 μM is observed compounds 1 and 2 against *S. epidermidis* (entries 11 and 12, Table 2).

**Table 2:** Minimum inhibitory concentration (MIC), bactericidal concentration (MBC) and inhibition zone (IZ) of compounds 1–5 and ligands L1, L2 and L3 against bacterial strains *P. aeruginosa*, *E. coli*, *S. epidermidis* and *S. aureus*

Entries	Compound	*P. aeruginosa*	*E. coli*	*S. epidermidis*	*S. aureus*	Ref.
**MIC (μM)**						
1	1	60.5 ± 7.6	130.8 ± 11.5	77.5 ± 4.2	75.9 ± 2.2	a
2	2	55.9 ± 8.1	80.5 ± 8.9	100.4 ± 9.6	118.8 ± 14.7	a
3	3	215.3 ± 21.7	>250	>250	>250	a
4	4	166.4 ± 7.7	>250	>250	>250	a
5	5	89.8 ± 6.1	230.9 ± 11.6	110.3 ± 7.8	195.8 ± 11.6	a
6	L1	>250	>250	>250	>250	a
7	L2	>250	>250	>250	>250	a
8	L3	>250	>250	>250	>250	a
9	AgNO_3_	60.0	184.8 ± 10.5	122.0	95.0	66–69
10	AgOTf	63.6 ± 8.9	134.6 ± 12.3	167.0 ± 5.6	166.4 ± 9.8	

**MBC (μM)**						
11	1	>250	>250	150 ± 0.0	162.5 ± 24.5	a
12	2	233 ± 33	>250	150 ± 0.0	162.5 ± 24.5	a
13	3	>250	>250	>250	>250	a
14	4	>250	>250	>250	>250	a
15	5	250.0 ± 0.0	238.0 ± 25.0	225.0 ± 28.0	250.0 ± 0.0	a
16	L1	>250	>250	>250	>250	a
17	L2	>250	>250	>250	>250	a
18	L3	>250	>250	>250	>250	a
19	AgNO_3_	153.3 ± 13.1	173.3 ± 18.0	140.0	135.0 ± 6.7	66–69
20	AgOTf	90.0 ± 8.8	>250	225.0 ± 28.3	218.0 ± 18.0	

**MBC/MIC**						
21	1	—	—	1.9	2.1	a
22	2	4.2	—	1.5	1.4	a
23	3	—	—	—	—	a
24	4	—	—	—	—	a
25	5	2.8	1.0	2.0	1.3	a
26	AgNO_3_	2.6	0.9	1.2	1.4	66–69
27	AgOTf	1.4	—	1.3	1.3	

**IZ (mm)**						
28	1	12.3 ± 1.7	9.8 ± 0.3	11.5 ± 0.6	11.3 ± 0.7	a
**29**	**AgNO**_**3**_ **/L1 (1 : 1)**	**12.5 ± 0.9**	**10.0 ± 0.0**	**10.5 ± 0.9**	**10.5 ± 0.9**	
30	2	13.9 ± 1.2	12.3 ± 1.5	11.2 ± 0.4	11.7 ± 0.4	a
**31**	**AgOTf/L1 (1 : 1)**	**12.5 ± 0.9**	**10.0 ± 0.0**	**10.5 ± 0.9**	**10.5 ± 0.9**	
32	3	9.8 ± 0.3	9.3 ± 0.4	9.1 ± 0.2	9.3 ± 0.4	a
**33**	**AgNO**_**3**_ **/L2 (1 : 1)**	**13.5 ± 0.9**	**10.0 ± 0.0**	**10.5 ± 0.9**	**10.5 ± 0.9**	
34	4	11.5 ± 0.9	9.8 ± 0.3	10.0 ± 0.0	10.0 ± 1.1	a
**35**	**AgOTf/L2 (1 : 1)**	**13.8 ± 1.5**	**10.0 ± 0.0**	**10.5 ± 0.9**	**10.5 ± 0.9**	
36	5	12.7 ± 1.3	9.8 ± 0.5	12.3 ± 1.7	10.0 ± 0.0	a
**37**	**AgOTf/L3 (1 : 1)**	**12.0 ± 1.9**	**9.8 ± 0.5**	**10.0 ± 0.0**	**10.5 ± 0.1**	
38	L1	9.0 ± 0.0	9.0 ± 0.0	9.0 ± 0.0	9.0 ± 0.0	a
39	L2	9.0 ± 0.0	9.0 ± 0.0	9.0 ± 0.0	9.0 ± 0.0	a
40	L3	9.0 ± 0.0	9.0 ± 0.0	9.0 ± 0.0	9.0 ± 0.0	a
41	AgNO_3_	13.0	10.1 ± 0.8	14.0	12.0	66–69
42	AgOTf	15.7 ± 0.7	10.5 ± 1.5	11.5 ± 0.6	12.7 ± 0.7	

a This work.

The ratio of MBC to MIC categorises an antibacterial agent as either bacteriostatic or bactericidal. When the MBC/MIC ratio is ≤2, the antibacterial agent qualifies as bactericidal, indicating its capability to eliminate 99.9% of the microorganisms. Conversely, if the MBC/MIC ratio is ≥4, the agent is deemed bacteriostatic, as it solely inhibits but does not eradicate the microorganism.^65–67^ Thus, compounds 1 and 2 demonstrate bactericidal properties against *S. epidermidis* and *S. aureus*, while compound 5 behaves as a bactericidal agent against *E. coli*, *S. epidermidis*, and *S. aureus*.

### Determination of the inhibition zone (IZ) through agar disc-diffusion method

The antimicrobial efficacy of compounds 1–5 and the ligands underwent evaluation through the agar diffusion assay (entries 28–37, Table 2). Paper discs with a diameter of 9 mm were soaked in a solution of the compounds (1 mM). These discs were then placed on agar plates and were incubated at 37 °C for 20 hours. The resulting IZ values for 1–5 against the tested bacterial strains range between 9.8–12.3 mm, 11.2–13.9 mm, 9.1–9.8 mm, 9.8–11.5 mm, and 9.8–12.7 mm, respectively. The Ag(i) compounds generally demonstrate larger inhibition zones than those observed for the ligands and the corresponding Ag(i) salts. This pattern aligns with the trend observed in MIC values, where compounds 1 and 2 perform better than those built with L2. Notably, compound 2 displays the highest activity comparable to that of AgOTf. Shungu *et al.*^65,66^ classified microorganisms into three categories depending on the size of the IZ caused by antibiotics. According to this classification, (i) strains with an IZ of ≥17 mm are deemed susceptible to the antibiotic, (ii) those with an IZ between 13 and 16 mm (13 ≤ IZ ≤ 16 mm) are considered intermediate, and (iii) microorganisms displaying an IZ of ≤12 mm are categorised as resistant strains to the antibiotic. The IZs formed in the cases of compounds 1, 3, 4 and 5 categorise them as resistant (entries 28 and 30–32, Table 2); however, compound 2, is classified as intermediate in the case of *P. aeruginosa*, with an IZ of 13.9 mm (entry 29, Table 2).^67,68^

To verify whether the complexes maintain their integrity during biological measurements, mixtures of their reactant components (AgNO_3_ or AgOTf and ligands L1–L3 at 1 mM) were prepared in the same 1 : 1 ratio used for complex synthesis and utilised to determine the relevant parameters. In experiments with mixtures of the complex's components, the observed biological effect remained consistent, regardless of the type of ligand or silver salt used (Table 2). The IZ values for the complexes differ from those observed when mixtures of their components are used. This indicates that the activity is attributed to the complete complex rather than its components, further supporting the stability of the complexes in solution (Table 2).

## Discussion

This study investigates the antimicrobial activity of 1-heteroaryl benzotriazole silver compounds for the first time. It explores how variations in anions and ligands influence their effectiveness. Although we aimed to establish the stability of these silver complexes and compare their solid-state and solution structures at the millimolar level, the data from ESI-MS, UV-Vis, ^1^H-NMR, and CV suggest the presence of multiple species, reflecting the complexity of their dynamic system. This multiplicity complicates the structure-bioactivity correlation. However, UV-Vis studies at micromolar concentrations provided insights into the electronic interactions between silver and the ligand. A notable difference in the ligand-to-metal charge transfer (LMCT) transition (Fig. S5†) between the free ligand(s) and the corresponding complex(es) verifies the presence of a complex with uncertain speciation. Moreover, this electronic shift supports the hypothesis that the observed antimicrobial activity is likely due to this complex (the reader should compare the data in Table 2), rather than free silver ions. It validates the presence of a complex—potentially the one characterised by single-crystal X-ray diffraction. However, it remains uncertain, and caution is advised, whether this is the major species responsible for the observed difference in bioactivity. The latter, in turn, underscores the significance of ligand coordination in modulating the properties of silver compounds and emphasises the necessity of characterising these species prior to biological evaluation, rather than relying on the *in situ* generation of Ag–ligand species. A hypothesis is that pre-isolated species may minimise the presence of various fragments in solution, potentially enabling more reliable and accurate structure–activity relationship studies. Further investigations using alternative spectroscopic techniques and more controlled solution conditions are needed to clarify the speciation and stability of these systems.

## Conclusions

In this work, we designed five Ag complexes with strategic variations in anions, the number of heteroatoms and accounted for steric effects. Compound 2 demonstrates notable biological activity, though the reasons behind its superior performance remain unclear, emphasising the need for further investigation into the impact of these structural modifications. The present findings highlight the potential of these compounds and open avenues for exploring their behaviour in solution and the mechanisms driving their bioactivity. Our results suggest a promising pathway for developing new drug candidates for innovative biological applications, with ongoing efforts in our laboratories to further understand, refine, and enhance this activity.

## Data availability

Crystal structures have been deposited in Cambridge Structural Database (CSD) – managed by the Cambridge Crystallographic Data Centre (CCDC). Crystallographic data for L2, 2, 3, 4 and 5 have been deposited at the CCDC under 2388779–2388783 and can be obtained from https://www.ccdc.cam.ac.uk/.

## Author contributions

GEK devised the project. AE synthesised and characterised the ligands and complexes and performed the NMR studies. GAJ contributed to the synthesis of the ligands and one complex. GEK, GJT and SJC crystallographic data. CNB and SKH performed the biological studies. All authors contributed to the preparation of the article.

## Conflicts of interest

There are no conflicts to declare.

## Supplementary Material

RA-015-D5RA01072A-s001

RA-015-D5RA01072A-s002

## References

[cit1] Islam S. U. , Hashmi A. A. and Khan S. A., Advances in Metallodrugs: Preparation and Applications in Medicinal Chemistry, John Wiley & Sons, 2020

[cit2] Sanchez-Cano C., Hannon M. J. (2009). Dalton Trans..

[cit3] Mjos K. D., Orvig C. (2014). Chem. Rev..

[cit4] Anthony E. J., Bolitho E. M., Bridgewater H. E., Carter O. W. L., Donnelly J. M., Imberti C., Lant E. C., Lermyte F., Needham R. J., Palau M., Sadler P. J., Shi H., Wang F.-X., Zhang W.-Y., Zhang Z., Anthony E. J., Bolitho E. M., Bridgewater H. E., Carter O. W. L., Donnelly J. M., Imberti C., Lant E. C., Lermyte F., Needham R. J., Palau M., Sadler P. J., Shi H., Wang F.-X., Zhang W.-Y., Zhang Z. (2020). Chem. Sci..

[cit5] Tan S., Yan Y., Lee P., Lim K. (2010). Future Med. Chem..

[cit6] Liu W., Gust R. (2013). Chem. Soc. Rev..

[cit7] Şahin-Bölükbaşı S., Şahin N. (2019). J. Organomet. Chem..

[cit8] Kaps L., Biersack B., Müller-Bunz H., Mahal K., Münzner J., Tacke M., Mueller T., Schobert R. (2012). J. Inorg. Biochem..

[cit9] Porchia M., Pellei M., Marinelli M., Tisato F., Del Bello F., Santini C. (2018). Eur. J. Med. Chem..

[cit10] Haque R. A., Ghdhayeb M. Z., Budagumpi S., Ahamed M. B. K., Majid A. M. S. A. (2016). RSC Adv..

[cit11] Hackenberg F., Müller-Bunz H., Smith R., Streciwilk W., Zhu X., Tacke M. (2013). Organometallics.

[cit12] Allison S. J., Sadiq M., Baronou E., Cooper P. A., Dunnill C., Georgopoulos N. T., Latif A., Shepherd S., Shnyder S. D., Stratford I. J. (2017). Cancer Lett..

[cit13] Büssing R., Bublitz A., Karge B., Brönstrup M., Strowig T., Ott I. (2024). JBIC, J. Biol. Inorg. Chem..

[cit14] Sun H. , eMagRes, 2007

[cit15] Fan Y., Huang Z., Wang X., Ma Y., Li Y., Yang S., Shi Y., Fan Y., Huang Z., Wang X., Ma Y., Li Y., Yang S., Shi Y. (2020). Molecules.

[cit16] Medici S., Peana M., Nurchi V. M., Zoroddu M. A. (2019). J. Med. Chem..

[cit17] Medici S., Peana M., Crisponi G., Nurchi V. M., Lachowicz J. I., Remelli M., Zoroddu M. A. (2016). Coord. Chem. Rev..

[cit18] Njogu E. M., Omondi B., Nyamori V. O. (2017). J. Mol. Struct..

[cit19] Esarev I. V., Karge B., Zeng H., Lippmann P., Jones P. G., Schrey H., Brönstrup M., Ott I. (2024). ACS Infect. Dis..

[cit20] Kalinowska-Lis U., Felczak A., Chęcińska L., Zawadzka K., Patyna E., Lisowska K., Ochocki J. (2015). Dalton Trans..

[cit21] Patl S., Tacke M. (2011). Metallomics.

[cit22] Wang M., Li G., Jiang G., Cai J., Liu Z., Huang R., Huang X., Wang H. (2024). J. Med. Chem..

[cit23] Zhang S., Zhong X., Yuan H., Guo Y., Song D., Qi F., Zhu Z., Wang X., Guo Z. (2020). Chem. Sci..

[cit24] Monteiro D. C., Phillips R. M., Crossley B. D., Fielden J., Willans C. E. (2012). Dalton Trans..

[cit25] Ray S., Mohan R., Singh J. K., Samantaray M. K., Shaikh M. M., Panda D., Ghosh P. (2007). J. Am. Chem. Soc..

[cit26] Medvetz D. A., Hindi K. M., Panzner M. J., Ditto A. J., Yun Y. H., Youngs W. J. (2008). Met. Base. Drugs.

[cit27] Patil S., Deally A., Gleeson B., Müller-Bunz H., Paradisi F., Tacke M. (2011). Metallomics.

[cit28] Hindi K. M., Panzner M. J., Tessier C. A., Cannon C. L., Youngs W. J. (2009). Chem. Rev..

[cit29] Fu R., Zhao B., Chen M., Fu X., Zhang Q., Cui Y., Hu X., Zhou W. (2023). Med. Oncol..

[cit30] Mao X., Xu S., Wang H., Xiao P., Li S., Wu J., Sun J., Jin C., Shen M., Shi Y. (2024). Cancer Cell Int..

[cit31] Coffetti G., Moraschi M., Facchetti G., Rimoldi I. (2023). Molecules.

[cit32] Liang X., Luan S., Yin Z., He M., He C., Yin L., Zou Y., Yuan Z., Li L., Song X. (2018). Eur. J. Med. Chem..

[cit33] Kenny R. G., Marmion C. J. (2019). Chem. Rev..

[cit34] Hannon M. J., Painting C. L., Plummer E. A., Childs L. J., Alcock N. W. (2002). Chem.–Eur. J..

[cit35] Zibaseresht R., Hartshorn R. M. (2005). Dalton Trans..

[cit36] Carrión M. C., Ortiz I. M., Jalon F. A., Manzano B. R., Rodríguez A. M., Elguero J. (2011). Cryst. Growth Des..

[cit37] Loukopoulos E., Abdul-Sada A., Viseux E. M., Lykakis I. N., Kostakis G. E. (2018). Cryst. Growth Des..

[cit38] Bollikolla H. B., Boddapati S. M., Thangamani S., Mutchu B. R., Alam M. M., Hussien M., Jonnalagadda S. B. (2023). J. Heterocycl. Chem..

[cit39] Briguglio I., Piras S., Corona P., Gavini E., Nieddu M., Boatto G., Carta A. (2015). Eur. J. Med. Chem..

[cit40] Kassab A. E. (2023). Arch. Pharm..

[cit41] Yuan J., Zhong Y., Li S., Zhao X., Luan G., Zhao Z., Huang J., Li H., Xu Y. (2013). Chin. J. Chem..

[cit42] Bajaj K. and Sakhuja R., The Chemistry of Benzotriazole Derivatives: A Tribute to Alan Roy Katritzky, 2016, pp. 235–283

[cit43] Tomas F., Catalan J., Perez P., Elguero J. (1994). J. Org. Chem..

[cit44] Burke-Laing M., Laing M. (1976). Acta Crystallogr., Sect. B.

[cit45] Li Q., Liu G., Wang N., Yin H., Li Z. (2020). J. Heterocycl. Chem..

[cit46] Loukopoulos E., Kostakis G. E. (2019). Coord. Chem. Rev..

[cit47] Smith J. R. L., Sadd J. S. (1975). J. Chem. Soc., Perkin Trans. 1.

[cit48] Kostakis G. E. (2023). Dalton Trans..

[cit49] Richardson C., Steel P. J. (2003). Dalton Trans..

[cit50] Wang F., Wu X.-Y., Zhao Z.-G., Zhang Q.-S., Xie Y.-m., Yu R., Lu C.-Z. (2010). Inorg. Chim. Acta.

[cit51] Pandey S., Mandal T., Mandal S. K. (2022). Polyhedron.

[cit52] Wang F., Wu X.-Y., Yu R.-M., Zhao Z.-G., Lu C.-Z. (2009). J. Coord. Chem..

[cit53] Huang R., Yang Y., Wang D.-S., Zhang L., Wang D. (2018). Org. Chem. Front..

[cit54] Xu Z., Wang D. S., Yu X., Yang Y., Wang D. (2017). Adv. Synth. Catal..

[cit55] Pandey S., Mandal T. (2021). Eur. J. Inorg. Chem..

[cit56] Sampani S. I., Zdorichenko V., Devonport J., Rossini G., Leech M. C., Lam K., Cox B., Abdul-Sada A., Vargas A., Kostakis G. E. (2021). Chem.–Eur. J..

[cit57] Pandey S., Mandal T., Singh V. (2020). ChemistrySelect.

[cit58] Castro J., Ferraro V., Bortoluzzi M. (2022). New J. Chem..

[cit59] Canales S., Crespo O., Fortea A., Gimeno M. C., Jones P. G., Laguna A. (2002). J. Chem. Soc., Dalton Trans..

[cit60] Argyle V. J., Woods L. M., Roxburgh M., Hanton L. R. (2013). CrystEngComm.

[cit61] Leoni P., Vichi E., Lencioni S., Pasquali M., Chiarentin E., Albinati A. (2000). Organometallics.

[cit62] Kintzel S., Eckhardt K., Getzschmann J., Bon V., Grothe J., Kaskel S. (2020). Eur. J. Inorg. Chem..

[cit63] Liu X. Y., Zhu H. L. (2005). Synth. React. Inorg., Met.-Org., Nano-Met. Chem..

[cit64] Sun D., Zhang N., Xu Q.-J., Wei Z.-H., Huang R.-B., Zheng L.-S. (2011). Inorg. Chim. Acta.

[cit65] Shungu D. L., Weinberg E., Gadebusch H. H. (1983). Antimicrob. Agents Chemother..

[cit66] Milionis I., Banti C. N., Sainis I., Raptopoulou C. P., Psycharis V., Kourkoumelis N., Hadjikakou S. K., Milionis I., Banti C. N., Sainis I., Raptopoulou C. P., Psycharis V., Kourkoumelis N., Hadjikakou S. K. (2018). J. Biol. Inorg. Chem..

[cit67] Stathopoulou M.-E. K., Banti C. N., Kourkoumelis N., Hatzidimitriou A. G., Kalampounias A. G., Hadjikakou S. K. (2018). J. Inorg. Biochem..

[cit68] Banti C., Kapetana M., Papachristodoulou C., Raptopoulou C., Psycharis V., Zoumpoulakis P., Mavromoustakos T., Hadjikakou S. (2021). Dalton Trans..

[cit69] Banti C. N., Hadjikakou S. K. (2024). Antibiotics.

[cit70] Coles S. J., Gale P. A. (2012). Chem. Sci..

