# Supplementary material for: Investigating the antimicrobial activity of 1-heteroaryl benzotriazole silver compounds
Source: RSC Adv. 2025 Apr 11;15(15):11431–40. doi: 10.1039/d5ra01072a (PMC11987083; doi:10.1039/d5ra01072a)
Supplement: RA-015-D5RA01072A-s001 [file RA-015-D5RA01072A-s001.pdf]

Supplementary Information (SI) for RSC Advances.
This journal is © The Royal Society of Chemistry 2025

Investigating antimicrobial activity of 1-heteroaryl benzotriazole silver compounds

Ahmed Elzein,[a] Ghadah Abdullah S. Al Jomeh,[a] Graham J. Tizzard,[b] Simon J. Coles,[b] Christina N. Banti,*[c] Sotiris K. Hadjikakou,*[c,d] and George E. Kostakis*[a]

[a] Department of Chemistry, School of Life Sciences, University of Sussex, Brighton BN1 9QJ, UK **E-mail:** G.Kostakis@sussex.ac.uk
[b] UK National Crystallography Service, Chemistry, University of Southampton, UK
[c] Biological Inorganic Chemistry laboratory, Department of Chemistry, University of Ioannina, 45110 Ioannina, Greece **E-mail:** cbanti@uoi.gr, shadjika@uoi.gr
[d] University Research Center of Ioannina (URCI), Institute of Materials Science and Computing, Ioannina, Greece

Contents

1. Materials 2
2. General Scientific methods and instrumentation 2
3. Single crystal X-ray diffraction studies 2
4. Elemental analyses. 4
5. Solution studies 5
5.1. NMR data 5
5.2. ESI-MS 10
5.3. Cyclic voltammetry 10
6. Biological studies 11
7. Synthetic procedures 15
7.1. Ligand synthesis 15
7.2. Complex Synthesis 17
8. Copies of all characterisation data 19
Ligand L1 19
Ligand L2 22
Ligand L3 25
*Compound **1*** 28
*Compound **2*** 31
*Compound **3*** 37
*Compound **4*** 41
*Compound **5*** 46
9. References 51

1. **Materials**

All reagents were purchased from Sigma Aldrich, Fluorochem, Tokyo Chemical Industry, Apollo Scientific, Fischer Scientific or Alfa Aesar and used without further purification. Experiments were performed under aerobic or under nitrogen conditions. The petroleum ether used was of the fraction that boils between 40 and 60 °C.

2. **General Scientific methods and instrumentation**

NMR spectra were recorded with a Varian VNMRS 600 and Varian VNMRS 400 at 30 °C on solution-state samples in Chloroform-d or d^6-DMSO. Chemical shifts are quoted in parts per million (ppm). Coupling constants (J) are recorded in units of Hz. HRMS (ESI-FTMS) data was obtained with a Bruker Daltonics Fourier Transform (FTMS) Apex II spectrometer with electrospray ionisation (ESI) using methanol and d^6-DMSO as solvents. ESI-MS data were obtained on a VG Auto spec Fissions instrument (EI at 70 eV), with high accuracy of four decimal places and carried out by Dr Ramon Gonzalez at the University of Sussex. Molecular ions are reported as mass/charge (m/z) ratios. UV–Vis measurements (280-900 nm & 320 – 1100 nm) were performed at room temperature (25˚C) using a Thermo Scientific Evolution 300 UV-Vis spectrophotometer equipped with 5mm path length quartz cells, and the collected data were processed using the Vision Pro software. Elemental analysis was performed at London Metropolitan University. IR spectra were recorded over the range of 4000–440 cm^{-1} on a PerkinElmer Spectrum One FT-IR spectrometer fitted with a UATR polarisation accessory.

3. **Single crystal X-ray diffraction studies**

Single crystal diffraction data for **2** and **4** were collected at the National Crystallography Service, University of Southampton.[1] Suitable crystals were mounted on a MITIGEN holder in oil on a Rigaku FRE+ diffractometer with Arc)Sec VHF Varimax confocal mirrors, an AFC12 goniometer and HyPix 6000HE detector and data were collected at T = 100(2)K. Data for **3** were collected at Diamond Light Source Beamline I19-1 at T = 100(2)K. Single crystal diffraction data for **L2** and **5** were collected at University of Sussex in a Rigaku rotating anode diffractometer at T = 100(2)K for **L2** and 124(5)K for **5**. The data were processed with CrysAlisPro and solved by intrinsic phasing methods with SHELXT.[2] All crystal structures were then refined on Fo^2 by full-matrix least-squares refinements using SHELXL[2] or olex2.refine.[3] All non-hydrogen atoms were refined anisotropically. Hydrogen atom positions were calculated geometrically and refined using the riding model. Data for **3** were solved in space group *Ia* and refined as a 2-component inversion twin (ca. 55:45) and thermal restraints (RIGU) were applied to all non-hydrogen atoms. Data for **4** was processed and refined as a 2-component non-merohedral twin (ca.57:43). Thermal restraints (RIGU) were applied to selected atoms (Ag1, N1-N3, C1, C6 and C7). Geometric/crystallographic calculations were performed using PLATON,[3] Olex2,[4] and WINGX[5] packages; Images and video were generated using CrystalMaker®: a crystal and molecular structures program for Mac and Windows. CrystalMaker Software Ltd, Oxford, England (www.crystalmaker.com). Structures have been given CCDC deposition numbers 2388779- 2388783.

Table S1. Crystal data and structure refinement for compounds **L2**, **2** – **5.**

Identification code	**L2**	**2**	**3**	**4**	**5**
Empirical formula	$C_{15}H_{10}N_4$	$C_{24}H_{16}Ag_2F_6N_8O_6S_2$	$C_{30}H_{20}AgN_9O_3$	$C_{32}H_{20}Ag_2F_6N_8O_6S_2$	$C_{11}H_7AgF_3N_5O_3S$
Formula weight	246.27	906.304	662.420	1006.42	454.15
Temperature/K	100	100(2)	100(2)	100(2)	124(5)
Crystal system	monoclinic	triclinic	monoclinic	triclinic	monoclinic
Space group	$P2_1/c$	P-1	Ia	P-1	$P2_1/c$
a/Å	13.2953(10)	7.94750(10)	26.5565(8)	7.9014(5)	8.73200(10)
b/Å	5.5726(3)	9.99190(10)	3.81000(10)	9.4771(5)	8.5637(2)
c/Å	16.8008(9)	10.3949(2)	25.8471(9)	12.3812(12)	18.9845(3)
α/°	90	73.3090(10)	90	107.539(6)	90
β/°	111.958(8)	70.4440(10)	105.206(3)	101.878(6)	100.7140(10)
γ/°	90	69.5970(10)	90	101.752(5)	90
Volume/Å^3	1154.47(13)	715.332(19)	2523.66(14)	829.29(11)	1394.88(4)
Z	4	1	4	1	4
ρ_{calc}g/cm^3	1.417	2.104	1.743	2.015	2.163
μ/mm^{-1}	0.709	1.612	2.541	1.402	13.602
F(000)	512.0	444.0	1337.1	496.0	888.0
Crystal size/mm^3	0.05 × 0.03 × 0.02	0.1 × 0.06 × 0.03	0.17 × 0.009 × 0.007	0.12 × 0.07 × 0.005	0.548 × 0.303 × 0.086
Radiation	Cu Kα (λ = 1.54184)	Mo Kα (λ = 0.71073)	Synchrotron (λ = 1.04020)	Mo Kα (λ = 0.71073)	Cu Kα (λ = 1.54184)
2Θ range for data collection/°	10.932 to 136.266	4.238 to 76.186	4.66 to 79.88	4.702 to 67.376	9.482 to 142.852
Index ranges	-12 ≤ h ≤ 15, -6 ≤ k ≤ 5, -19 ≤ l ≤ 20	-13 ≤ h ≤ 13, -17 ≤ k ≤ 17, -17 ≤ l ≤ 17	-32 ≤ h ≤ 32, -4 ≤ k ≤ 4, -30 ≤ l ≤ 31	-12 ≤ h ≤ 12, -14 ≤ k ≤ 14, -19 ≤ l ≤ 18	-10 ≤ h ≤ 10, -9 ≤ k ≤ 10, -23 ≤ l ≤ 21
Reflections collected	4187	65339	8204	13432	19129
Independent reflections	2052 [R_{int} = 0.0384, R_{sigma} = 0.0488]	7516 [Rint = 0.0412, Rsigma = 0.0201]	4318 [R_{int} = 0.0479, R_{sigma} = 0.0704]	13432 [R_{int} = ?, R_{sigma} = 0.0528]	2702 [R_{int} = 0.0587, R_{sigma} = 0.0296]
Data/restraints/parameters	2052/0/172	7516/16/217	4318/371/389	13432/237/254	2702/0/217
Goodness-of-fit on F^2	1.033	1.036	1.015	1.132	1.028
Final R indexes [I>=2σ (I)]	R_1 = 0.0517, wR_2 = 0.1377	R1 = 0.0220, wR2 = 0.0560	R_1 = 0.0623, wR_2 = 0.1559	R_1 = 0.0961, wR_2 = 0.2264	R_1 = 0.0368, wR_2 = 0.0978
Final R indexes [all data]	R_1 = 0.0589, wR_2 = 0.1458	R1 = 0.0240, wR2 = 0.0572	R_1 = 0.0837, wR_2 = 0.1733	R_1 = 0.1116, wR_2 = 0.2362	R_1 = 0.0390, wR_2 = 0.1003
Largest diff. peak/hole / e Å^{-3}	0.22/-0.27	1.26/-1.17	1.19/-1.23	3.38/-2.14	0.98/-1.09

4. **Elemental analyses.**

Elemental analysis results for all complexes were recorded at London Metropolitan University. The samples, upon crystallisation, were filtered, dried, collected and kept out of light. For compounds **1** and **4**, the analysis was performed for the solid colourless white precipitate that was collected during filtration.

1, Calculated for $C_{24}H_{16}Ag_2F_6N_8O_6S_2$ C, 31.81; H, 1.78; N: 12.36; Found C: 31.90; H: 1.82; N: 12.37.

2, Calculated for $C_{22}H_{16}Ag_2F_6N_{10}O_6$ C, 36.09; H, 2.20; N: 19.13; Found C: 36.08; H: 1.43; N: 18.68.

3, Calculated for $C_{30}H_{20}AgN_9O_3$ C, 54.40; H, 3.04; N: 19.03; Found C: 54.34; H: 2.86; N: 18.26.

4, Calculated for $C_{32}H_{20}Ag_2F_6N_8O_6S_2$ C, 38.19; H, 2.00; N: 11.13; Found C: 37.93; H: 1.62; N: 11.86.

5, Calculated for $C_{22}H_{14}Ag_2F_6N_{10}O_6S_2$ C, 29.09; H, 1.55; N: 15.42; Found C: 29.25; H: 1.32; N: 15.31.

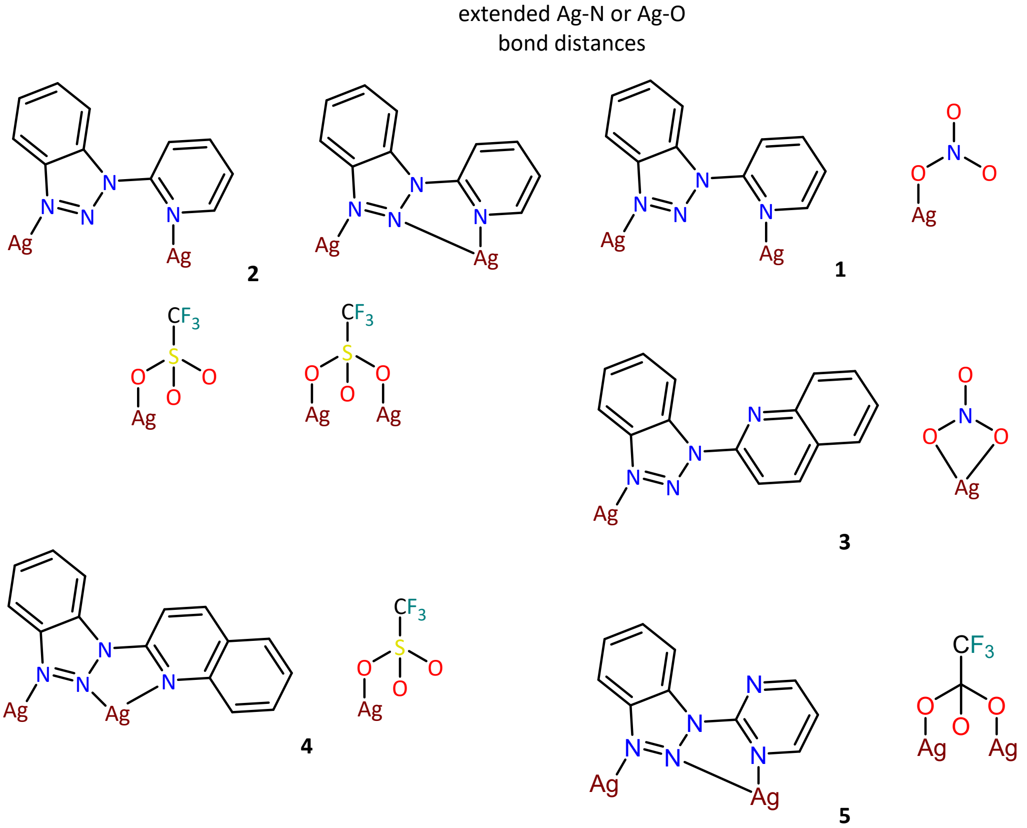

Scheme S1. A schematic representation of the different coordination modes observed in the present Ag family of compounds .

5. Solution studies

5.1. NMR data

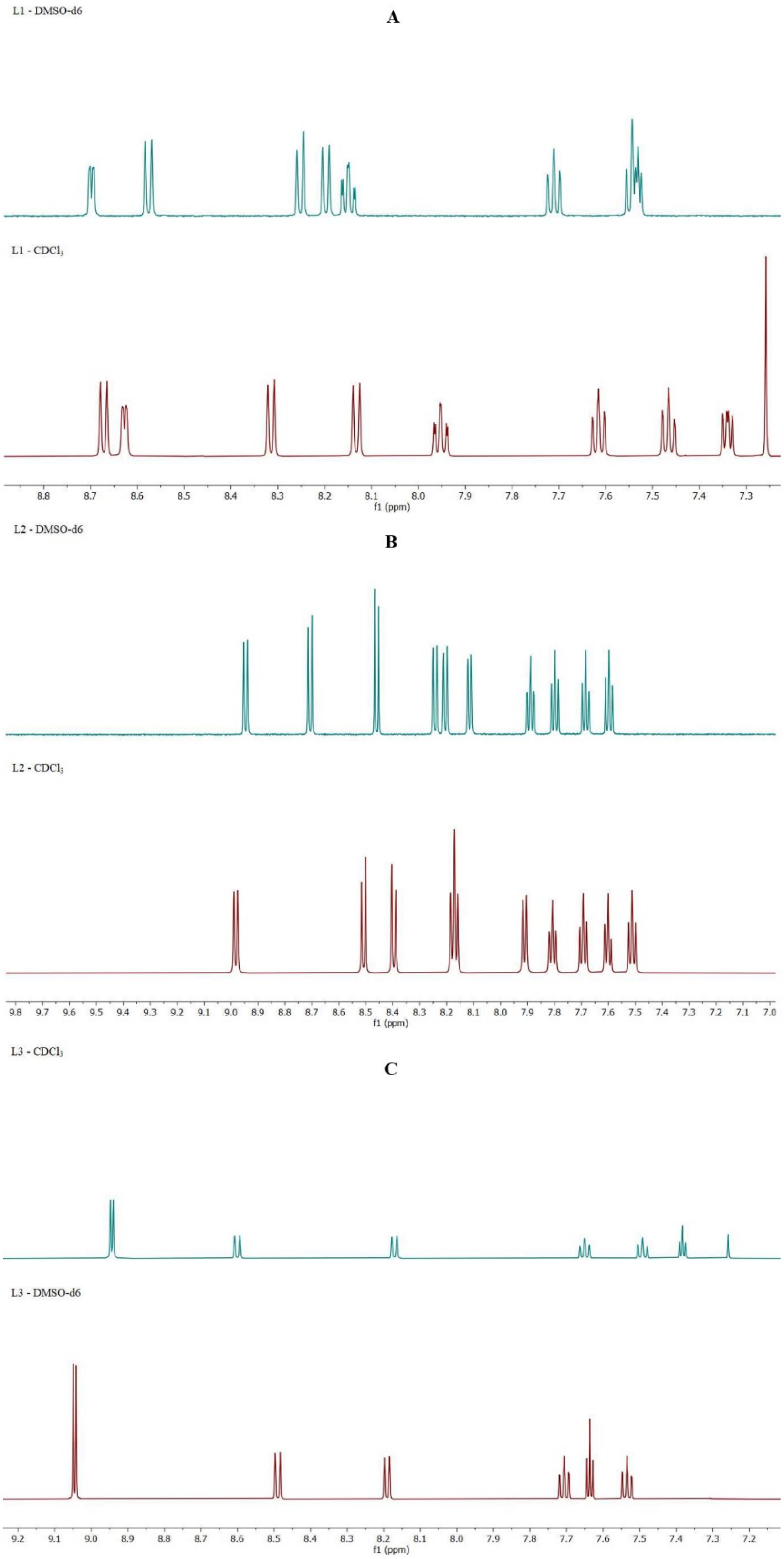

Figure S1. ^{1}H NMR of **L1** (A), **L2** (B), and **L3** (C) in d^6-DMSO vs $CDCl_3$.

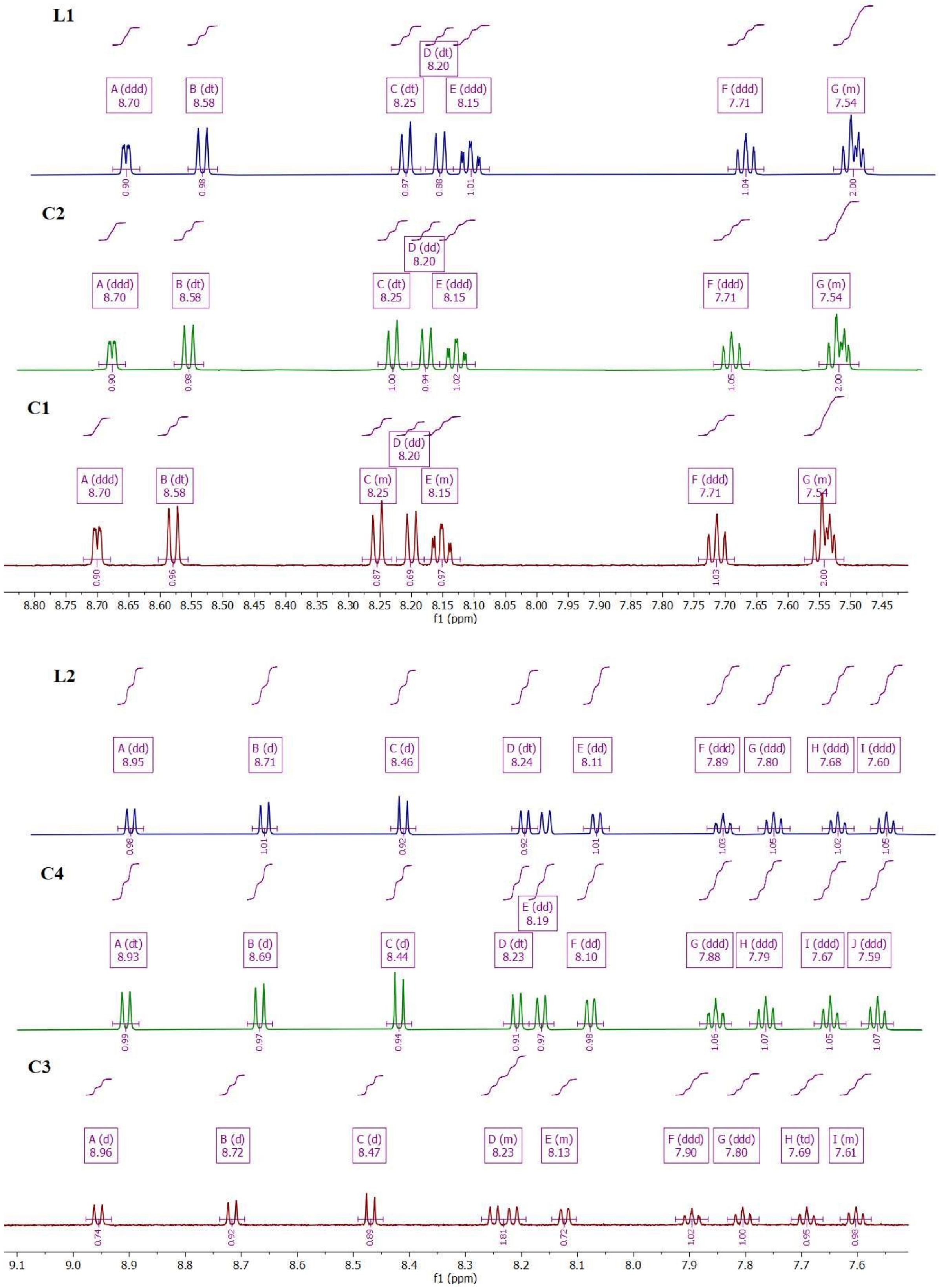

Figure S2. *^{1}H NMR comparison between Ag(**L1**) based complexes (top), Ag(**L2**) based complexes (bottom), and anion effect.*

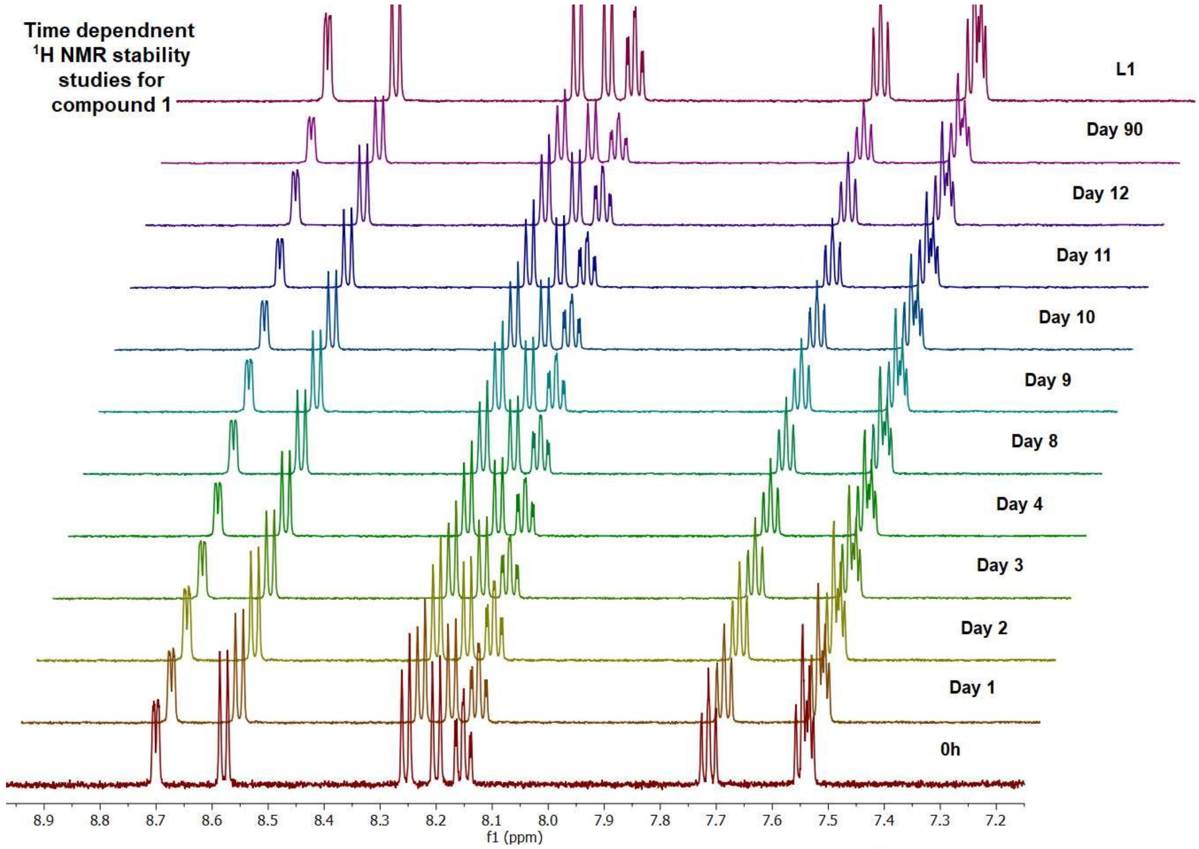
Time dependnent
1H NMR stability
studies for
compound 1
L1
Day 90
Day 12
Day 11
Day 10
Day 9
Day 8
Day 4
Day 3
Day 2
Day 1
0h
8.9 8.8 8.7 8.6 8.5 8.4 8.3 8.2 8.1 8.0 7.9 7.8 7.7 7.6 7.5 7.4 7.3 7.2
f1 (ppm)

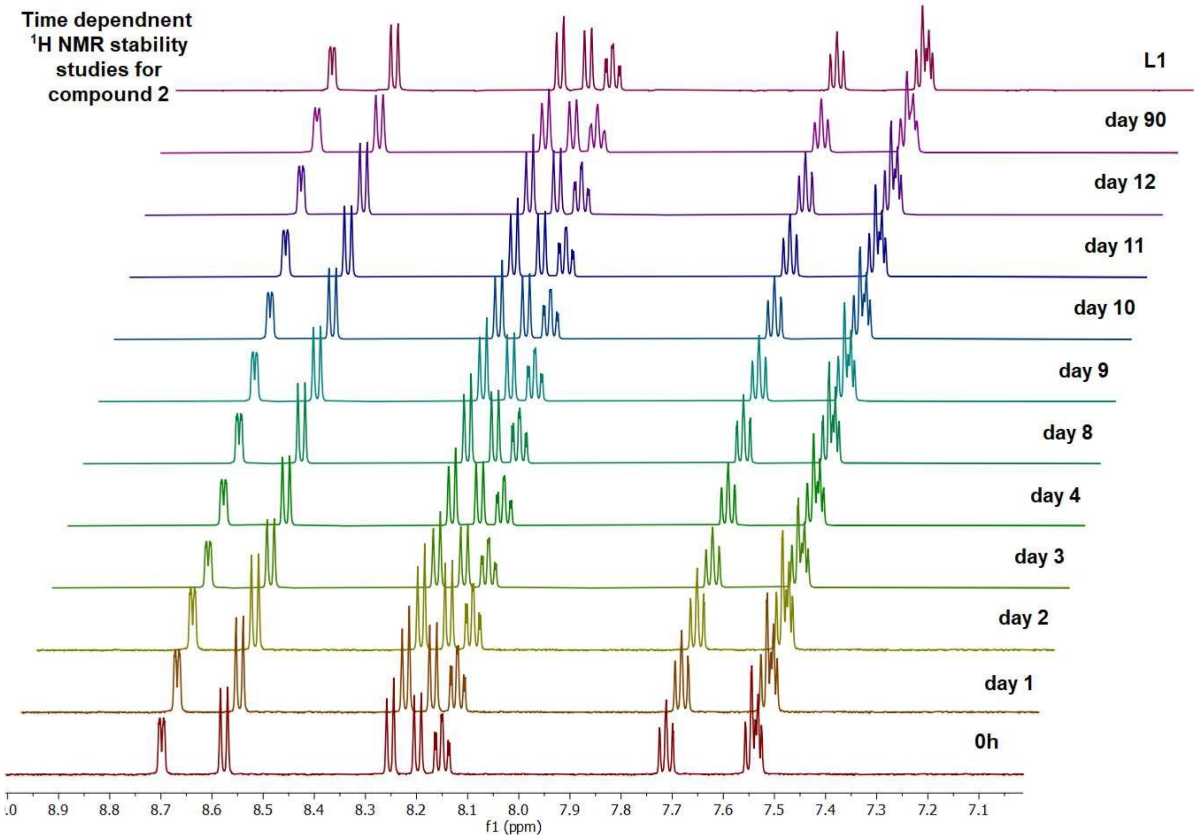
Time dependnent
1H NMR stability
studies for
compound 2
L1
day 90
day 12
day 11
day 10
day 9
day 8
day 4
day 3
day 2
day 1
0h
8.9 8.8 8.7 8.6 8.5 8.4 8.3 8.2 8.1 8.0 7.9 7.8 7.7 7.6 7.5 7.4 7.3 7.2 7.1
f1 (ppm)

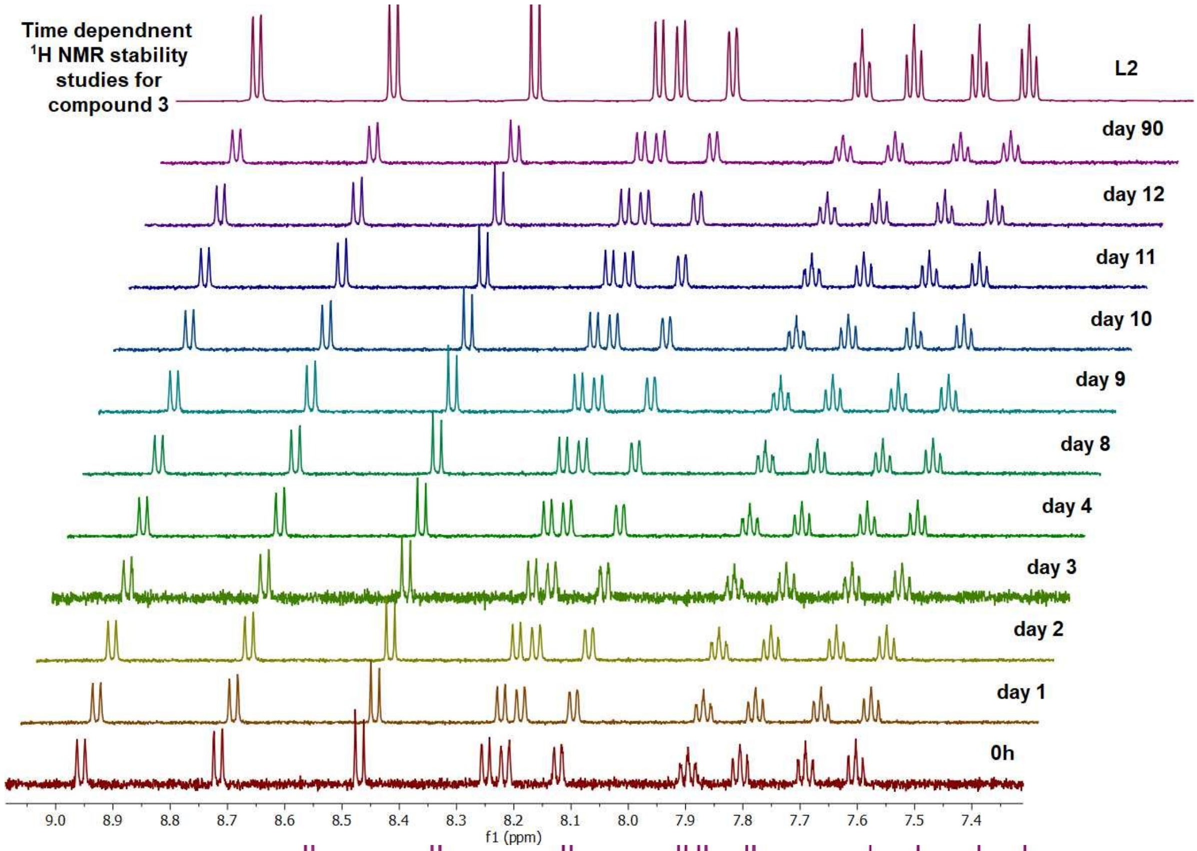
Time dependnent
1H NMR stability
studies for
compound 3
L2
day 90
day 12
day 11
day 10
day 9
day 8
day 4
day 3
day 2
day 1
0h
9.0 8.9 8.8 8.7 8.6 8.5 8.4 8.3 8.2 8.1 8.0 7.9 7.8 7.7 7.6 7.5 7.4
f1 (ppm)

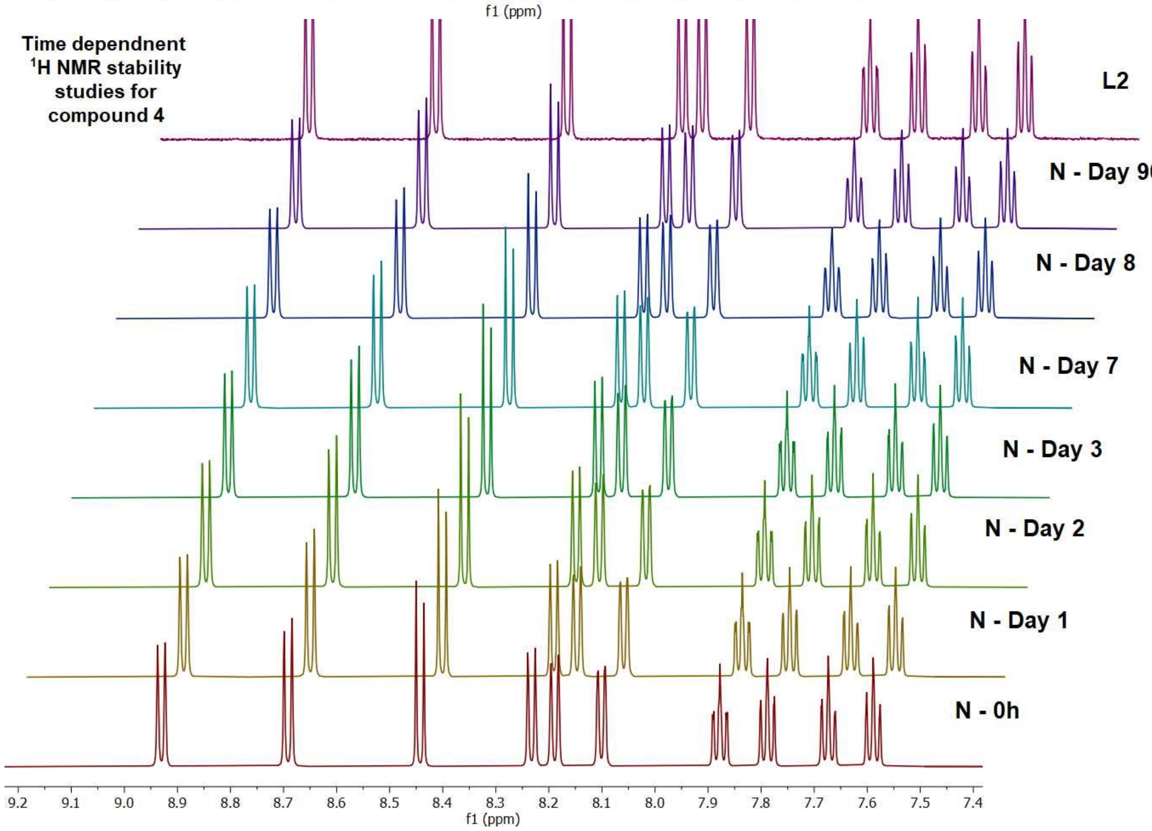
Time dependnent
1H NMR stability
studies for
compound 4
L2
N - Day 90
N - Day 8
N - Day 7
N - Day 3
N - Day 2
N - Day 1
N - 0h
9.2 9.1 9.0 8.9 8.8 8.7 8.6 8.5 8.4 8.3 8.2 8.1 8.0 7.9 7.8 7.7 7.6 7.5 7.4
f1 (ppm)

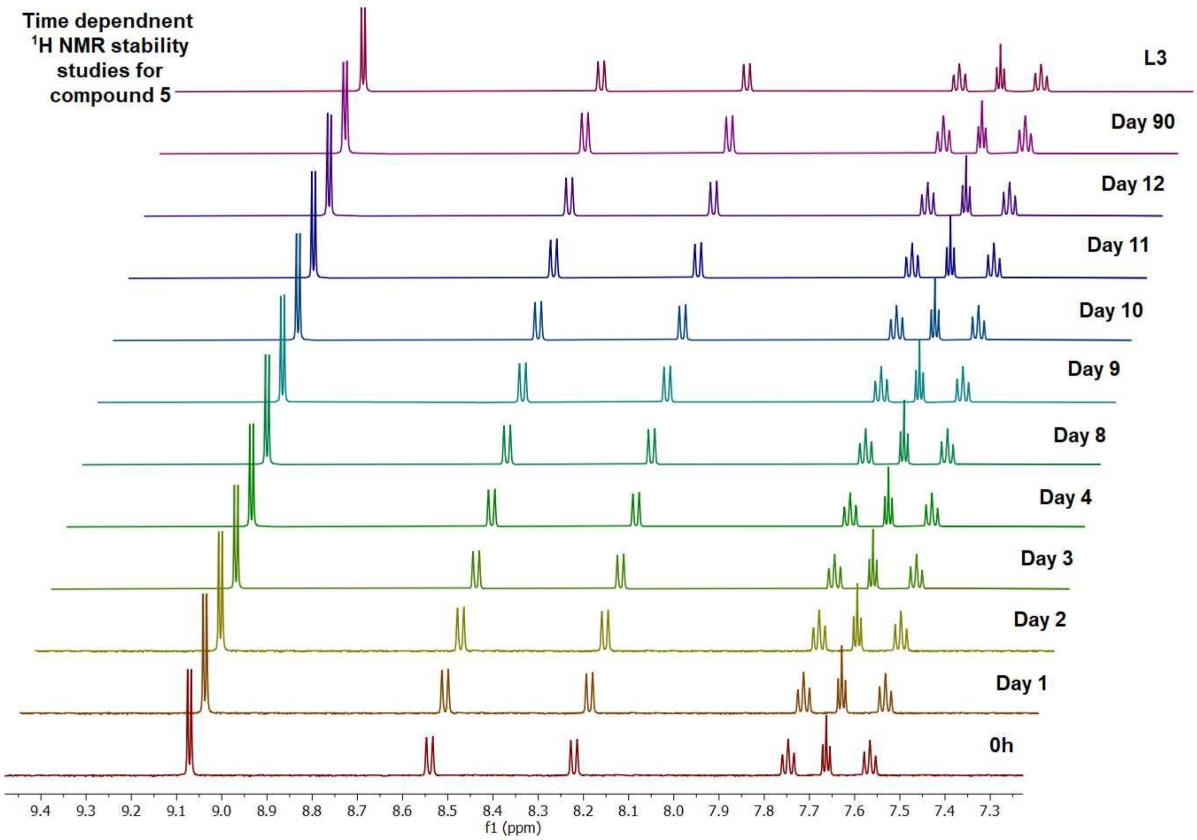

***Figure S3**. Time dependant ^{1}H NMR studies for compounds **1-5**.*

5.2. ESI-MS

Table S2. *Summary of molecular weights calculated from MS for compounds **1 – 5** in d^6-DMSO.*

Entry	Compound	Monomer	Mwt Calc/Found	Dimer	Mwt Calc/Found
**1**	1	$[Ag(\mathbf{L1})]^+$	302.98 / 302.9815	$[Ag_2(\mathbf{L1})_2NO_3]^+$	669.95 / 669.944
		$[Ag(\mathbf{L1})_2]^+$	499.05 / 499.0544		
**2**	2	$[Ag(\mathbf{L1})]^+$	302.98 / 302.9815	$[Ag_2(\mathbf{L1})_2OTf]^+$	756.91 / 756.9095
		$[Ag(\mathbf{L1})_2]^+$	499.05 / 499.0544		
**3**	3	$[Ag(\mathbf{L2})]^+$	353.00 / 352.9968	-	
		$[Ag(\mathbf{L2})_2]^+$	599.09 / 599.0884		
**4**	4	$[Ag(\mathbf{L2})]^+$	353.00 / 352.9968	$[Ag_2(\mathbf{L1})_2OTf]^+$	742.9633 / 742.99
		$[Ag(\mathbf{L2})_2]^+$	599.09 / 599.0884		
**5**	5	$[Ag(\mathbf{L3})]^+$	303.98 / 303.9752	$[Ag_2(\mathbf{L1})_2OTf]^+$	758.90 / 758.9020
		$[Ag(\mathbf{L3})_2]^+$	501.05 / 501.0431		

5.3. Cyclic voltammetry

We attempted to record the CV of compound **1** in the same solvent that the NMR and biological studies were carried out. Thus, we prepared a solution of compound **1** at 0.012 M in DMSO with 0.1 M NH4ClO4 supporting electrolytes; the scan rate was 100 mV/s. Pt disk (1 mm) working electrode; Pt wire counter and Ag wire pseudo-reference. Internal ferrocene is used as a reference.Fc/Fc$^+$ couple at 0.32V; This couple is typically 0.64 V relative to SHE, while Ag/AgCl reference is 0.197 V relative to SHE; thus, in this scan Fc/Fc$^+$ is 0.443 V vs Ag/AgCl. Based on this, the reductive feature is at –0.657 V vs Ag/AgCl. This feature is not observed in an initial reductive sweep; it only follows an oxidative sweep. The initial oxidation process is not observed, but must coincide with solvent breakdown, the onset of which is 1.603 V relative to Ag/AgCl. Data are though inconclusive. Attempts to repeat with NH_4PF_6 supporting electrolyte (to afford a more positive window) were thwarted by instantaneous decomplexation upon adding the analyte, affording colloidal silver.

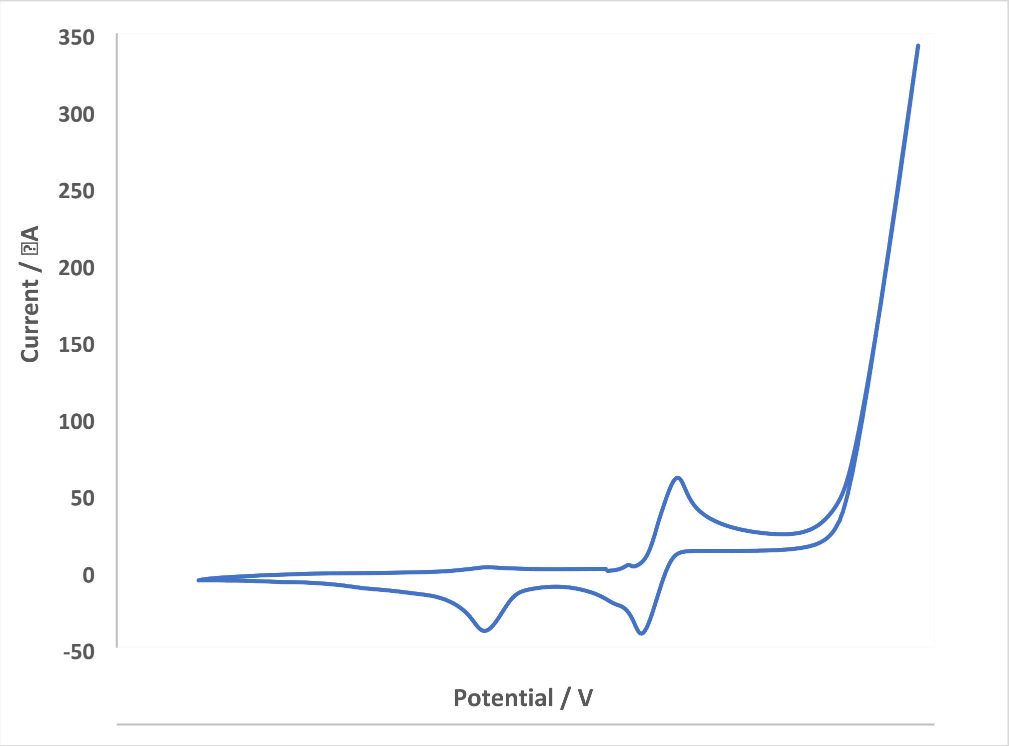

***Figure S4**. CV of compound **1** in DMSO. .*

6. Biological studies

Bacterial Strains: For the antibacterial experiments the strain of *Staphylococcus epidermidis* (ATCC® 14990™), *Staphylococcus aureus* subsp. *aureus* (ATCC® 25923™), *P. aeruginosa and Escherichia coli Dh5a* (*E. coli*) were used.

Antibacterial effects of compounds on the growth of microbial strains: This study was performed according to standard procedure which is also described elsewhere [1-3]. Briefly, the bacterial strains were streaked onto in trypticase soy agar. The plates were incubated for 18-24 h at 37 °C. Three to five isolated colonies are selected of the same morphological appearance from the fresh agar plate using a sterile loop and transfer into a tube containing 2 mL of sterile saline solution. The optical density at 620 nm is adjusted to 0.1 which corresponds to 10^8 cfu/mL. For the evaluation of MIC the inoculum size for broth dilution is 5×10^5 cfu/mL. The total volume of the culture solution treated by compounds and ligands, as well as the total volume of the positive and negative control was 2 mL. The range of concentrations of silver(I) complexes and their ligands is 20-250 μM. The growth is assessed after incubation for 20 hrs.

For the evaluation of MBC, the bacteria were initially cultivated in the presence of compounds and ligands, in broth culture for 20 hrs. The MBC values were determined in duplicate, by subculturing 4 μL of the broth an agar plate.[1-5]

The study of IZ agar plates were inoculated with a standardized inoculum (10^8 cfu/mL) of the tested microorganism. Filter paper disks (9 mm in diameter), which have been previously soaked by compounds and ligands (1 mM), were placed on the agar surface. The Petri dishes were incubated for 20 hrs.

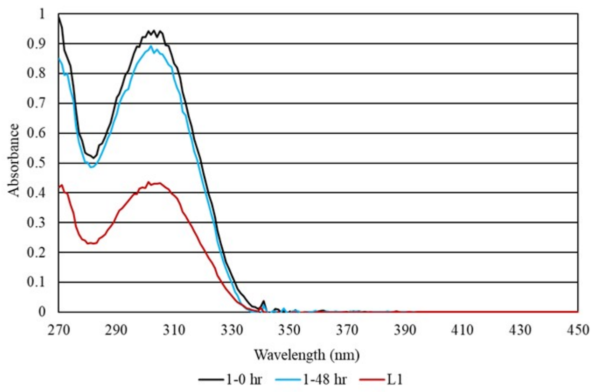

(A)

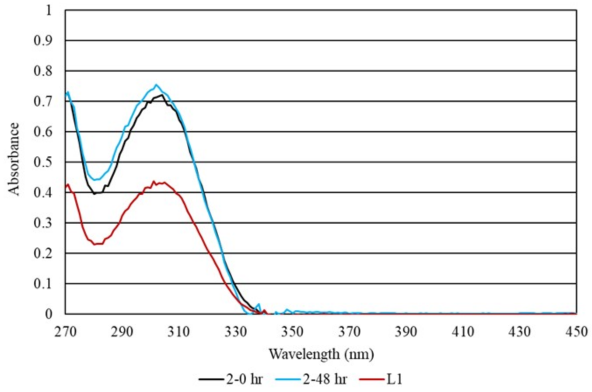

(B)

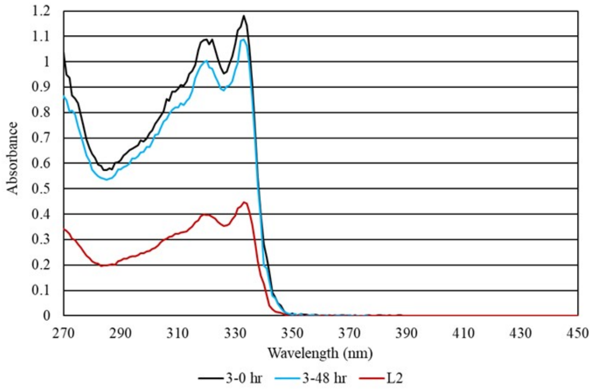

(C)

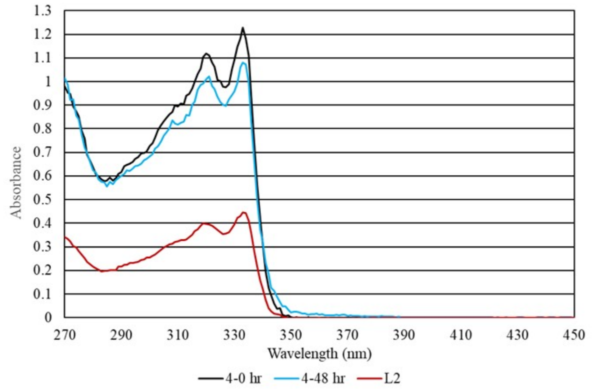

(D)

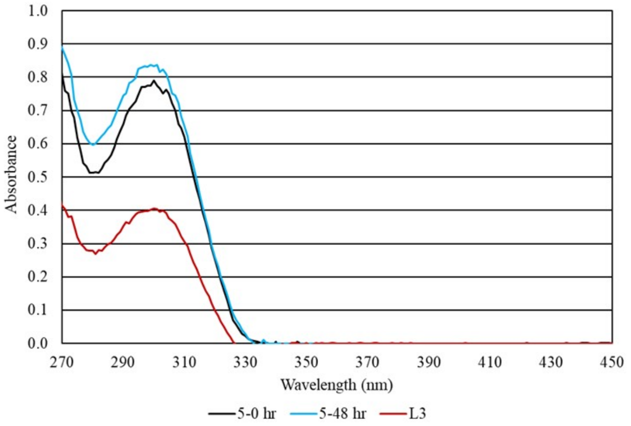

(E)

Figure S5 UV spectra of the compounds **1-5** and after 48 hr in DMSO solution (5×10^{-5} M), and the corresponding of ligands L1, L2 and L3 (5×10^{-5} M)

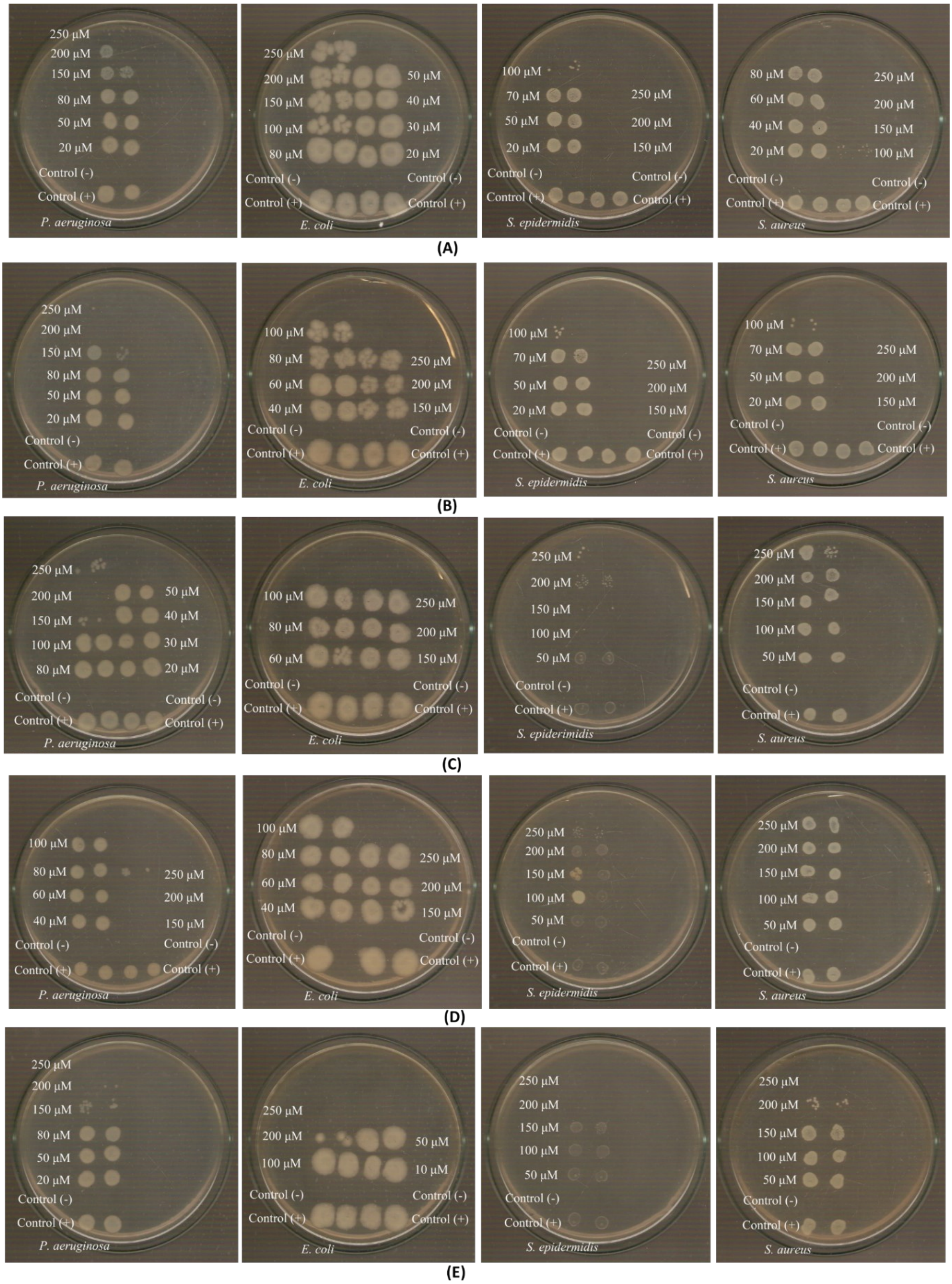

Figure S6. Minimum bactericidal concentration of compounds **1** (**A**), **2** (**B**), **3** (**C**), **4** (**D**) and **5** (**E**) against *P. aeruginosa*, *E. coli*, *S. epidermidis* and *S. aureus.*

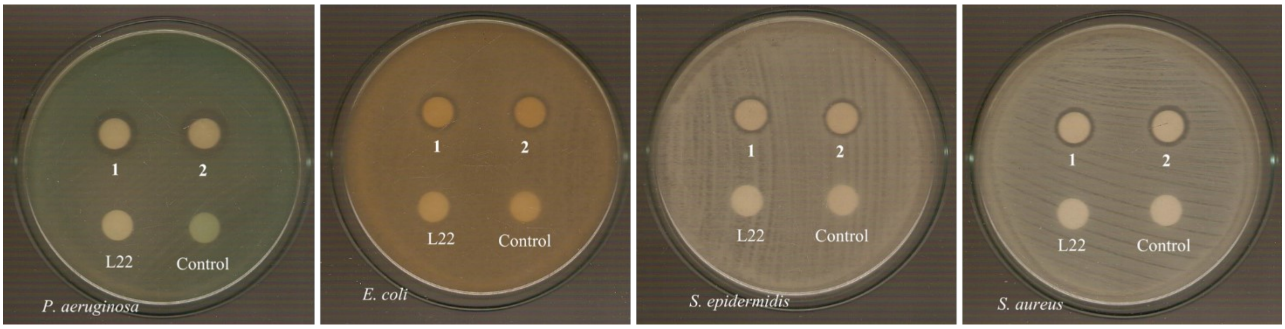

(A)

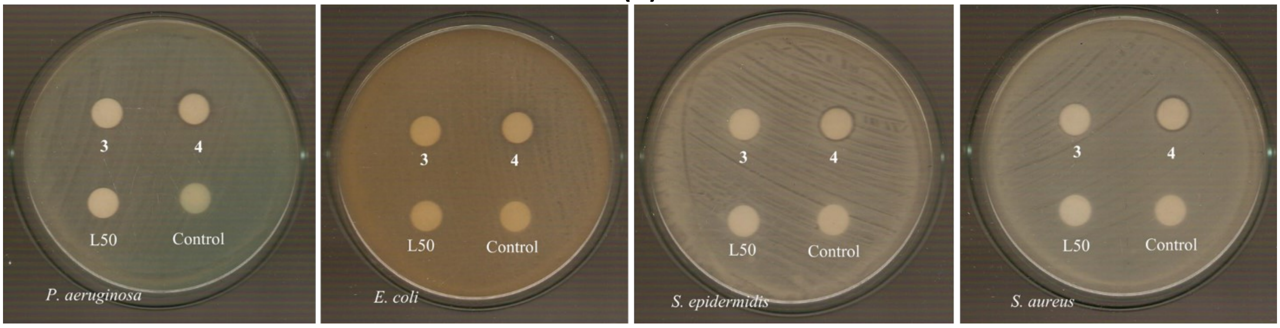

(B)

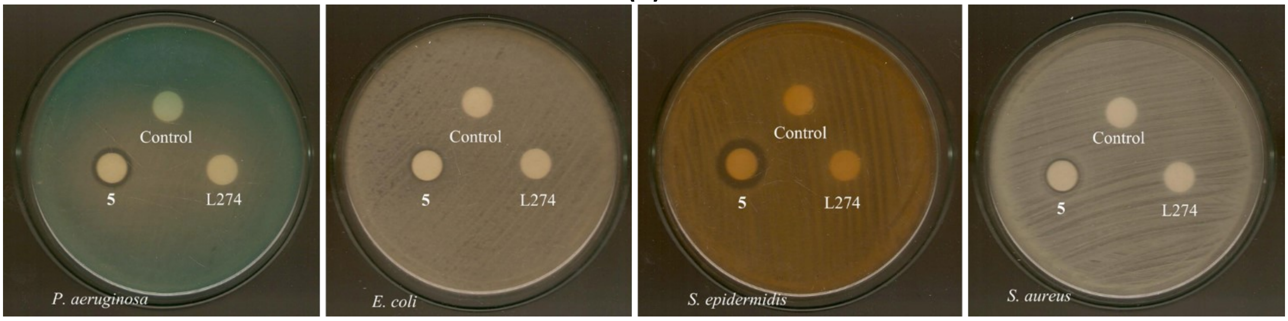

(C)

Figure S7. IZs developed in agar plates of *P. aeruginosa*, *E. coli*, *S. epidermidis* and *S. aureus* for **1** and **2** (**A**), **3** and **4** (**B**) and **5** (**C**) at 1 mM.

7. Synthetic procedures

7.1. Ligand synthesis

Following already established protocols,[6-9] the synthesis of 1-heteroaryl substituted benzotriazole ligands can be achieved in a one pot, high yield reaction, using readily available chemicals and avoiding column chromatography.[10]

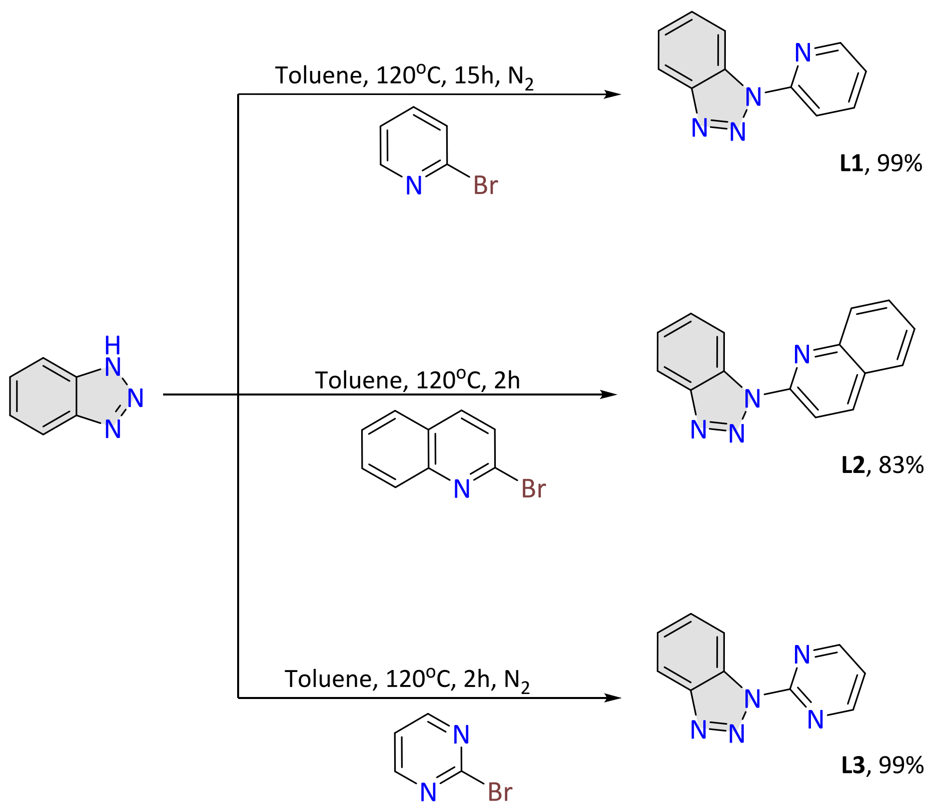

Scheme S2. *Synthetic protocol for benzotriazole derivatives.*

Ligand L1. ^{1}H NMR (600 MHz, Chloroform-*d*) δ 8.67 (dd, *J* = 8.4, 1.0 Hz, 1H), 8.68 – 8.61 (m, 1H), 8.31 (dt, *J* = 8.2, 1.0 Hz, 1H), 8.13 (dt, *J* = 8.4, 1.0 Hz, 1H), 7.95 (ddd, *J* = 8.1, 7.3, 1.8 Hz, 1H), 7.62 (ddd, *J* = 8.3, 7.0, 1.1 Hz, 1H), 7.47 (ddd, *J* = 8.2, 6.9, 1.0 Hz, 1H), 7.34 (ddd, *J* = 7.4, 4.9, 1.1 Hz, 1H).; ^{13}C NMR (151 MHz, Chloroform-*d*) δ 148.36, 146.78, 138.86, 128.80, 124.91, 122.30, 119.80, 114.81, 114.50. (HRMS + TOF MS ES+) calculated $C_{11}H_8N_4$: 196.21, observed: 197.0834.

Ligand L2. ^{1}H NMR (600 MHz, Chloroform-*d*) δ 8.98 (d, *J* = 8.3 Hz, 1H), 8.51 (d, *J = 8.8 Hz,* 1H), 8.40 (d, *J = 8.8 Hz,* 1H), 8.17 (t, *J* = 7.8 Hz, 2H), 7.91 (dd, *J* = 8.1, 1.4 Hz, 1H), 7.81 (ddd, *J* = 8.4, 6.9, 1.4 Hz, 1H), 7.69 (ddd, *J* = 8.2, 7.0, 1.1 Hz, 1H), 7.63 – 7.57 (m, 1H), 7.51 (ddd, *J = 8.2, 7.20, 1.2 Hz, 1H).* ^{13}C NMR (151 MHz, Chloroform-*d*) δ 150.48, 146.55, 139.25, 131.65, 130.56, 129.01, 128.82, 127.82, 127.09, 126.70, 125.20, 119.86, 115.45, 113.46. (HRMS + TOF MS ES+) calculated $C_{11}H_8N_4$: 246.27, observed: 247.0990.

Ligand L3. ^{1}H NMR (600 MHz, Chloroform-*d*) δ 8.67 (dd, *J* = 8.4, 1.0 Hz, 1H), 8.68 – 8.61 (m, 1H), 8.31 (dt, *J* = 8.2, 1.0 Hz, 1H), 8.13 (dt, *J* = 8.4, 1.0 Hz, 1H), 7.95 (ddd, *J* = 8.1, 7.3, 1.8 Hz, 1H), 7.62 (ddd, *J* = 8.3, 7.0, 1.1 Hz, 1H), 7.47 (ddd, *J* = 8.2, 6.9, 1.0 Hz, 1H), 7.34 (ddd, *J* = 7.4, 4.9, 1.1 Hz, 1H).; ^{13}C NMR (151 MHz, Chloroform-*d*) δ 160.10, 155.93, 146.38, 131.68, 129.89, 125.86, 121.05, 120.16, 115.00. (HRMS + TOF MS ES+) calculated $C_{11}H_8N_4$: 197.20, observed: 198.0806.

7.2. Complex Synthesis

Following already established protocols,[11-17] the synthesis of compounds **1** - **5** can be achieved in a one pot,[18-20] high yield reaction, using readily available chemicals.[21-23]

General complex synthesis

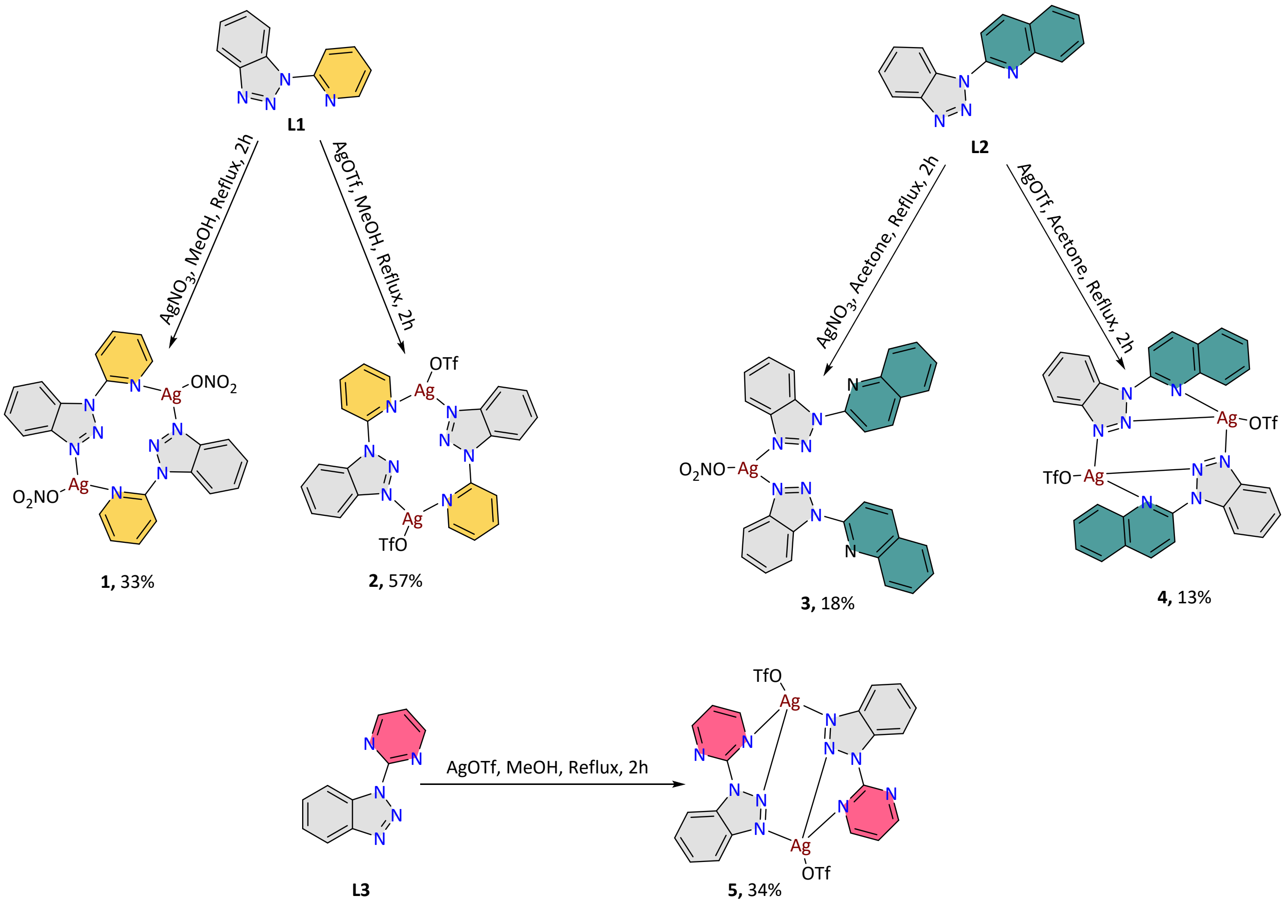

Scheme S3. *Synthetic protocol for compounds **1-5**.*

Compound 1 was prepared by dissolving $AgNO_3$ (85 mg, 0.5 mmol) in MeOH (5 ml) in one vial, and an equimolar amount of **L1** (98 mg, 0.5 mmol) was dissolved in MeOH (5 ml) in a different vial. The two solutions were combined by slow addition of the ligand solution to the metal solution and refluxed for 2 hours while being protected from light. The mixture was left to cool down to room temperature, then filtered. The solution was left to form crystals by slow evaporation (121 mg, 33% crystals yield based on the metal salt). *^{1}H NMR (600 MHz, d^6-DMSO) δ 8.70 (dd, J = 5.3, 1.7 Hz, 1H), 8.60 – 8.56 (m, 1H), 8.25 (d, J = 8.1 Hz, 1H), 8.20 (d, J = 8.3 Hz, 1H), 8.15 (td, J = 8.3, 7.8, 1.9 Hz, 1H), 7.71 (ddd, J = 8.2, 6.9, 1.1 Hz, 1H), 7.57 – 7.51 (m, 2H).* HR-MS (ESI positive mode) m/z calculated: 669.95, found: 669.944 for $[Ag_2(\textbf{L1})_2NO_3]^+$.

Compound 2 was prepared by dissolving AgOTf (129 mg, 0.5 mmol) in MeOH (5 ml) in one vial, and an equimolar amount of **L1** (98 mg, 0.5 mmol) was dissolved in MeOH (5 ml) in a different vial. The two solutions were combined by slow addition of the ligand solution to the metal solution and refluxed for 2 hours while being protected from light. The mixture was left to cool down to room temperature, then filtered. The solution was left to form crystals by slow evaporation (259 mg, 57% crystals yield based on the metal salt). *^{1}H NMR (600 MHz, d^6-DMSO) δ 8.70 (ddd, J = 4.9, 1.8, 0.9 Hz, 1H), 8.58 (dt, J = 8.3, 0.9 Hz, 1H), 8.25 (dt, J =*

8.3, 0.9 Hz, 1H), 8.20 (dd, J = 8.3, 1.0 Hz, 1H), 8.15 (ddd, J = 8.2, 7.4, 1.8 Hz, 1H), 7.71 (ddd, J = 8.2, 7.0, 1.0 Hz, 1H), 7.57 – 7.51 (m, 2H). HR-MS (ESI positive mode) m/z calculated: 756.91, found: 756.9095 for $[Ag_2(\mathbf{L1})_2OTf]^+$.

Compound 3 was prepared by dissolving $AgNO_3$ (85 mg, 0.5 mmol) in acetone (5 ml) in one vial, and an equimolar amount of **L2** (123 mg, 0.5 mmol) was dissolved in acetone (5 ml) in a different vial. The two solutions were combined by slow addition of the ligand solution to the metal solution and refluxed for 2 hours while being protected from light. The mixture was left to cool down to room temperature, then filtered. The solution was left to form crystals by slow evaporation (60 mg, 18% crystals yield based on the metal salt). *^1H NMR (600 MHz, d^6-DMSO) δ 8.96 (d, J = 8.3 Hz, 1H), 8.72 (d, J = 8.9 Hz, 1H), 8.47 (d, J = 8.8 Hz, 1H), 8.25 (d, J = 8.2 Hz, 1H), 8.22 (d, J = 8.7 Hz, 1H), 8.12 (d, J = 8.1 Hz, 1H), 7.90 (t, J = 7.3 Hz, 1H), 7.81 (t, J = 7.4 Hz, 1H), 7.69 (t, J = 7.7 Hz, 1H), 7.60 (t, J = 7.5 Hz, 1H).* HR-MS (ESI positive mode) m/z calculated: 599.09, found: 599.0884 for $[Ag(\mathbf{L2})_2NO_3]^+$.

Compound 4 was prepared by dissolving AgOTf (129 mg, 0.5 mmol) in acetone (5 ml) in one vial, and an equimolar amount of **L2** (123 mg, 0.5 mmol) was dissolved in acetone (5 ml) in a different vial. The two solutions were combined by slow addition of the ligand solution to the metal solution and refluxed for 2 hours while being protected from light. The mixture was left to cool down to room temperature, then filtered. The solution was left to form crystals by slow evaporation (66 mg, 13% crystals yield based on the metal salt). *^1H NMR (600 MHz, d^6-DMSO) δ 8.93 (dt, J = 8.3, 0.9 Hz, 1H), 8.69 (d, J = 8.9 Hz, 1H), 8.44 (d, J = 8.8 Hz, 1H), 8.23 (dt, J = 8.3, 0.9 Hz, 1H), 8.19 (dd, J = 8.4, 1.1 Hz, 1H), 8.10 (dd, J = 8.1, 1.4 Hz, 1H), 7.88 (ddd, J = 8.4, 6.9, 1.4 Hz, 1H), 7.79 (ddd, J = 8.2, 7.0, 1.0 Hz, 1H), 7.67 (ddd, J = 8.0, 6.9, 1.2 Hz, 1H), 7.59 (ddd, J = 8.1, 6.9, 1.0 Hz, 1H).* HR-MS (ESI positive mode) m/z calculated: 599.09, found: 599.0884 for $[Ag(\mathbf{L2})_2]^+$.

Compound 5 was prepared by dissolving AgOTf (129 mg, 0.5 mmol) in MeOH (5 ml) in one vial, and an equimolar amount of **L3** (99 mg, 0.5 mmol) was dissolved in MeOH (5 ml) in a different vial. The two solutions were combined by slow addition of the ligand solution to the metal solution and refluxed for 2 hours while being protected from light. The mixture was left to cool down to room temperature, then filtered. The solution was left to form crystals by slow evaporation (205 mg, 34% crystals yield based on the metal salt). *^1H NMR (600 MHz, d^6-DMSO) δ 9.07 (d, J = 4.8 Hz, 2H), 8.54 (d, J = 8.4 Hz, 1H), 8.22 (d, J = 8.3 Hz, 1H), 7.75 (dd, J = 8.3, 7.0 Hz, 1H), 7.66 (t, J = 4.8 Hz, 1H), 7.57 (dd, J = 8.3, 7.0 Hz, 1H).* HR-MS (ESI positive mode) m/z calculated: 758.90, found: 758.9020 for $[Ag_2(\mathbf{L3})_2OTf]^+$.

8. Copies of all characterisation data

Ligand L1

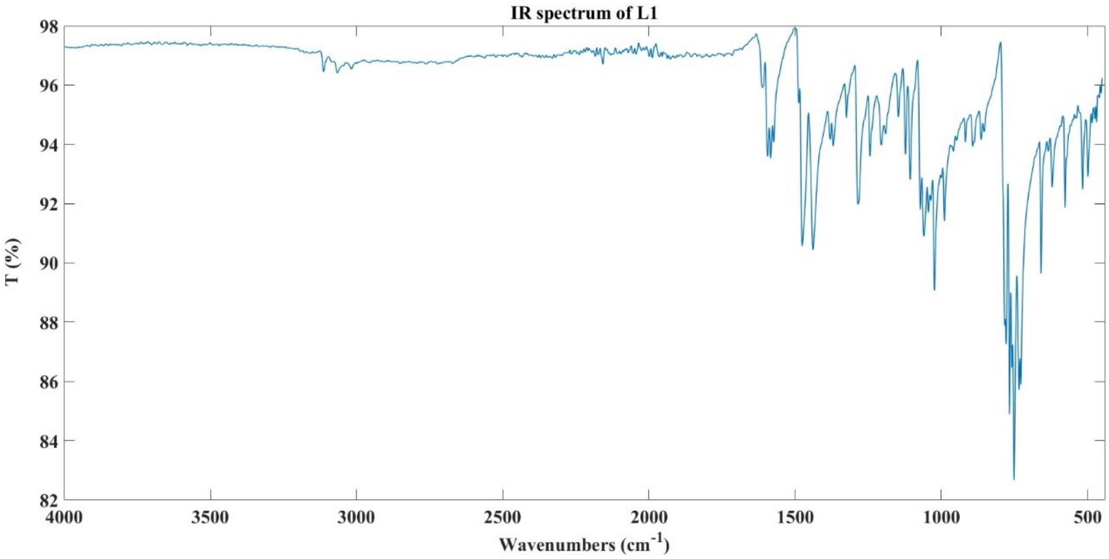

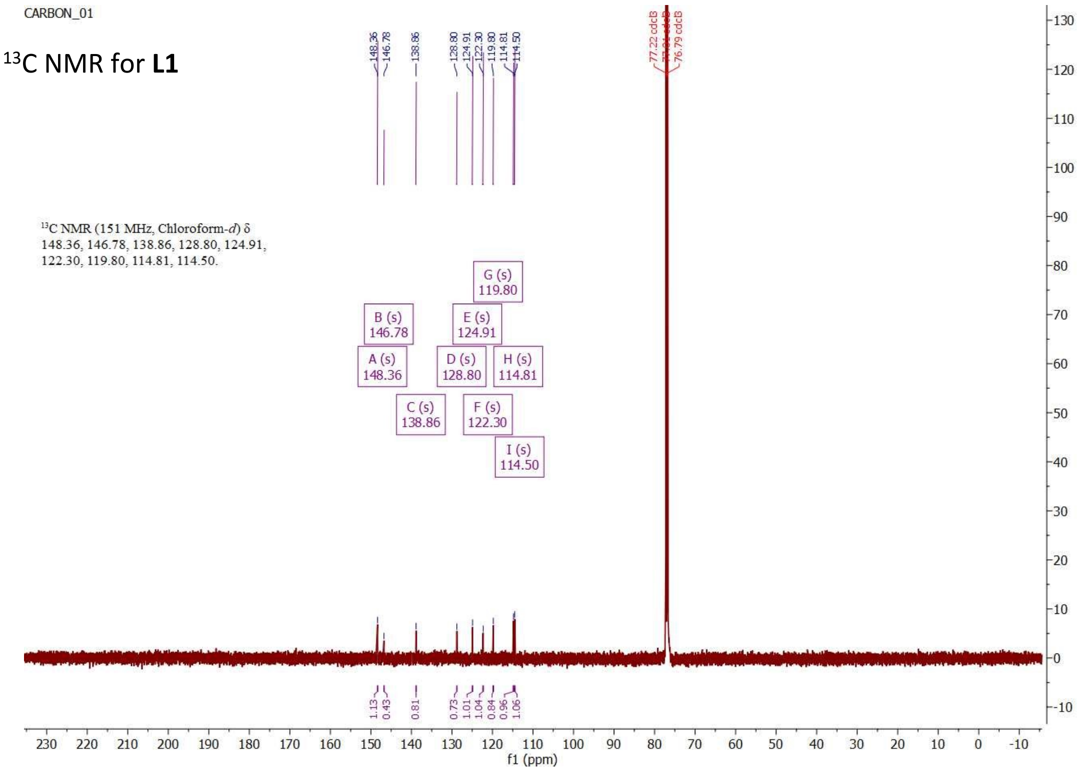

^{1}H NMR for **L1**

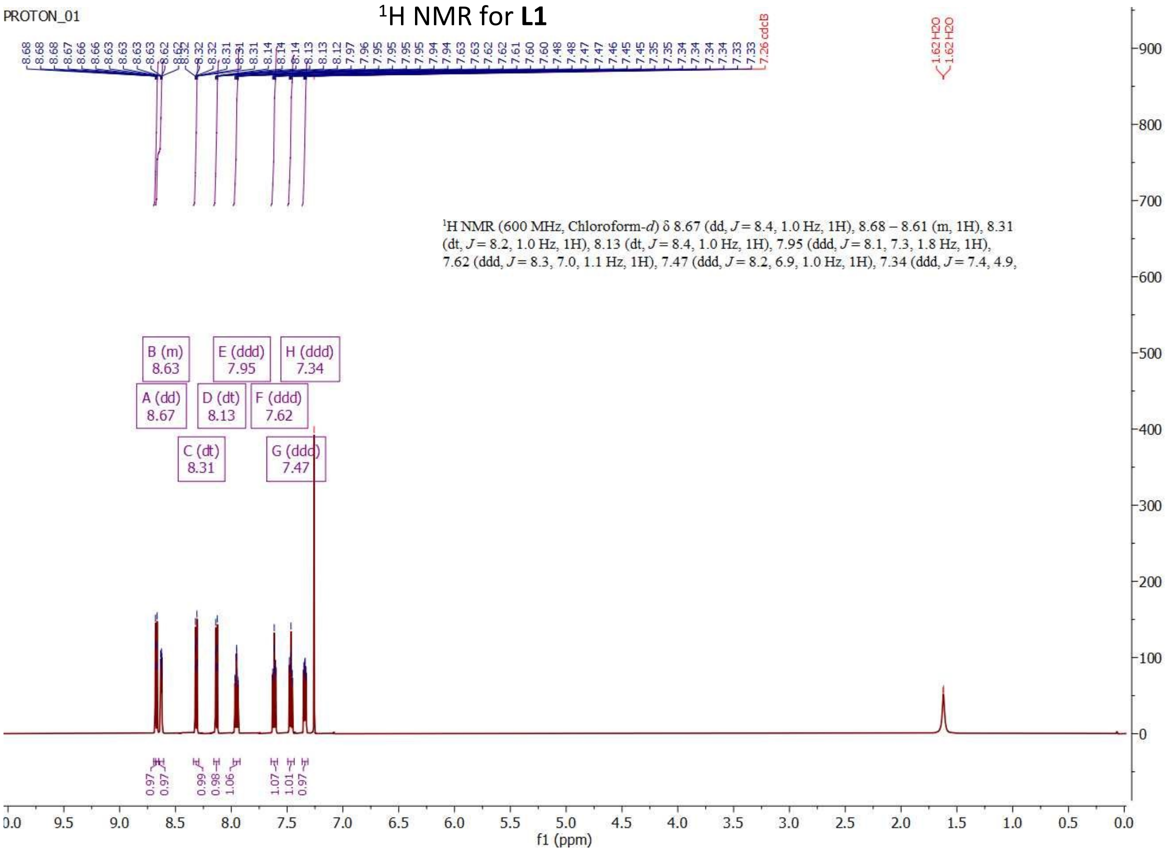

Mass spectrum for **L1**

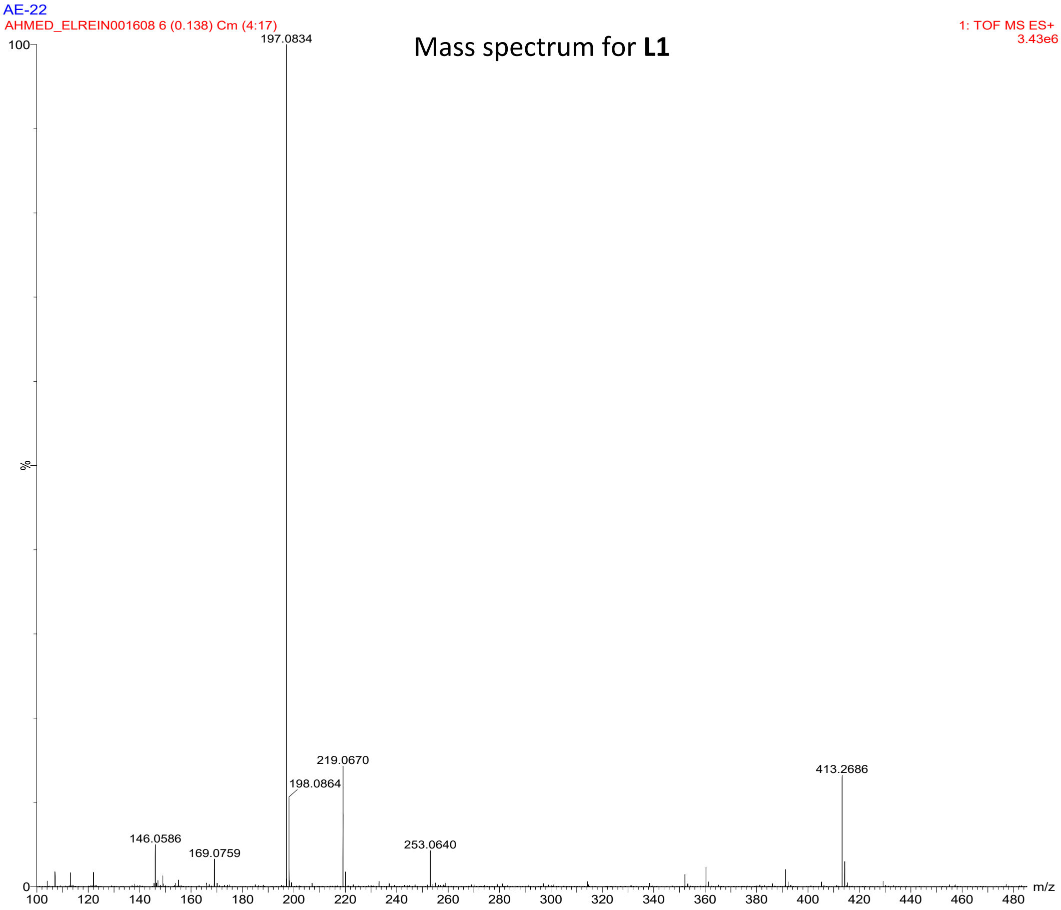

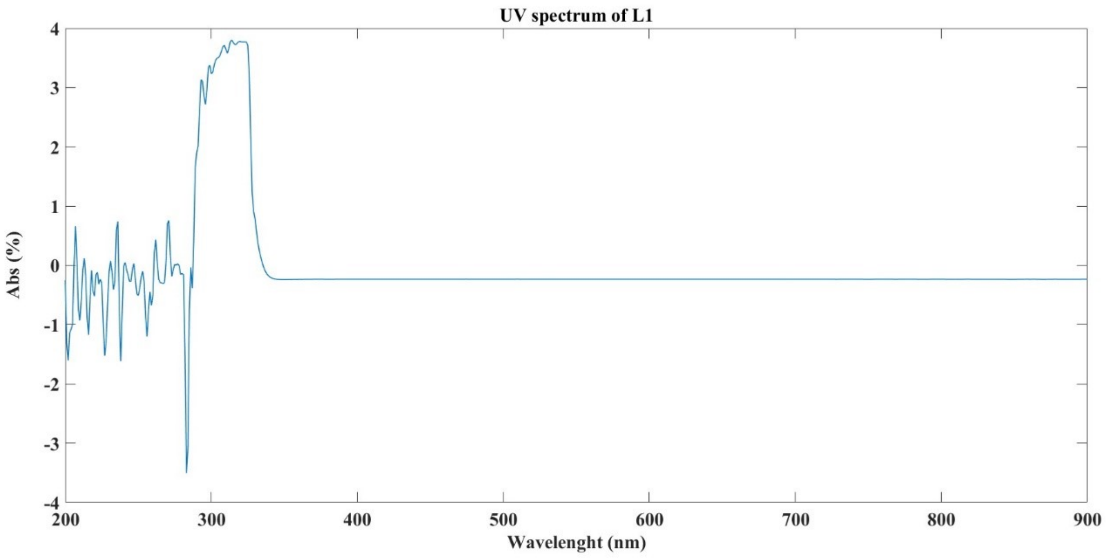

Figure S8**. Characterisation data for **L1.

Ligand L2

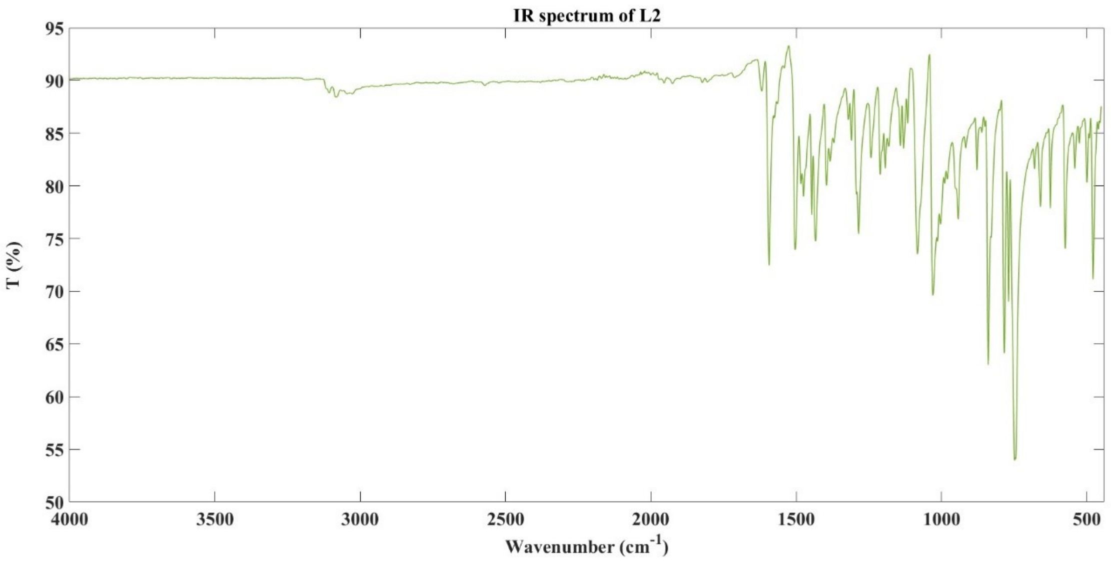

IR spectrum of L2
T (%)
Wavenumber (cm^{-1})

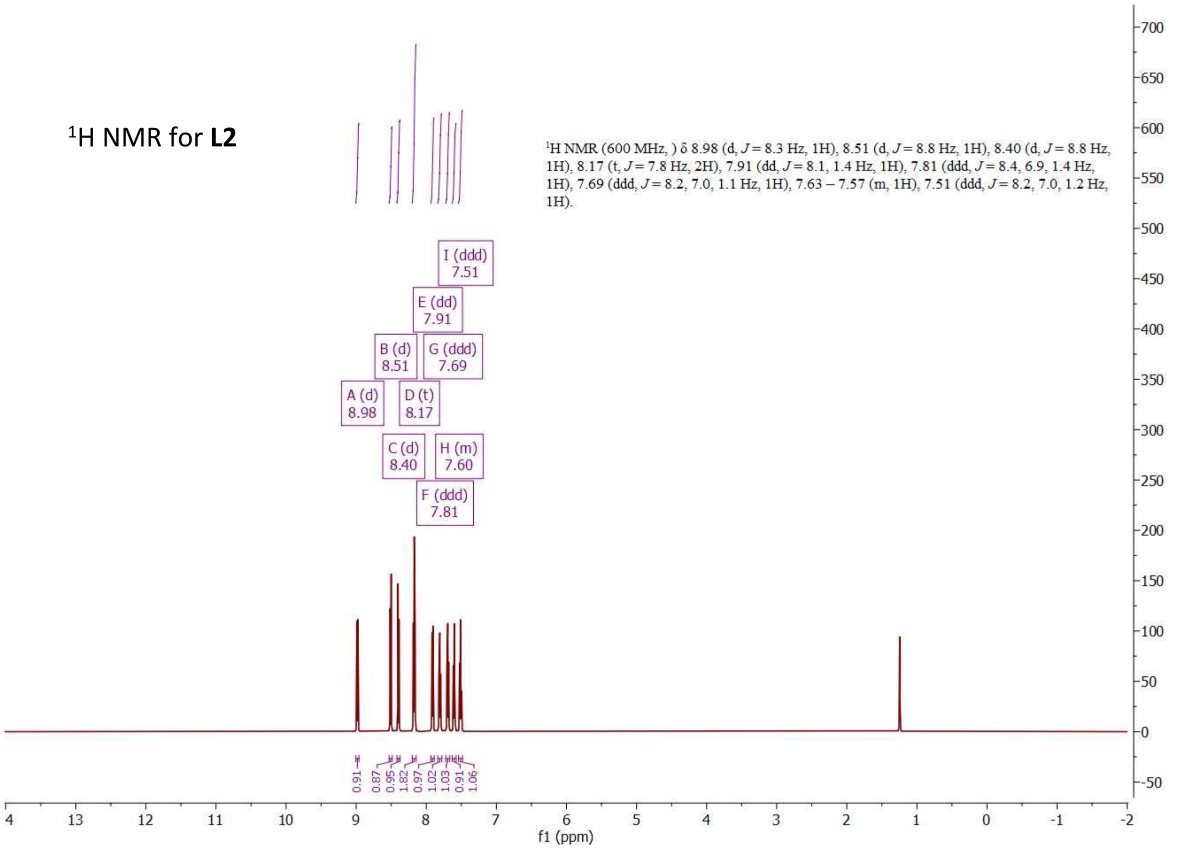

^{1}H NMR for **L2**
^{1}H NMR (600 MHz,) δ 8.98 (d, J = 8.3 Hz, 1H), 8.51 (d, J = 8.8 Hz, 1H), 8.40 (d, J = 8.8 Hz, 1H), 8.17 (t, J = 7.8 Hz, 2H), 7.91 (dd, J = 8.1, 1.4 Hz, 1H), 7.81 (ddd, J = 8.4, 6.9, 1.4 Hz, 1H), 7.69 (ddd, J = 8.2, 7.0, 1.1 Hz, 1H), 7.63 – 7.57 (m, 1H), 7.51 (ddd, J = 8.2, 7.0, 1.2 Hz, 1H).
I (ddd) 7.51
E (dd) 7.91
B (d) 8.51
G (ddd) 7.69
A (d) 8.98
D (t) 8.17
C (d) 8.40
H (m) 7.60
F (ddd) 7.81
f1 (ppm)

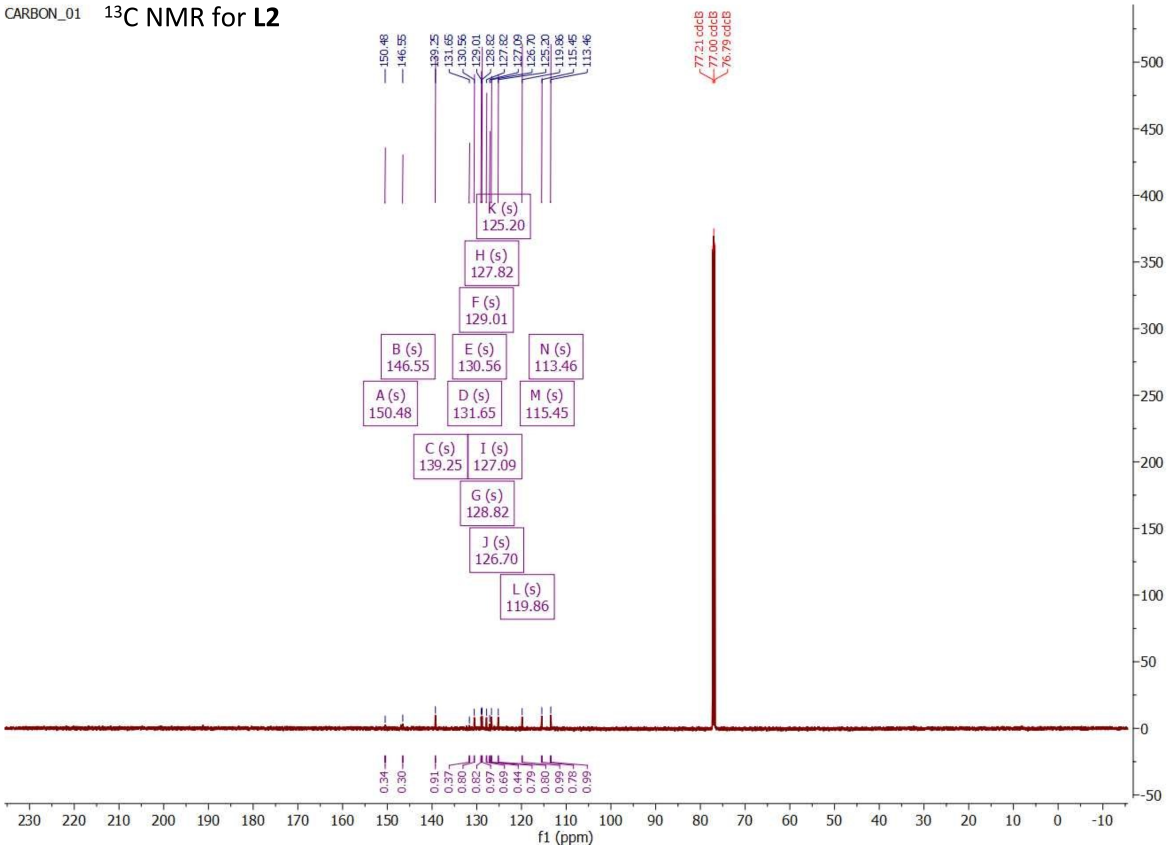
CARBON_01
^{13}C NMR for **L2**
150.48
146.55
139.25
131.65
130.56
129.01
128.82
127.82
127.09
126.70
125.20
119.86
115.45
113.46
77.21 cdcl3
77.00 cdcl3
76.79 cdcl3
A (s) 150.48
B (s) 146.55
C (s) 139.25
D (s) 131.65
E (s) 130.56
F (s) 129.01
G (s) 128.82
H (s) 127.82
I (s) 127.09
J (s) 126.70
K (s) 125.20
L (s) 119.86
M (s) 115.45
N (s) 113.46
f1 (ppm)

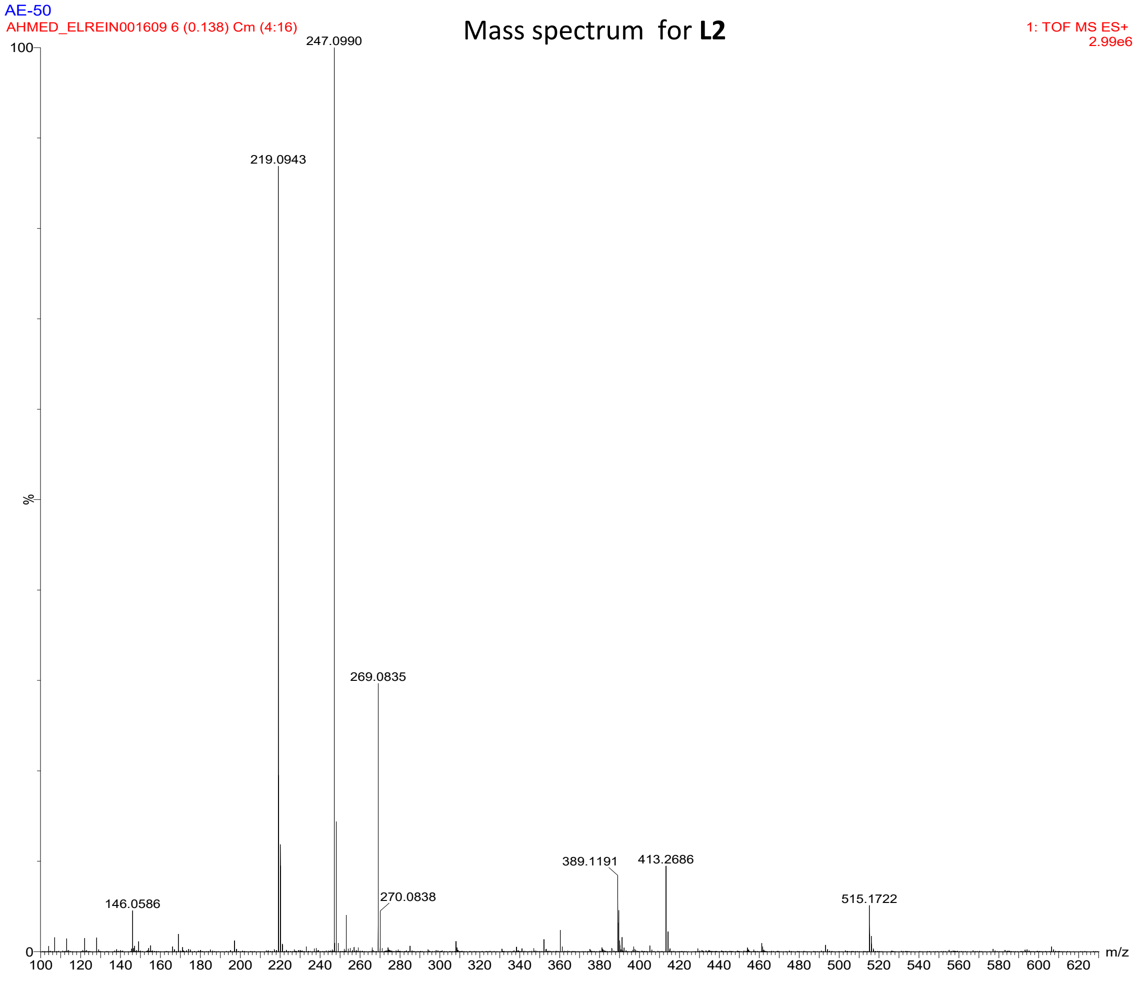
AE-50
AHMED_ELREIN001609 6 (0.138) Cm (4:16)
Mass spectrum for **L2**
1: TOF MS ES+
2.99e6
247.0990
219.0943
269.0835
270.0838
146.0586
389.1191
413.2686
515.1722
%
m/z

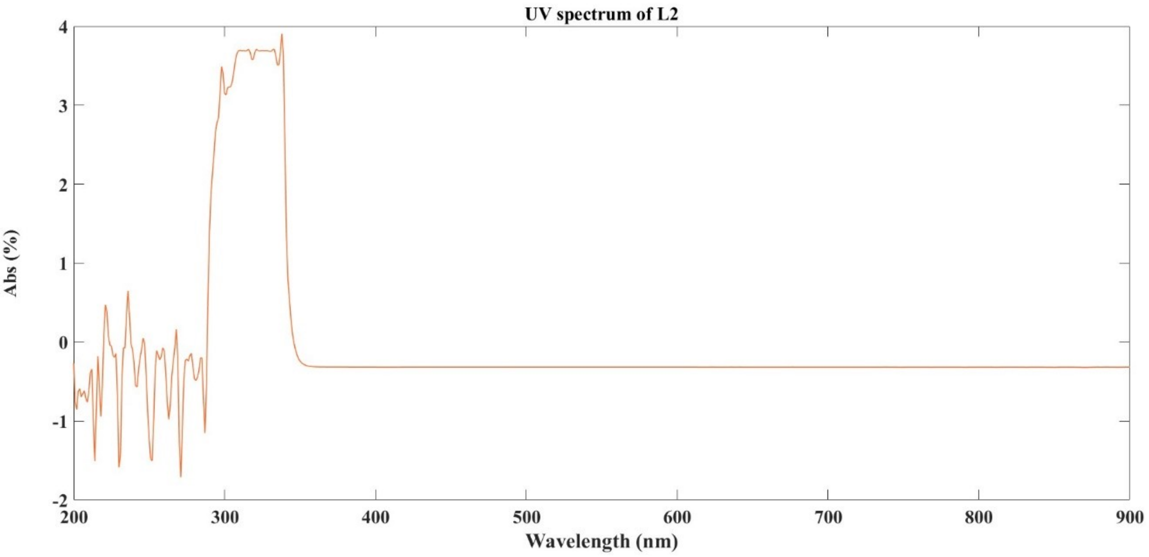

Figure S9. *Characterisation data for* ***L2.***

Ligand L3

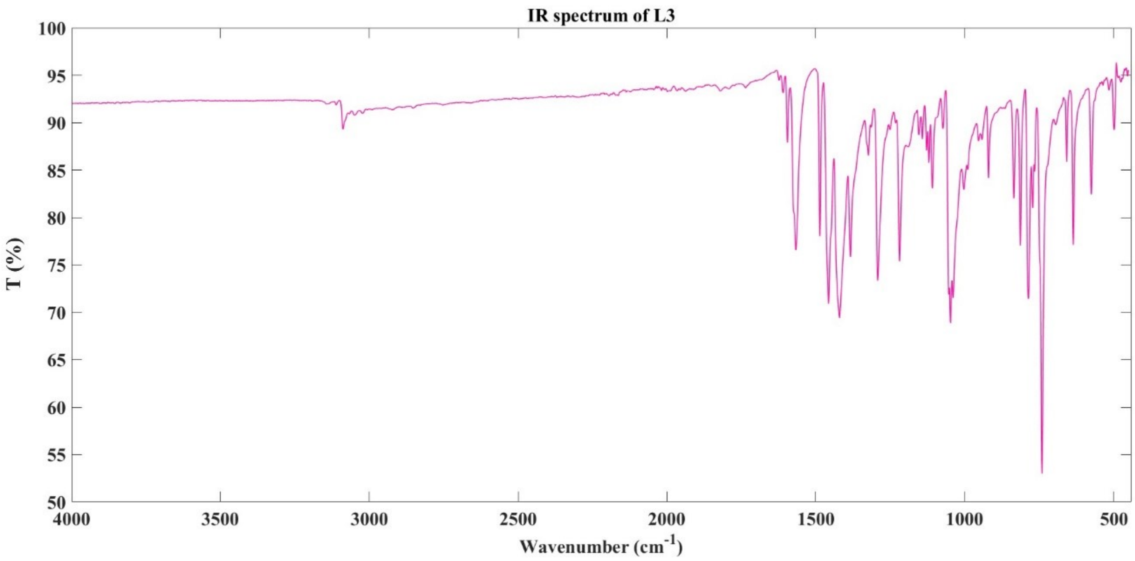

^{13}C NMR for **L3**

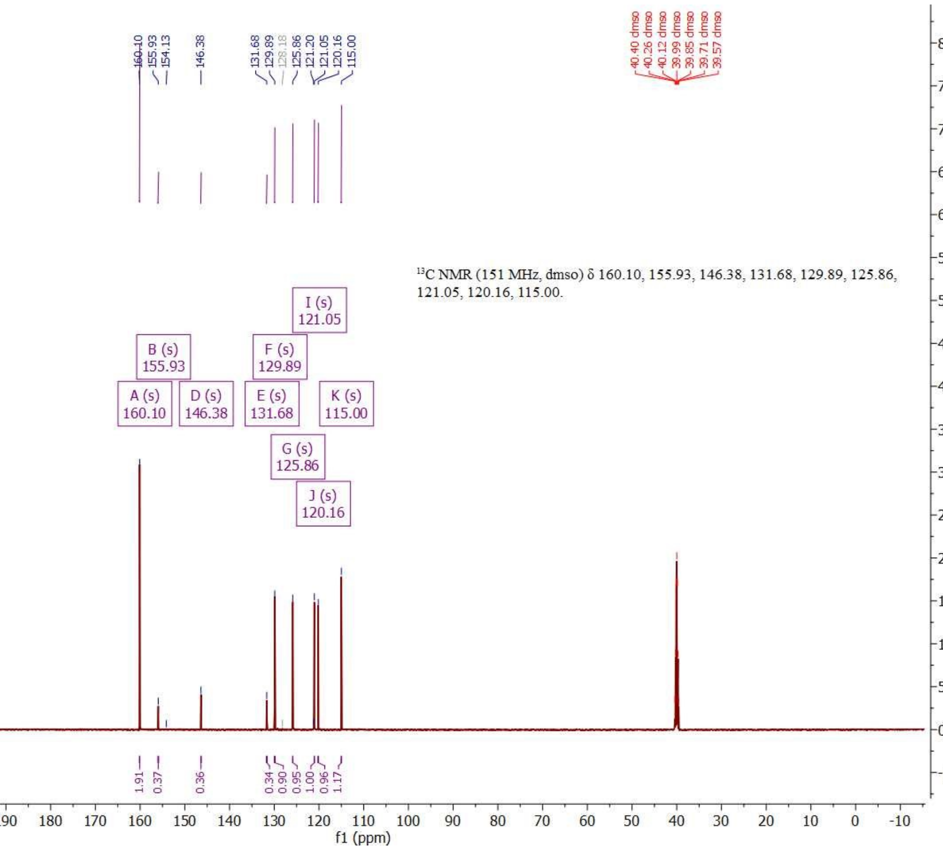

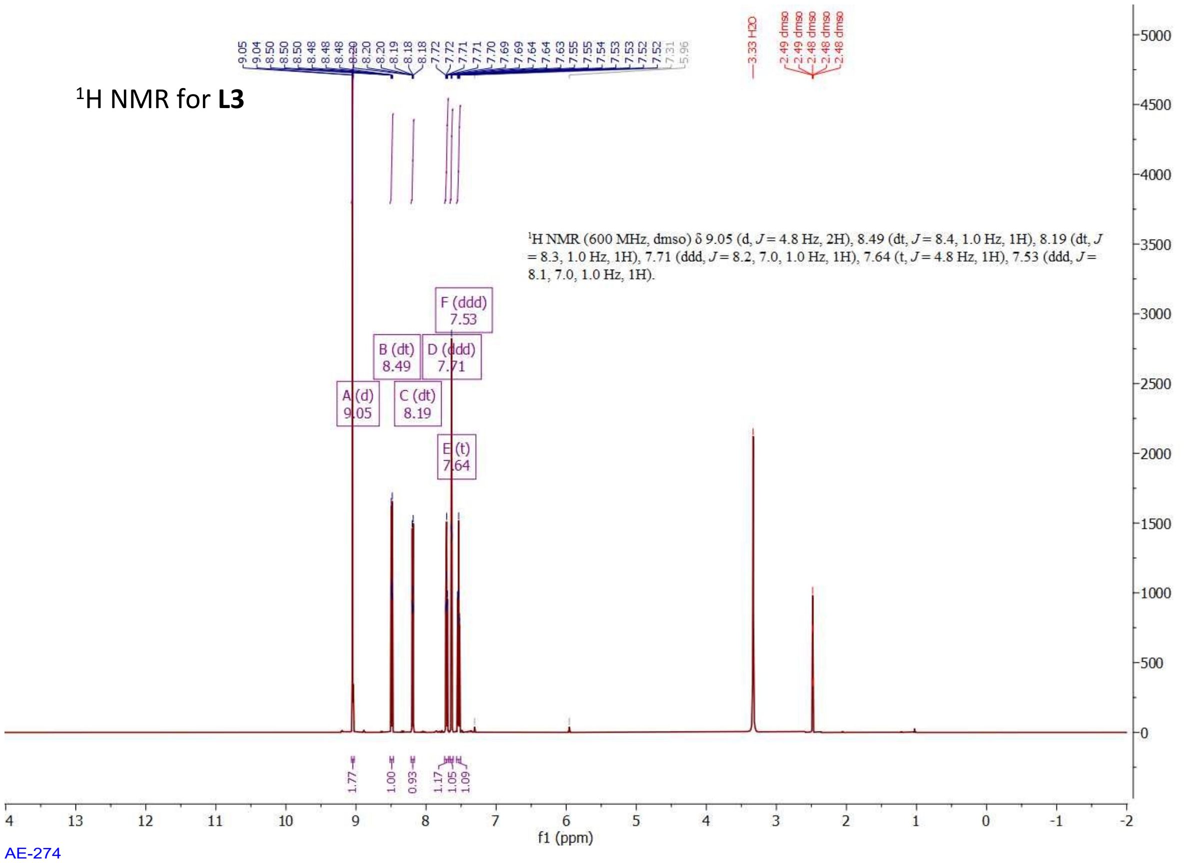

^{1}H NMR for **L3**
^{1}H NMR (600 MHz, dmso) δ 9.05 (d, J = 4.8 Hz, 2H), 8.49 (dt, J = 8.4, 1.0 Hz, 1H), 8.19 (dt, J = 8.3, 1.0 Hz, 1H), 7.71 (ddd, J = 8.2, 7.0, 1.0 Hz, 1H), 7.64 (t, J = 4.8 Hz, 1H), 7.53 (ddd, J = 8.1, 7.0, 1.0 Hz, 1H).
A (d) 9.05
B (dt) 8.49
C (dt) 8.19
D (ddd) 7.71
E (t) 7.64
F (ddd) 7.53
3.33 H2O
2.49 dmso
1.77
1.00
0.93
1.17
1.05
1.09
f1 (ppm)

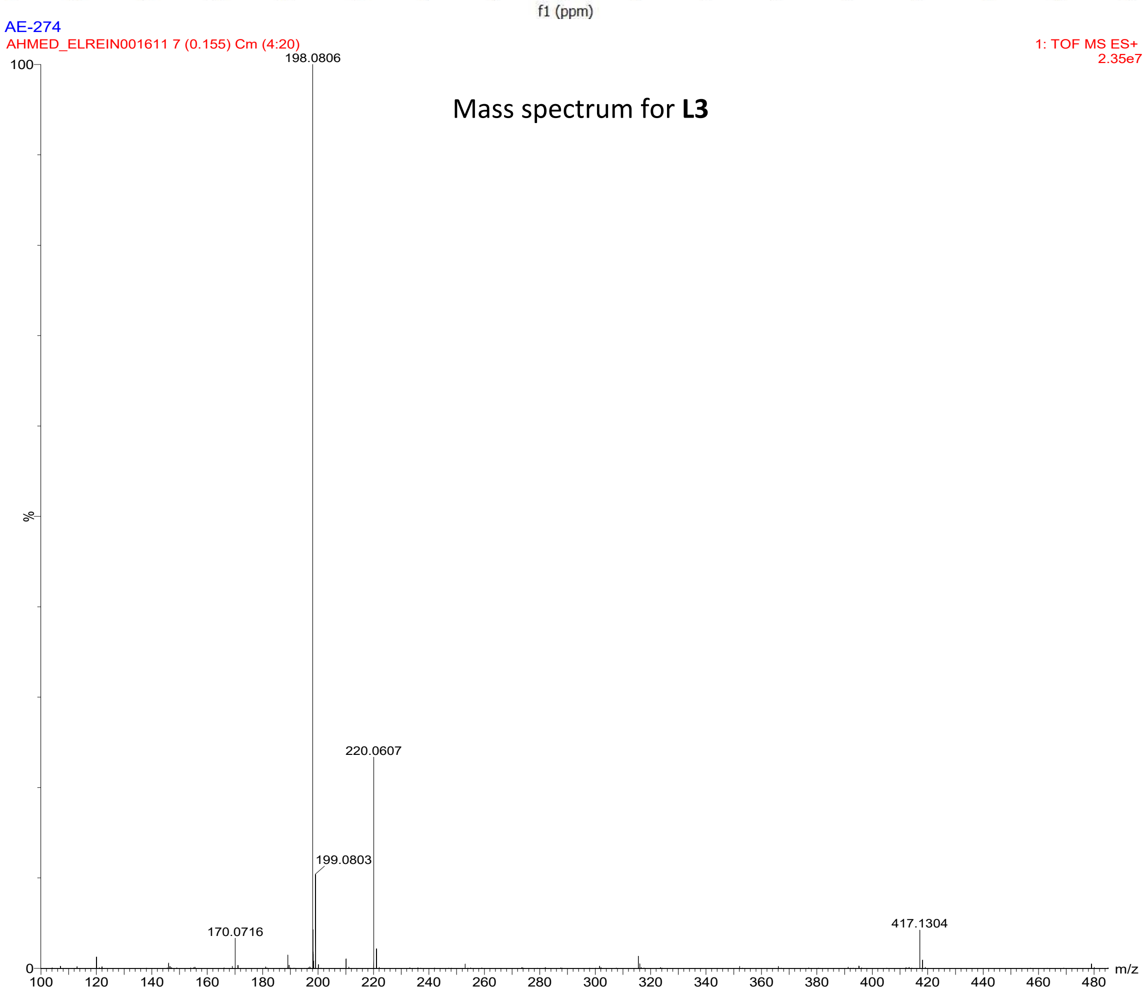

AE-274
AHMED_ELREIN001611 7 (0.155) Cm (4:20)
1: TOF MS ES+
2.35e7
Mass spectrum for **L3**
198.0806
220.0607
199.0803
170.0716
417.1304
%
m/z

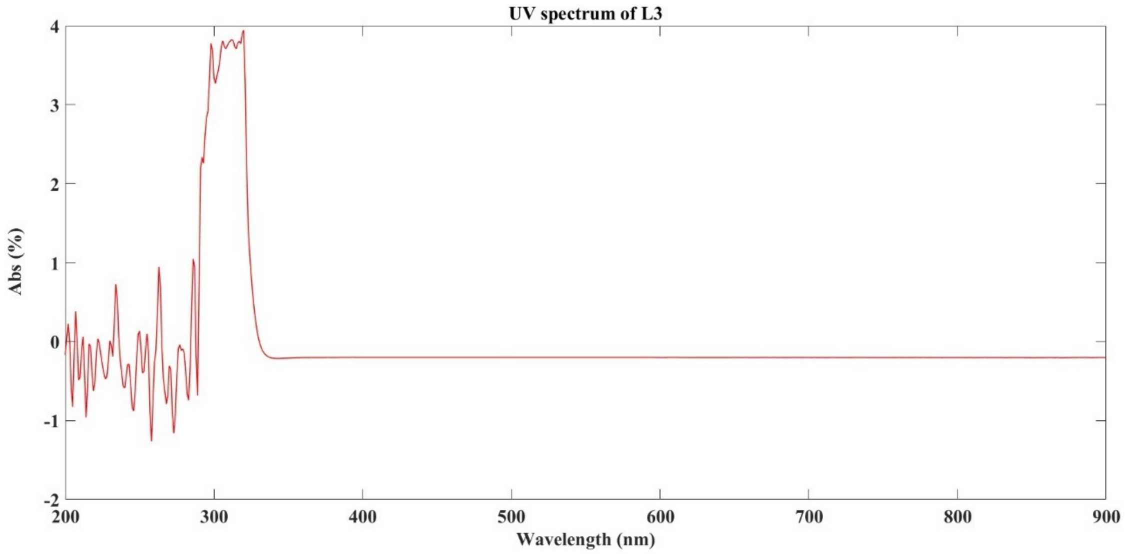

Figure S10. *Characterisation data for **L3**.*

*Compound **1***

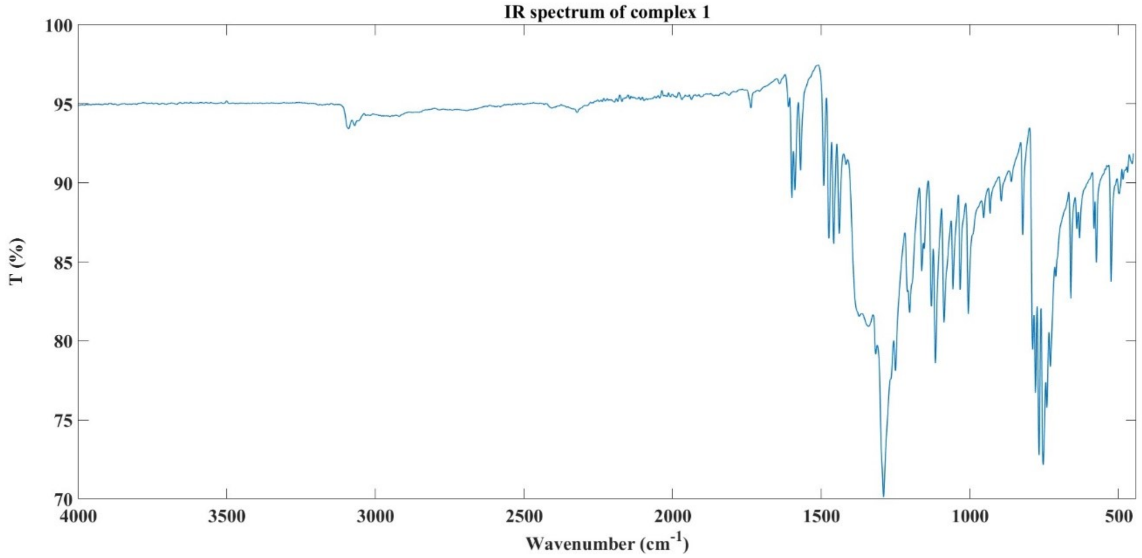

¹H NMR for **C1**

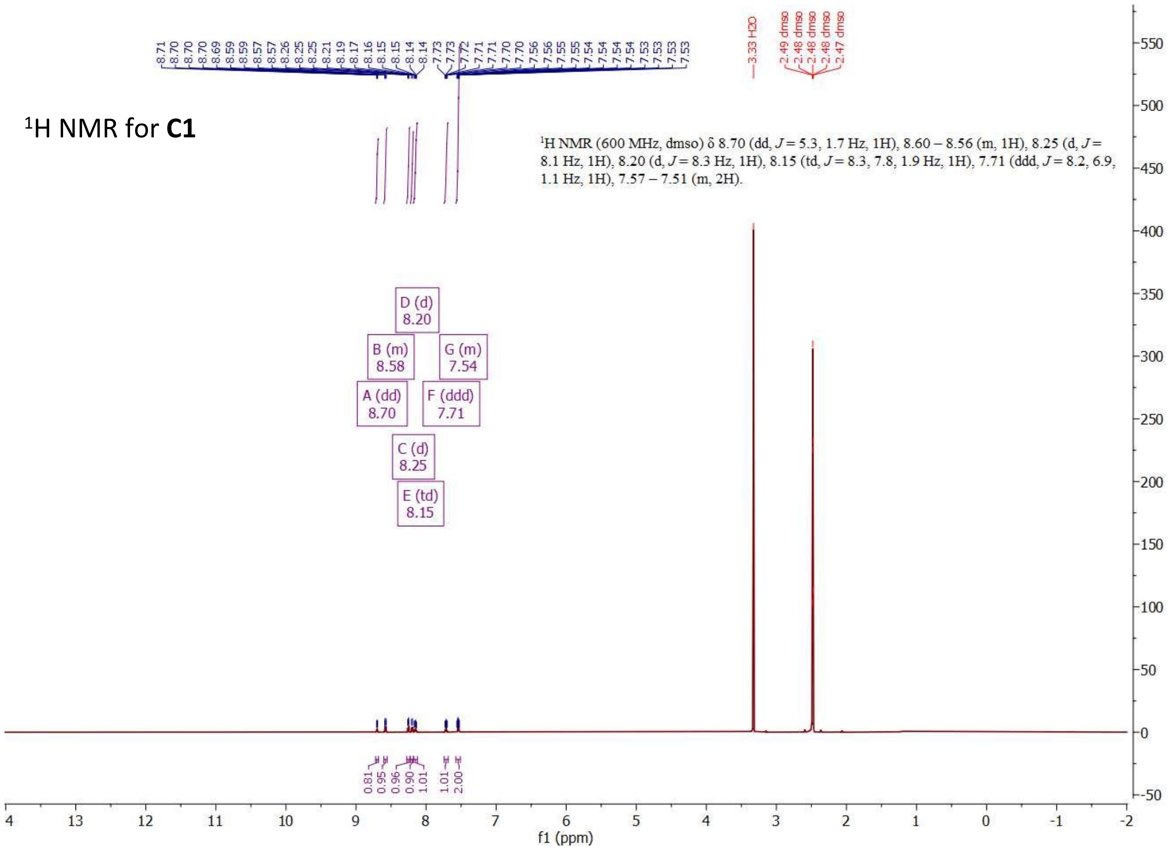

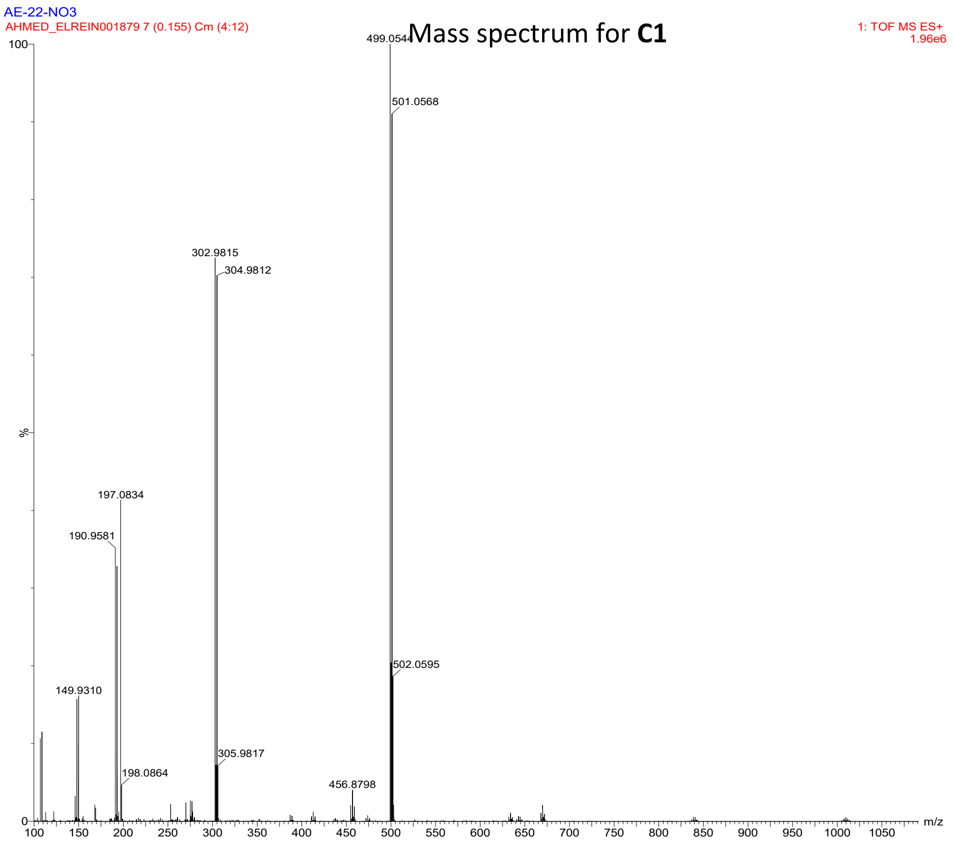
AE-22-NO3
AHMED_ELREIN001879 7 (0.155) Cm (4:12)
Mass spectrum for C1
1: TOF MS ES+
1.96e6
499.0544
501.0568
502.0595
302.9815
304.9812
305.9817
197.0834
190.9581
198.0864
149.9310
456.8798
%
m/z

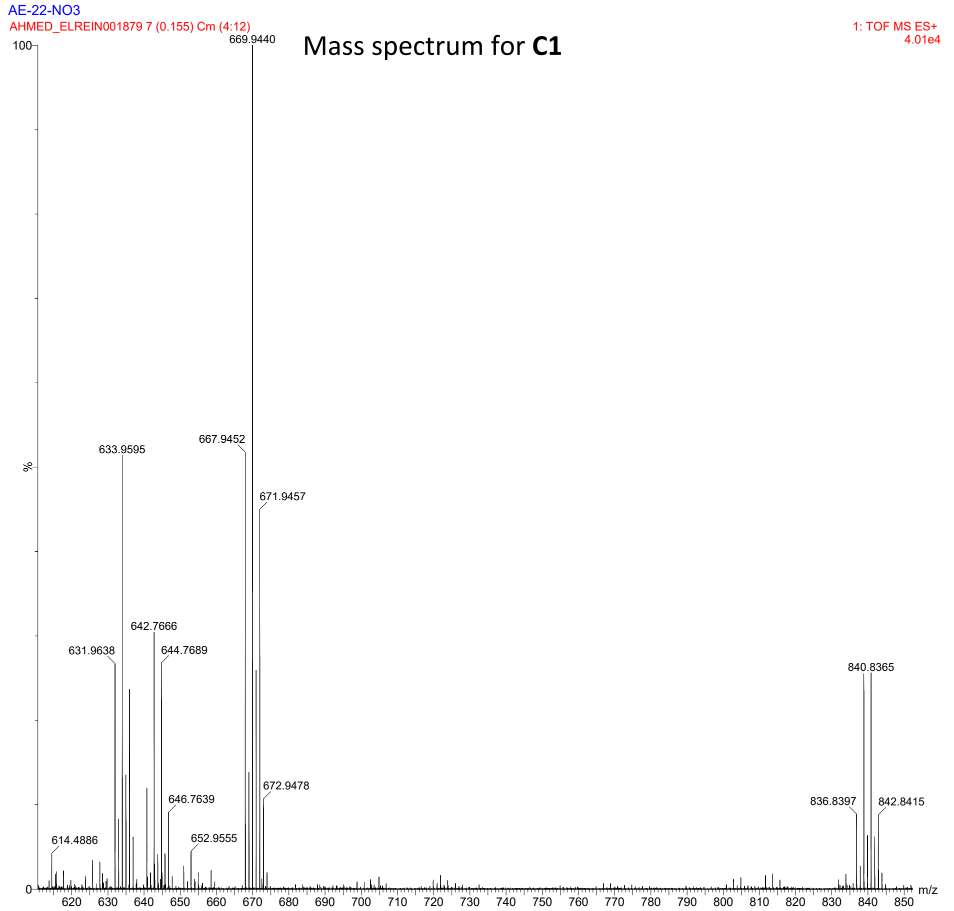
AE-22-NO3
AHMED_ELREIN001879 7 (0.155) Cm (4:12)
Mass spectrum for C1
1: TOF MS ES+
4.01e4
669.9440
667.9452
671.9457
672.9478
633.9595
631.9638
642.7666
644.7689
646.7639
652.9555
614.4886
840.8365
836.8397
842.8415
%
m/z

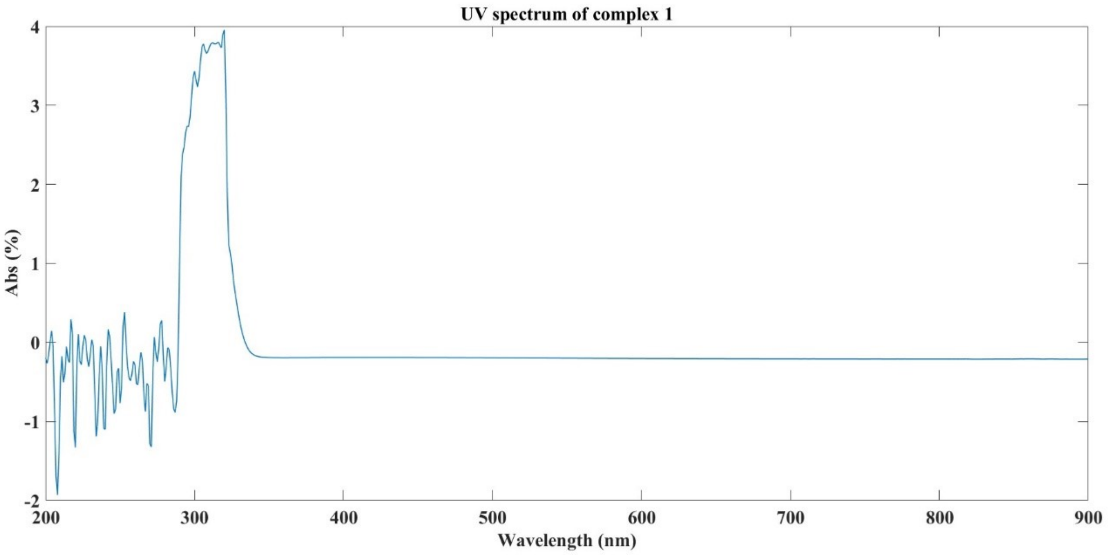

Figure S11. *Characterisation data for complex **1**.*

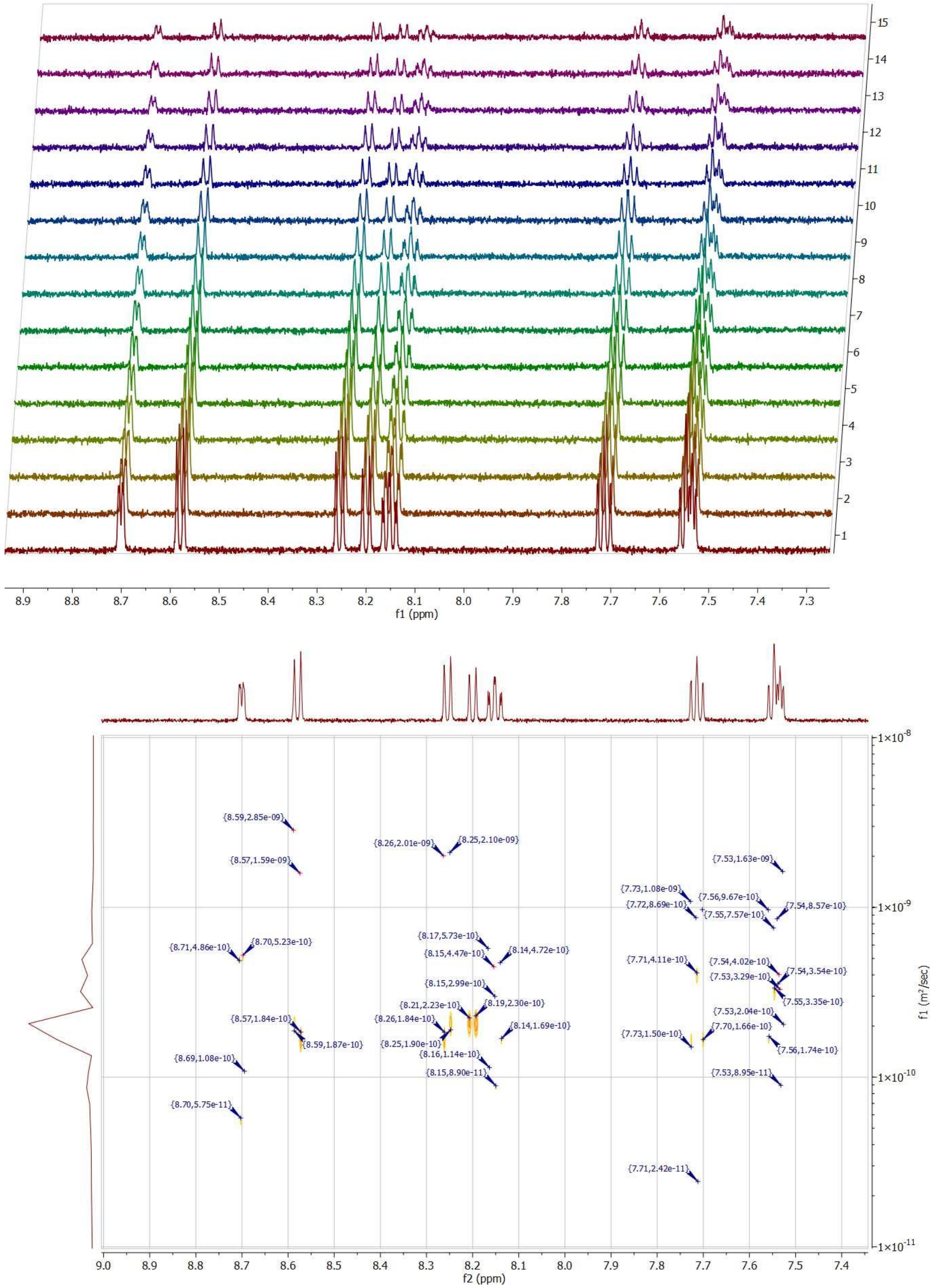

Figure S12. *Diffusion* ^{1}*H NNMR studies for complex* ***1****.*

*Compound **2***

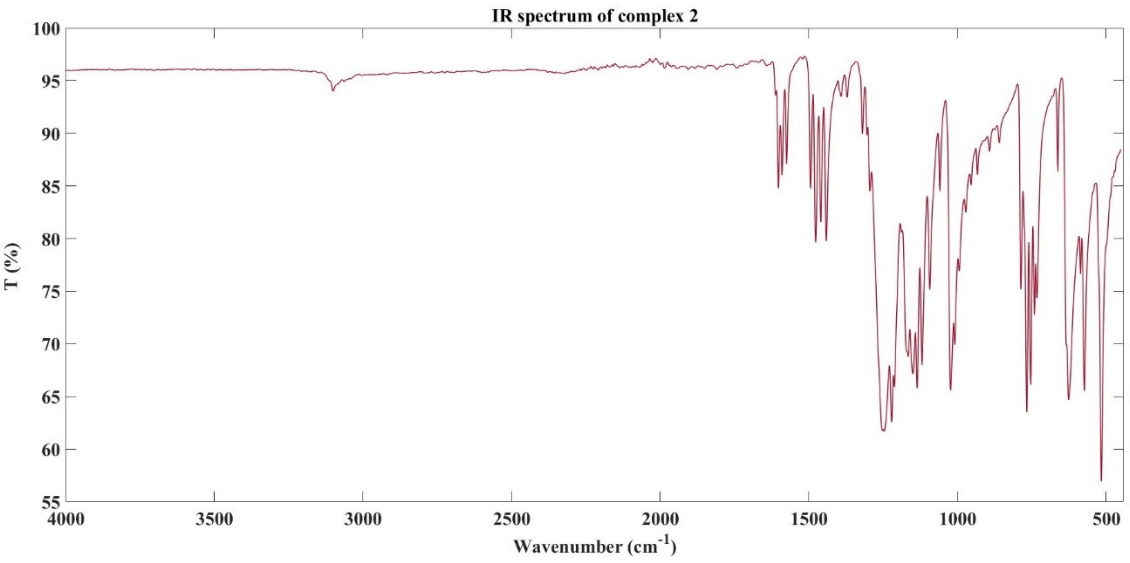

^{1}H NMR for **C2**

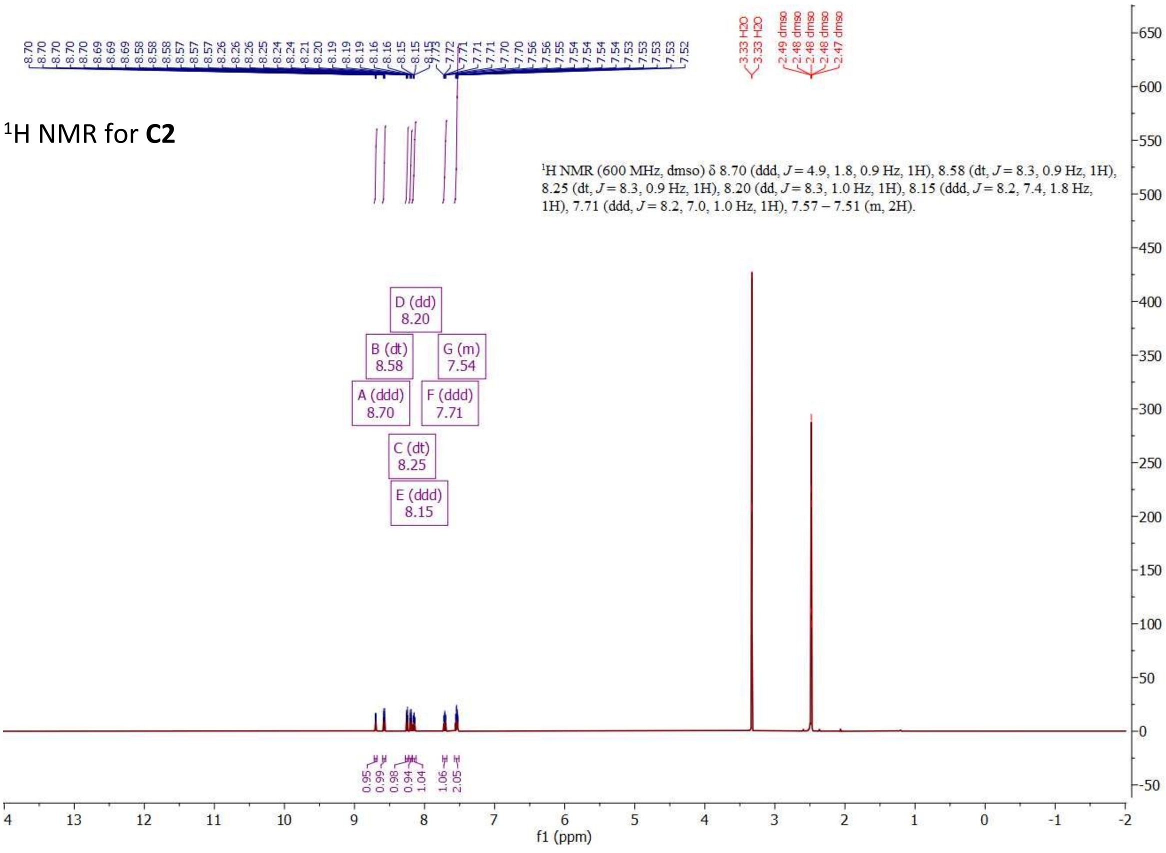

^{19}F NMR for **C2**

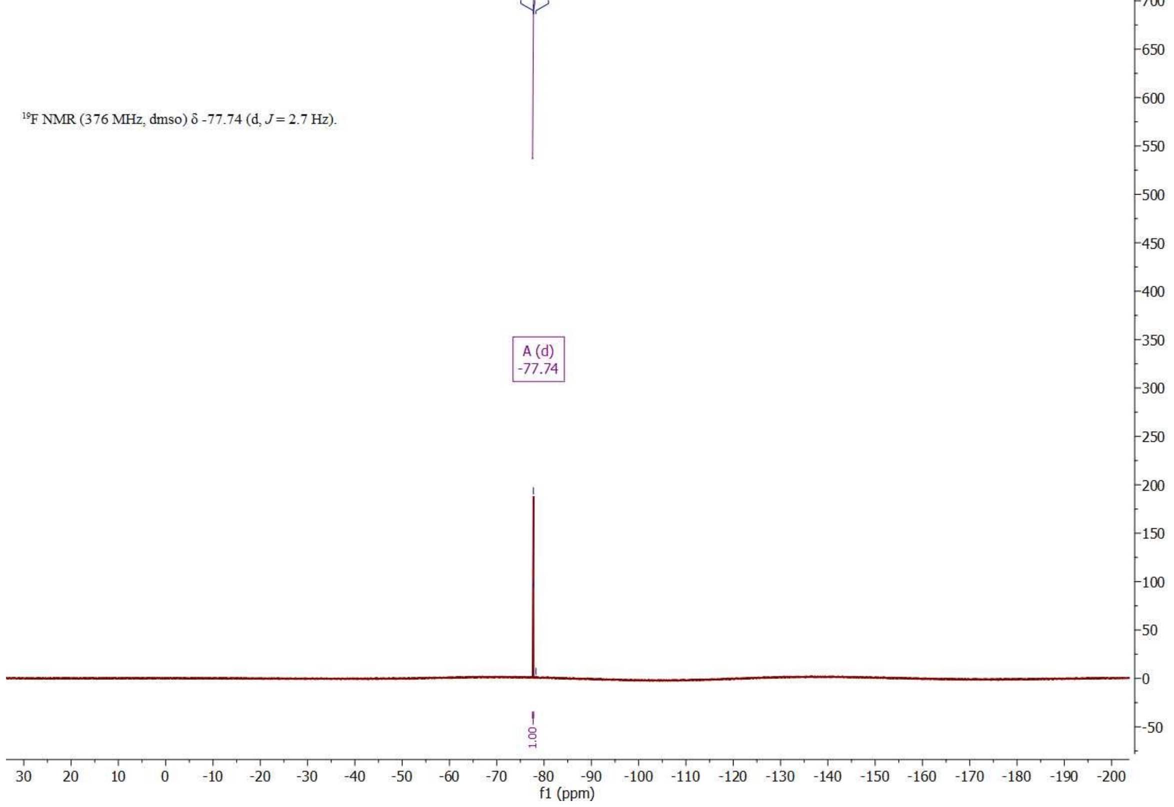

Mass spectrum for **C2**

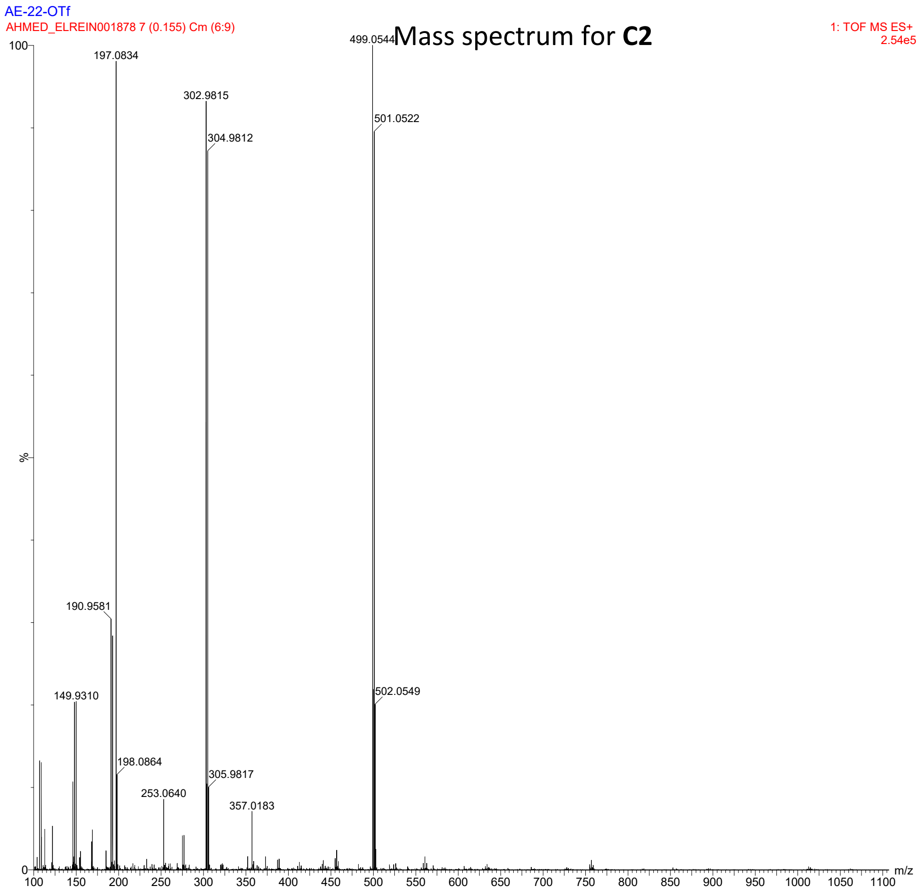

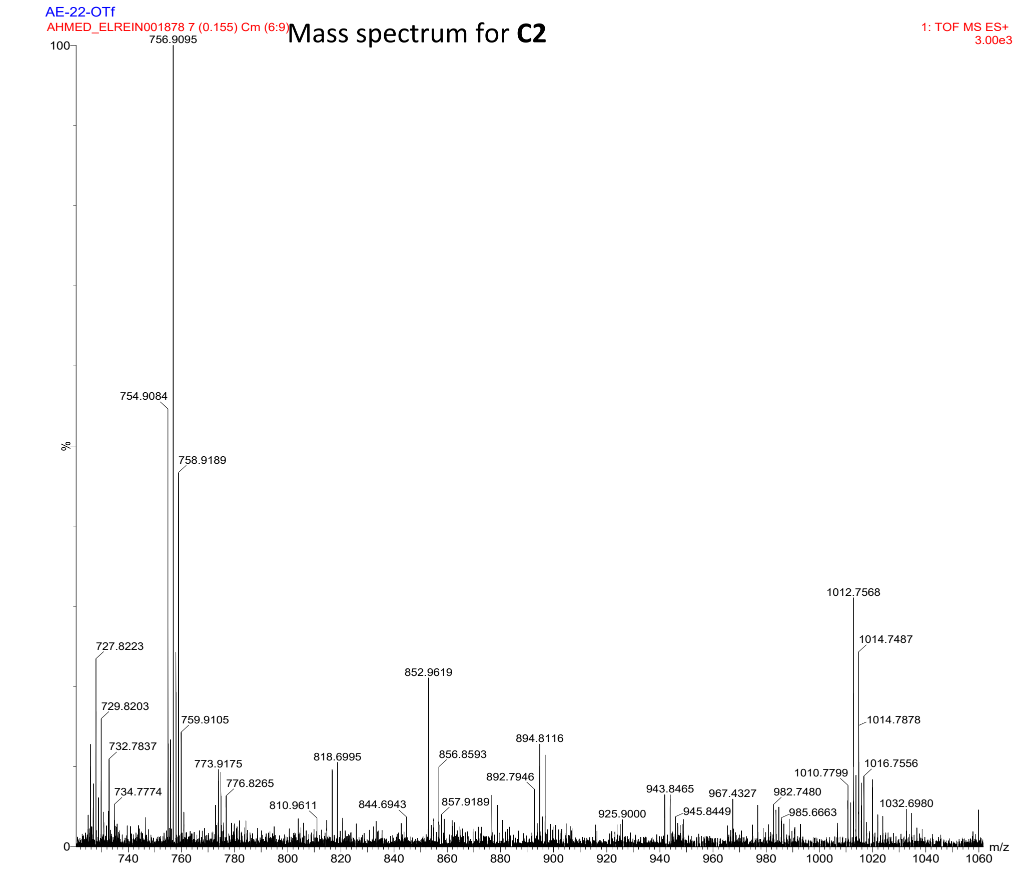

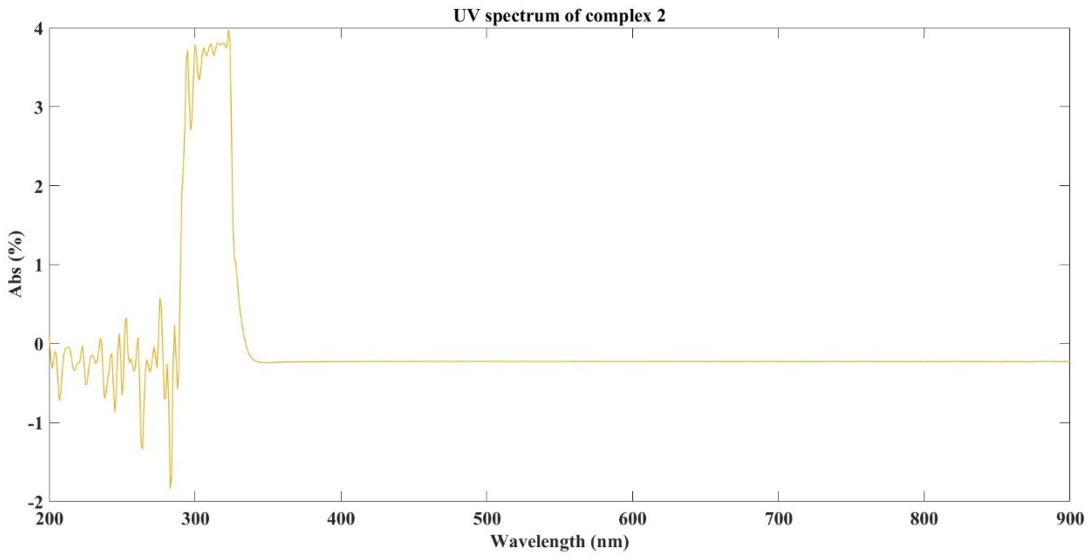

Figure S13. *Characterisation data for complex* ***2****.*

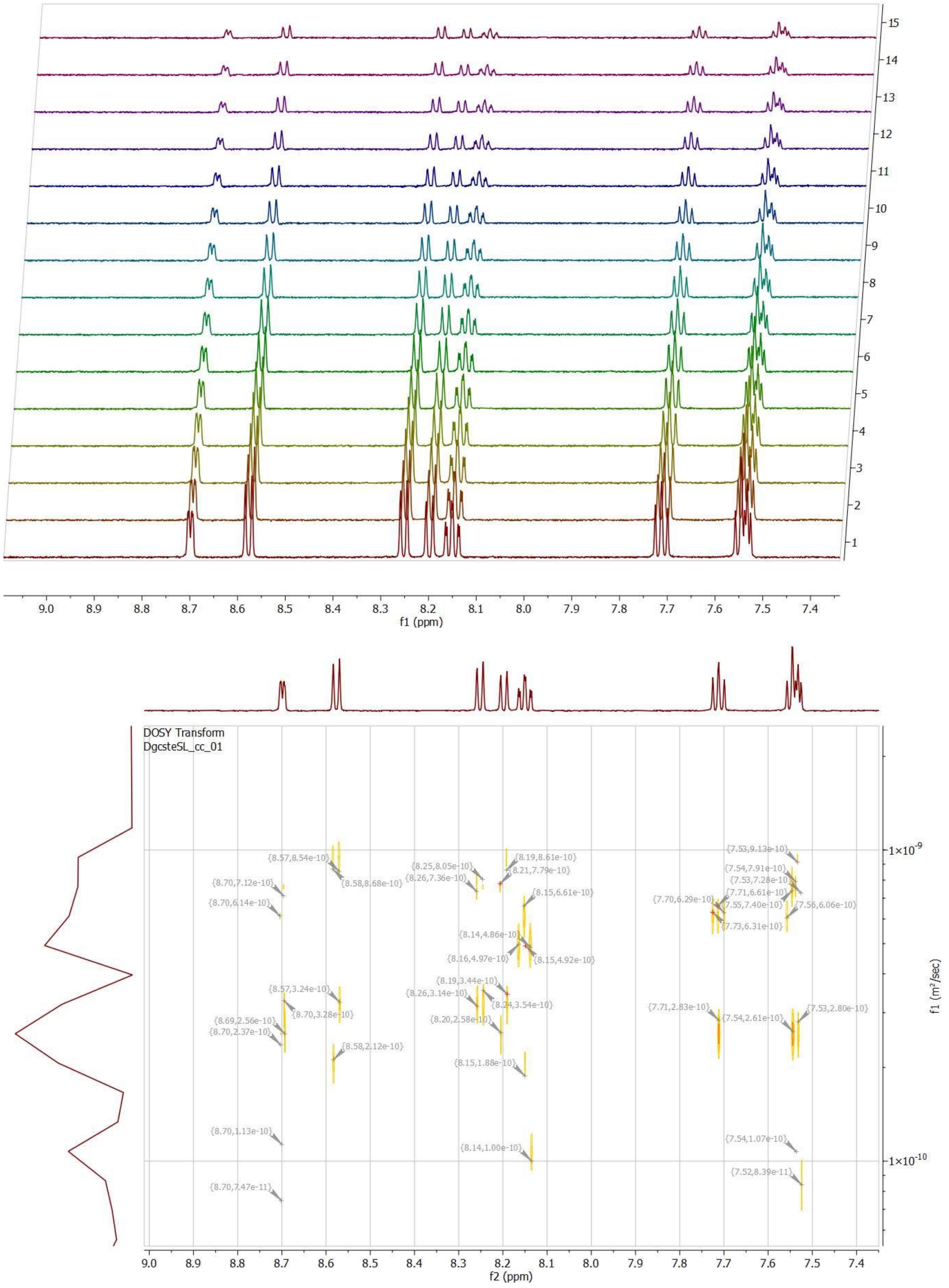

Figure S14. *Diffusion ^{1}H NNMR studies for complex* ***2****.*

Compound 1 vs Compound 2 vs L1

- ***UV spectrum***

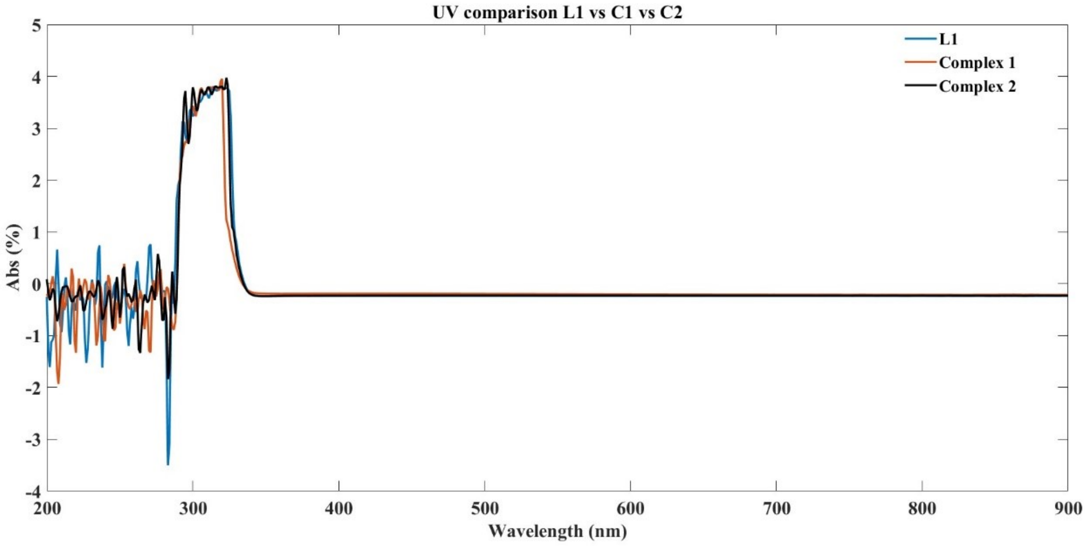

Figure S15. *UV studies comparison between Ag(**L1**) based complexes at 0.012mM.*

- ***^{1}H NMR in d^{6}-DMSO***

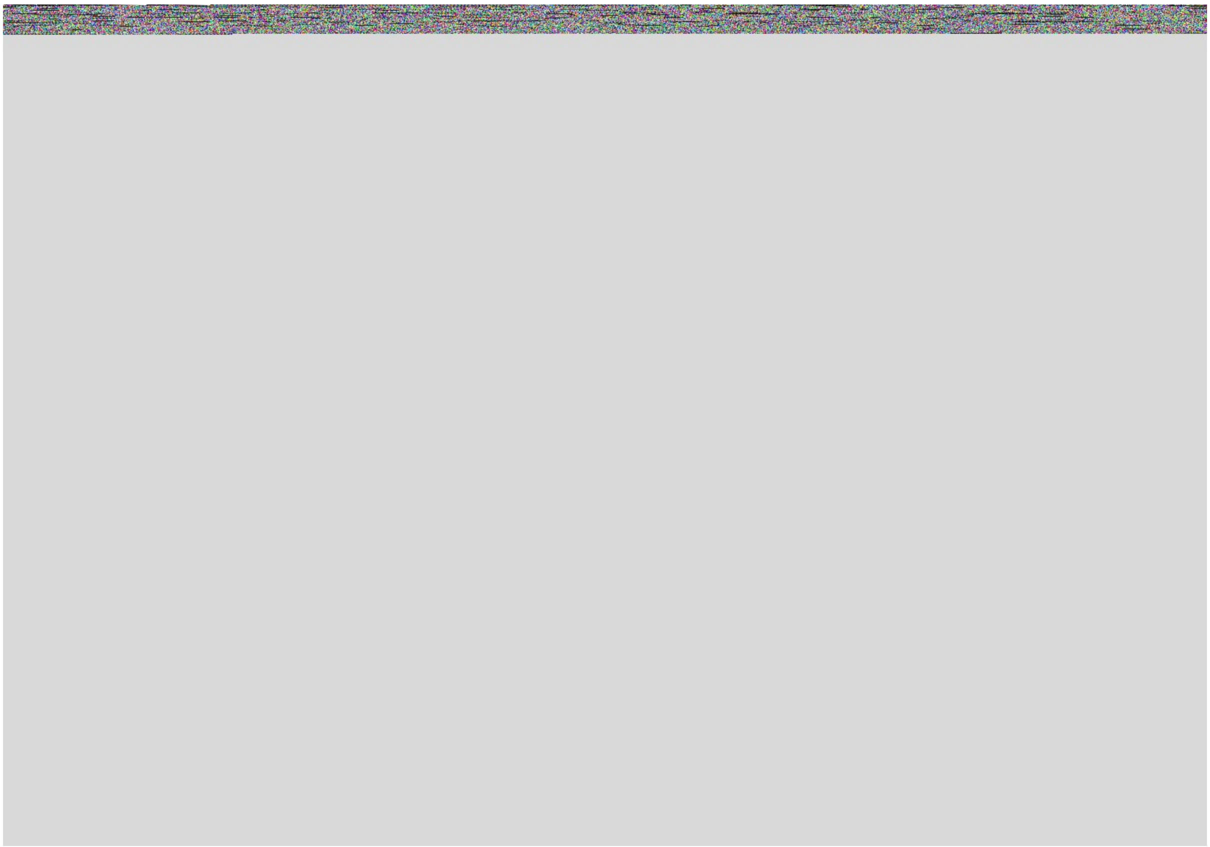

Figure S16. *^{1}H NMR comparison between Ag(**L1**) based complexes and anion effect.*

*Compound **3***

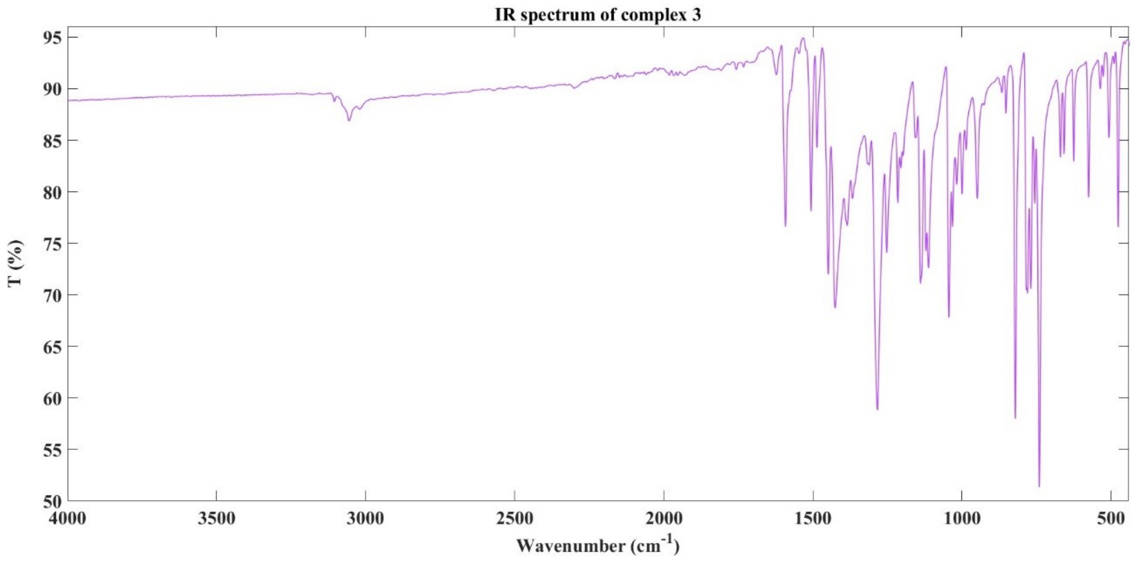

^{1}H NMR for **C3**

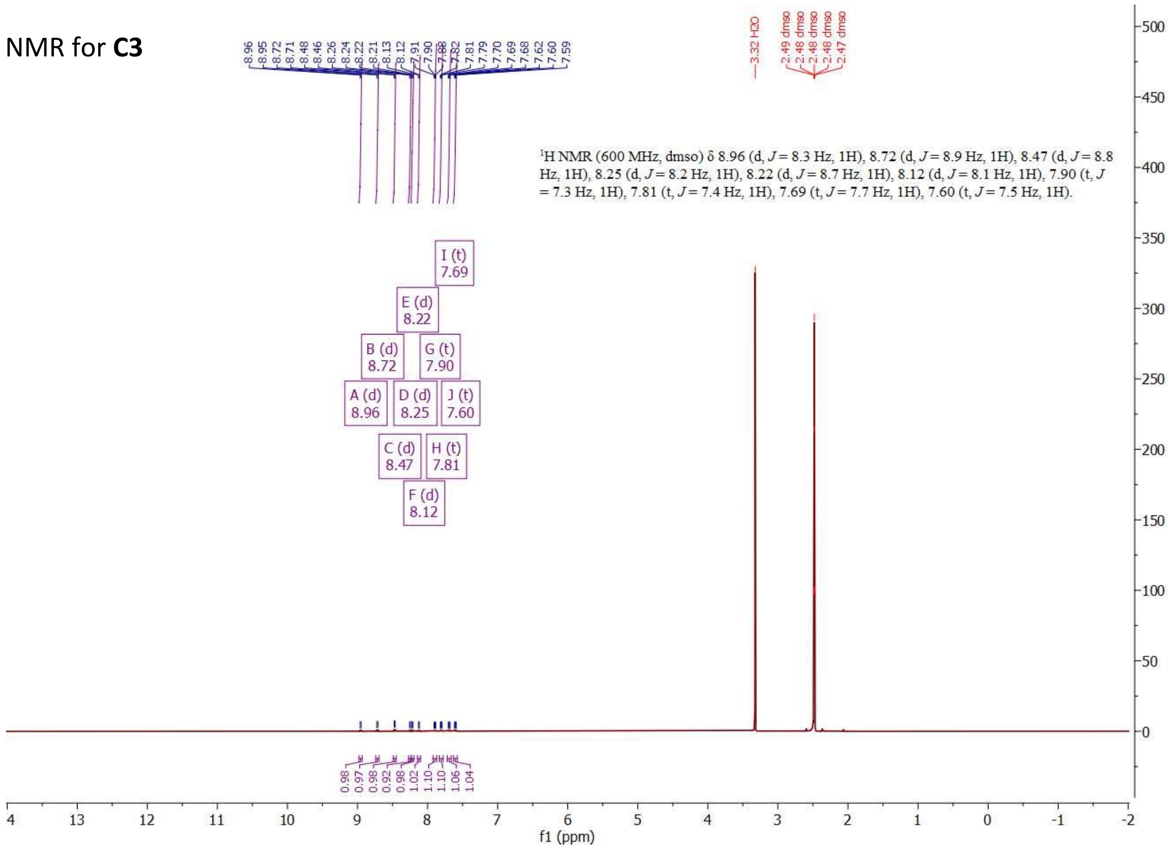

Mass spectrum for **C3**

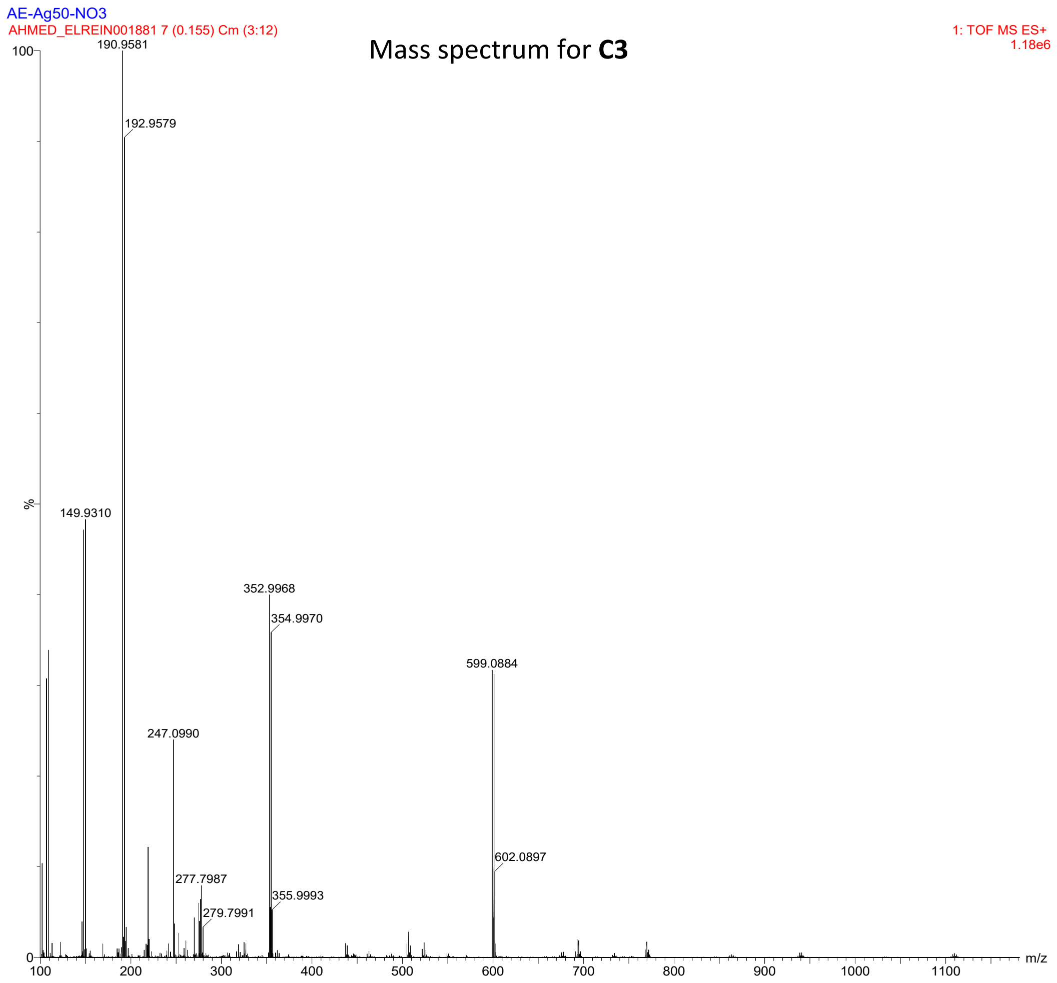

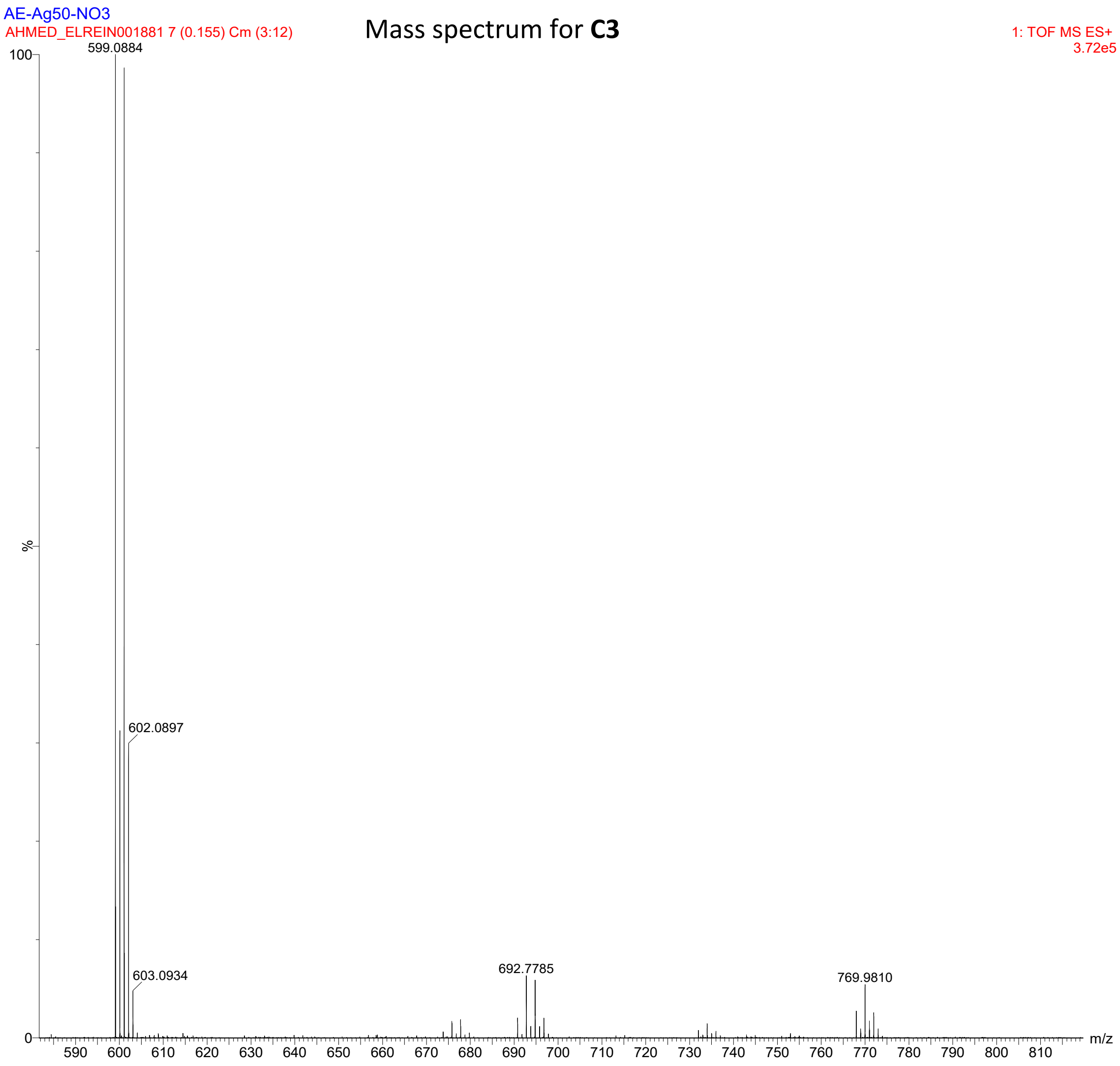

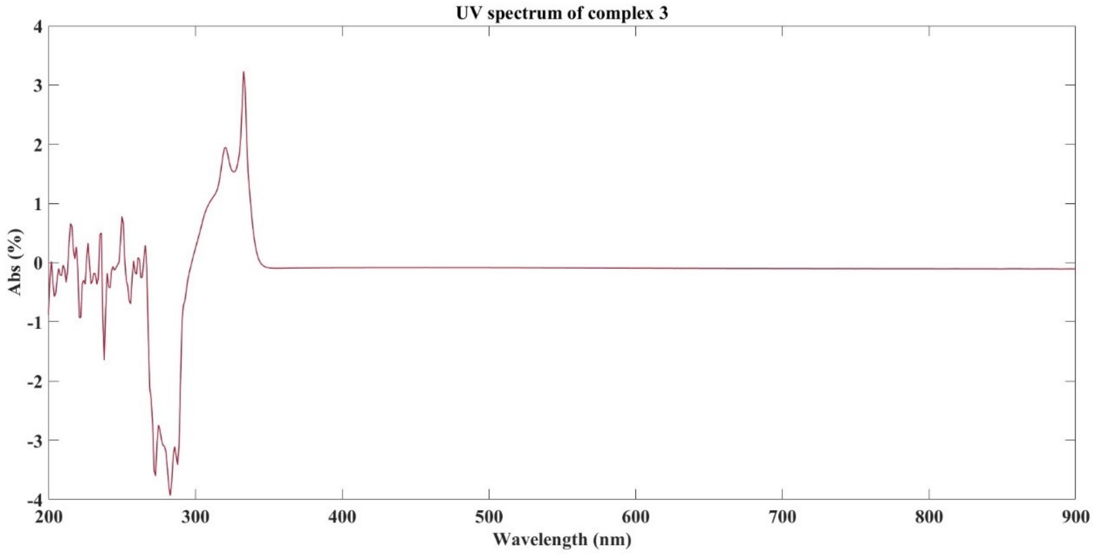

Figure S17. *Characterisation data for complex **3**.*

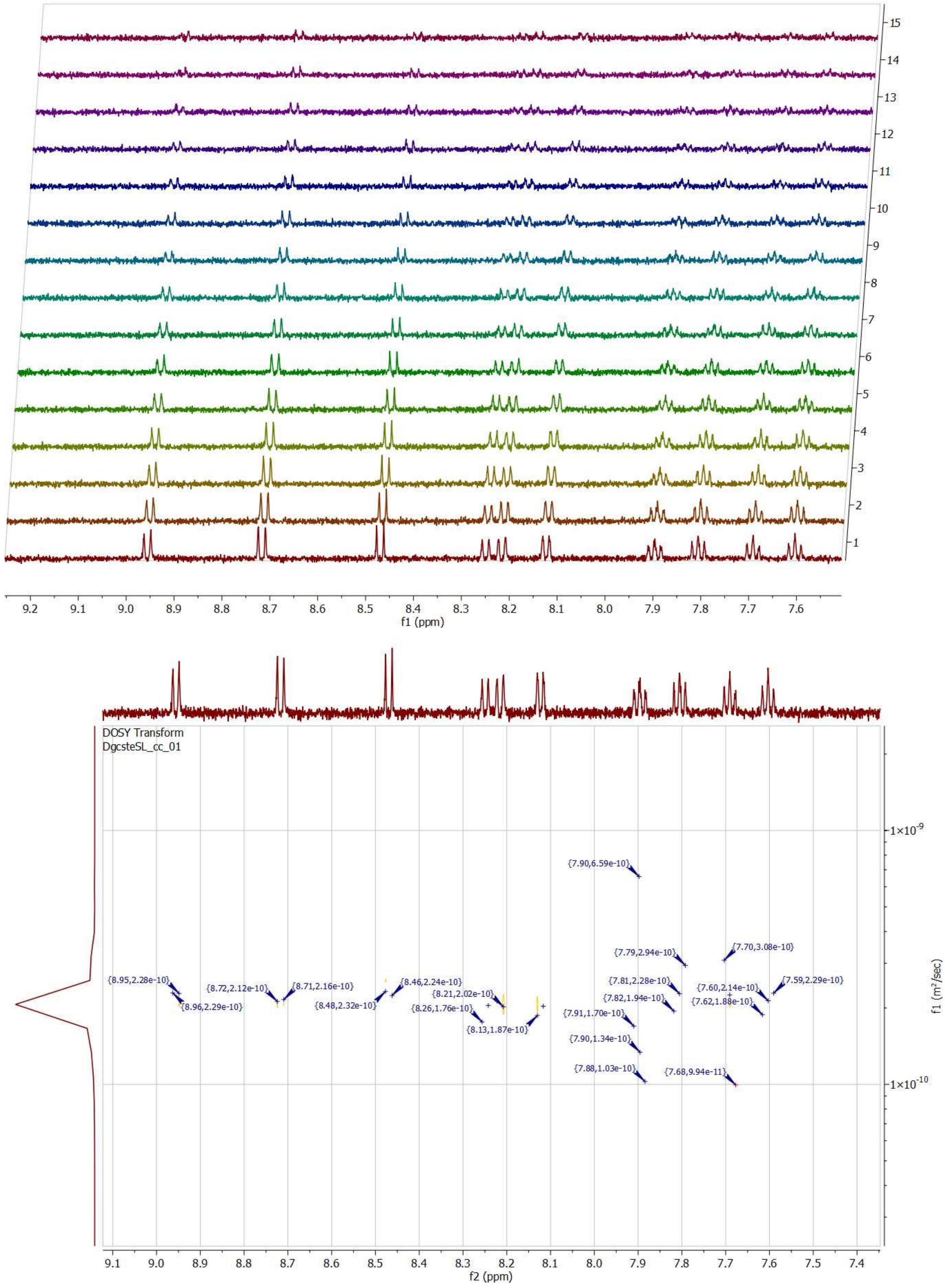

Figure S18. *Diffusion* [1]*H NNMR studies for complex **3**.*

*Compound **4***

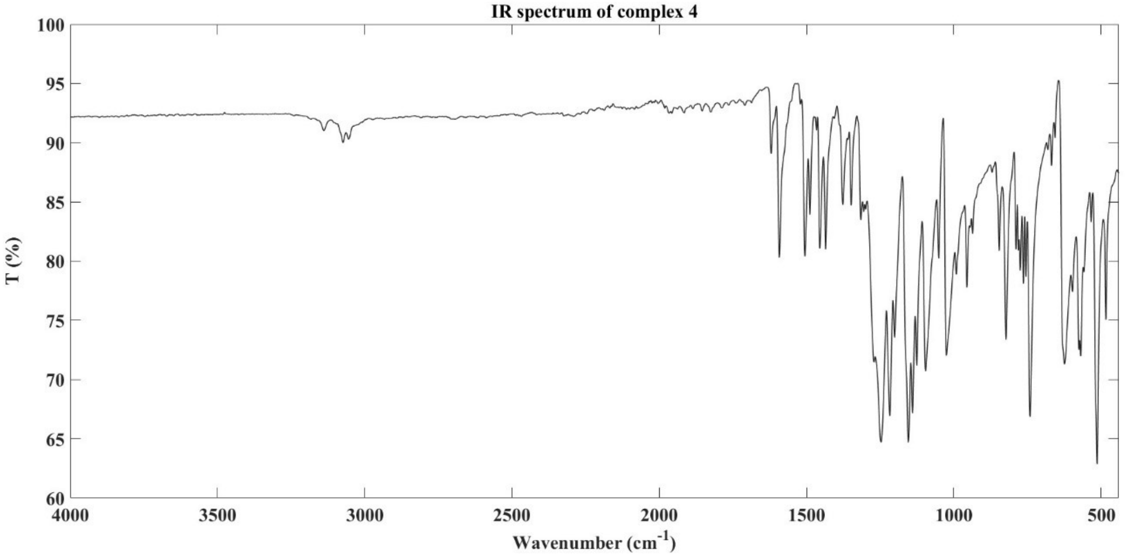

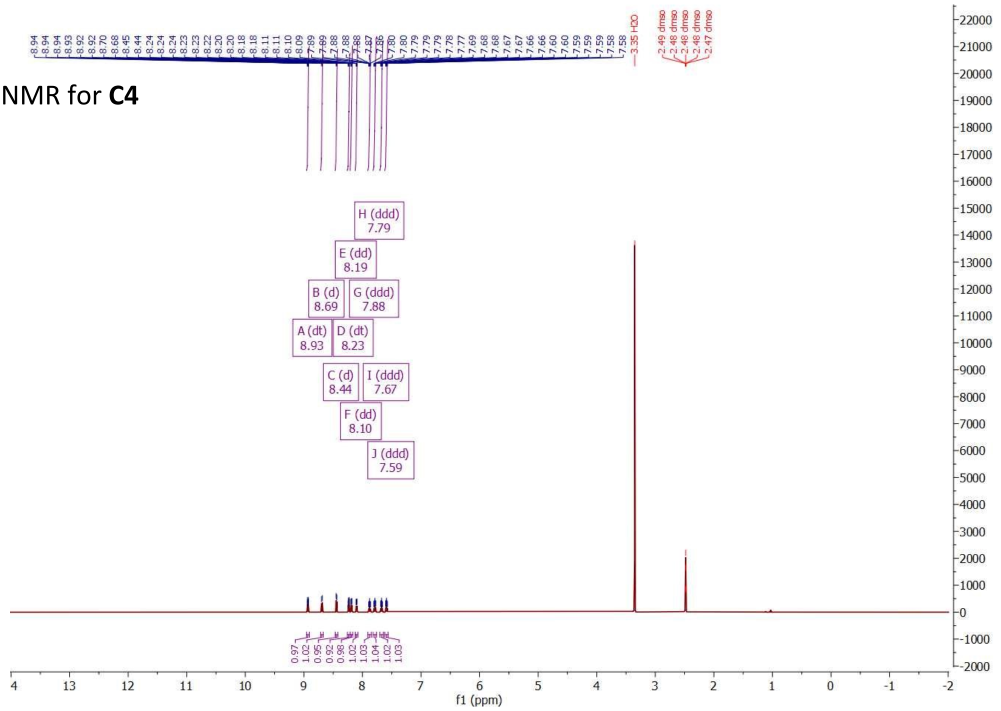

^{1}H NMR for **C4**

^{19}F NMR for **C4**

^{19}F NMR (376 MHz, dmso) δ -77.74.

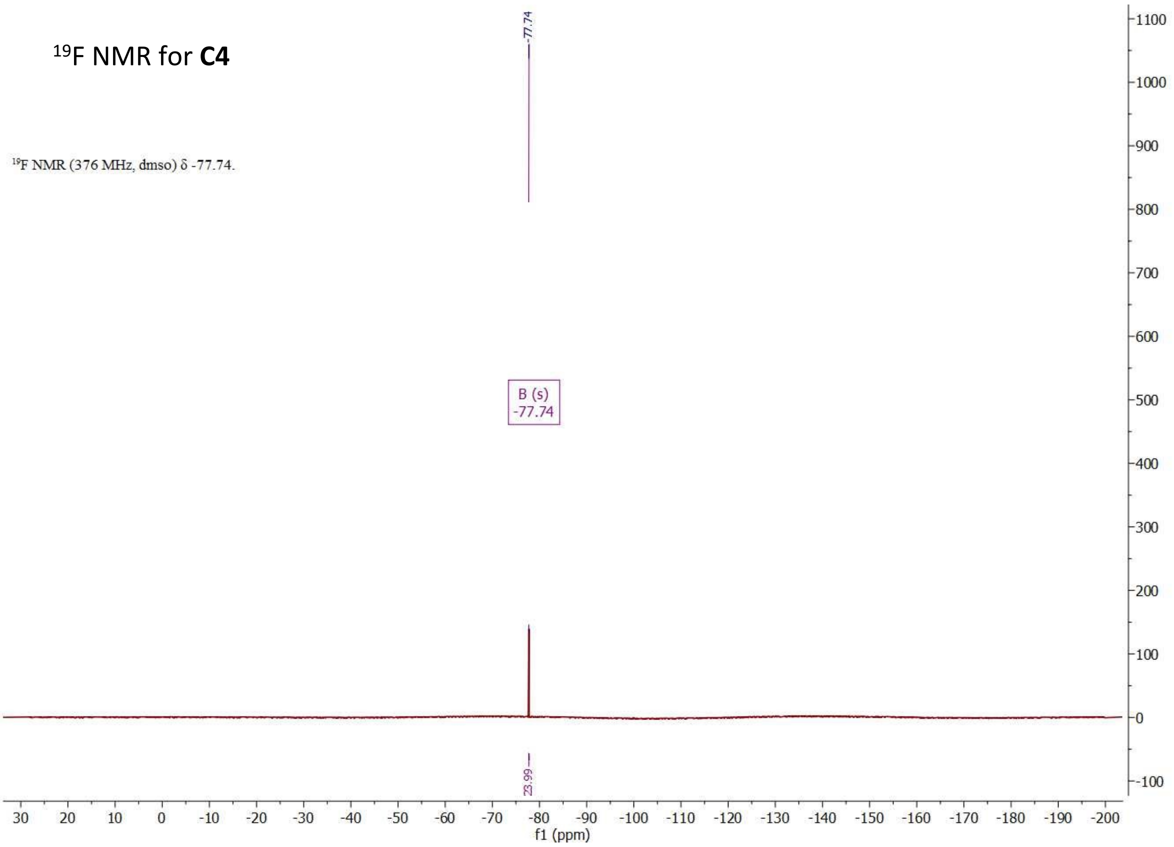

Mass spectrum for **C4**

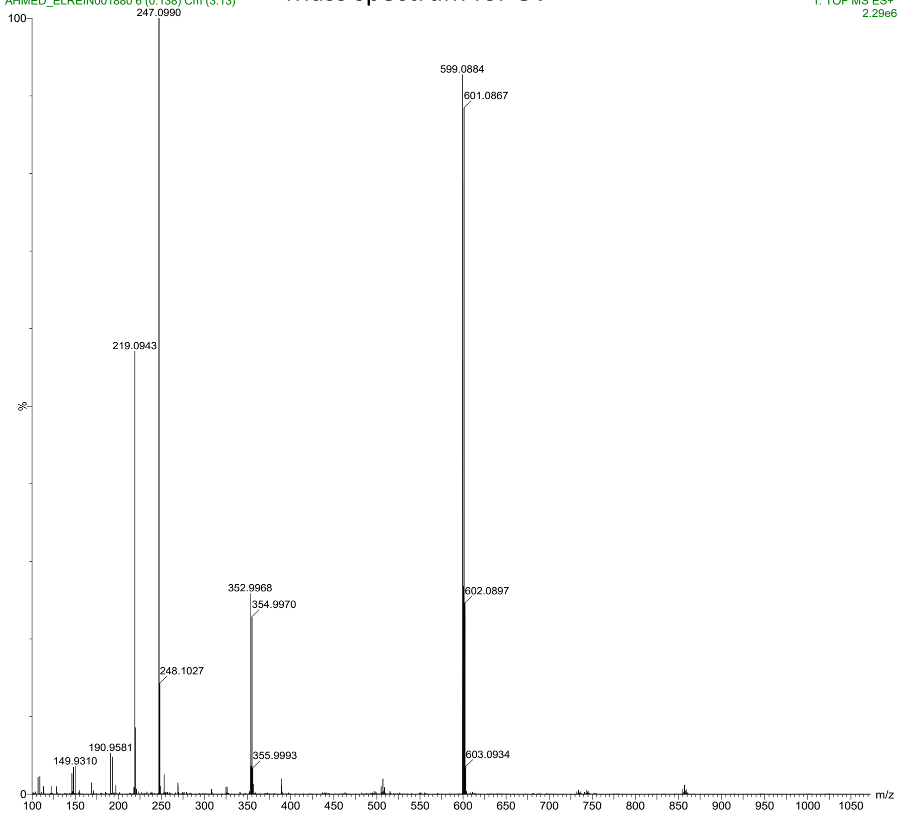

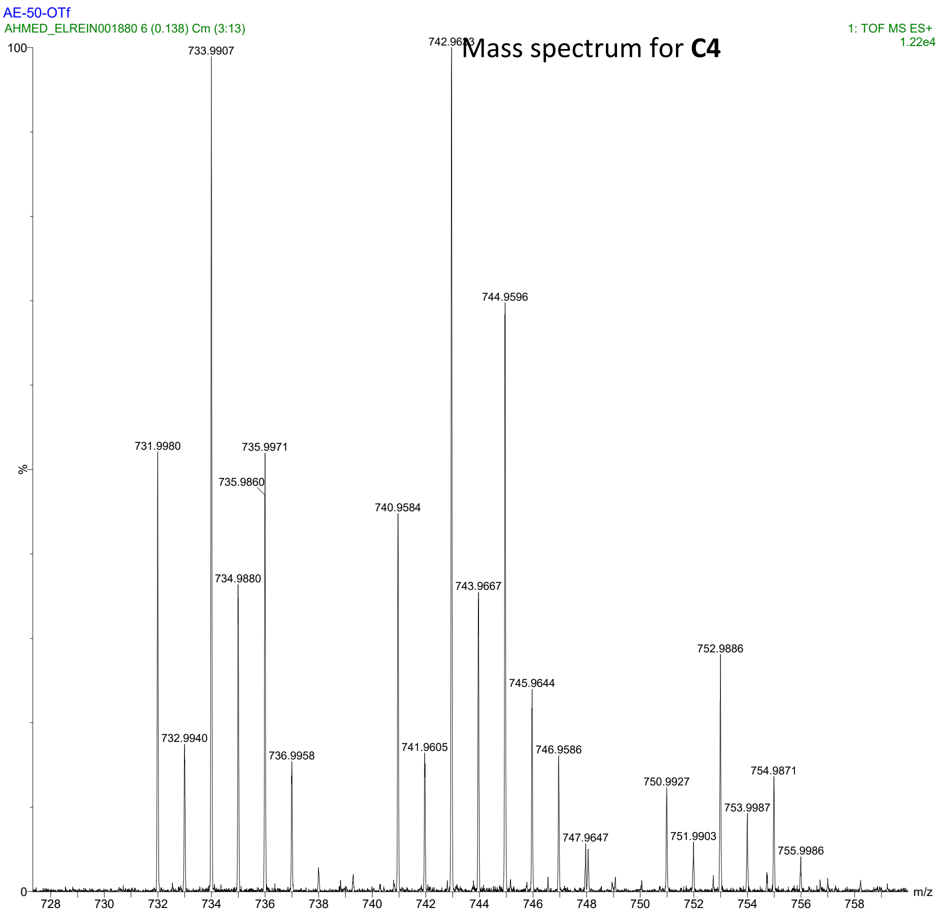

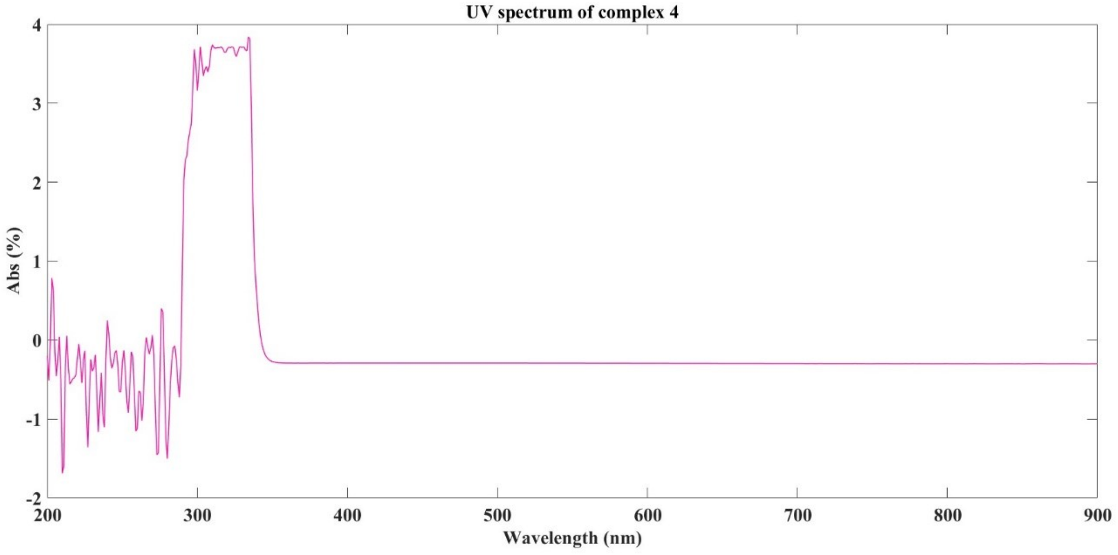

Figure S19. *Characterisation data for complex* ***4****.*

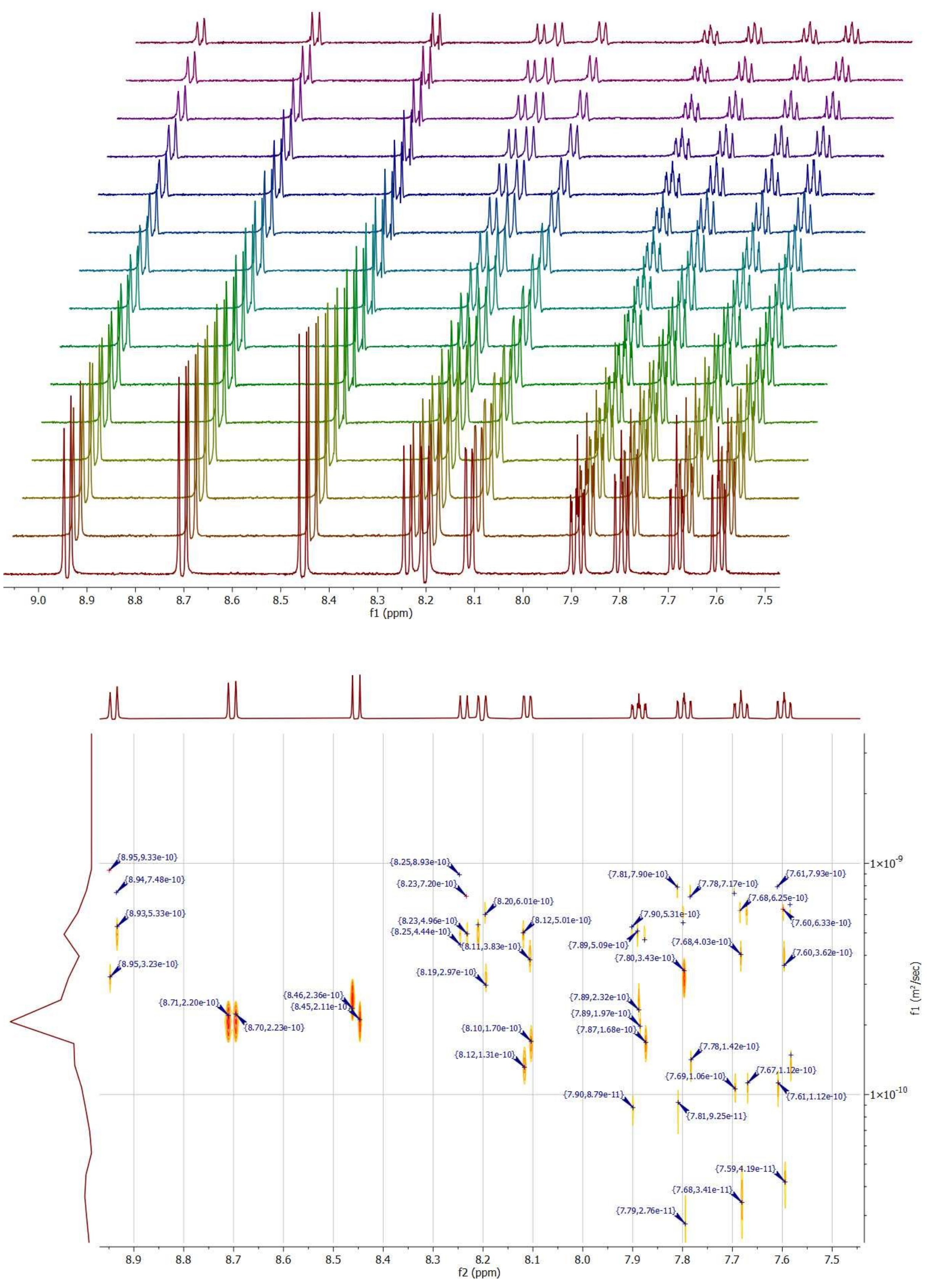

Figure S20.. Diffusion ^{1}H NNMR studies for complex **4**.

Compound 3 vs Compound 4 vs L2

- ***UV spectrum***

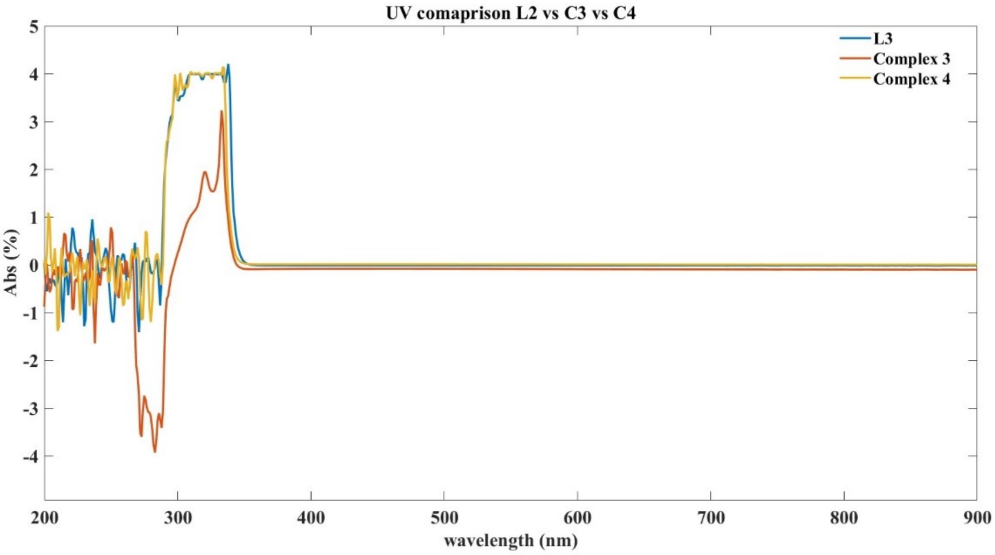

Figure S21. *UV studies comparison between Ag(**L2**) based complexes.*

- ***^{1}H NMR in d^{6}-DMSO:***

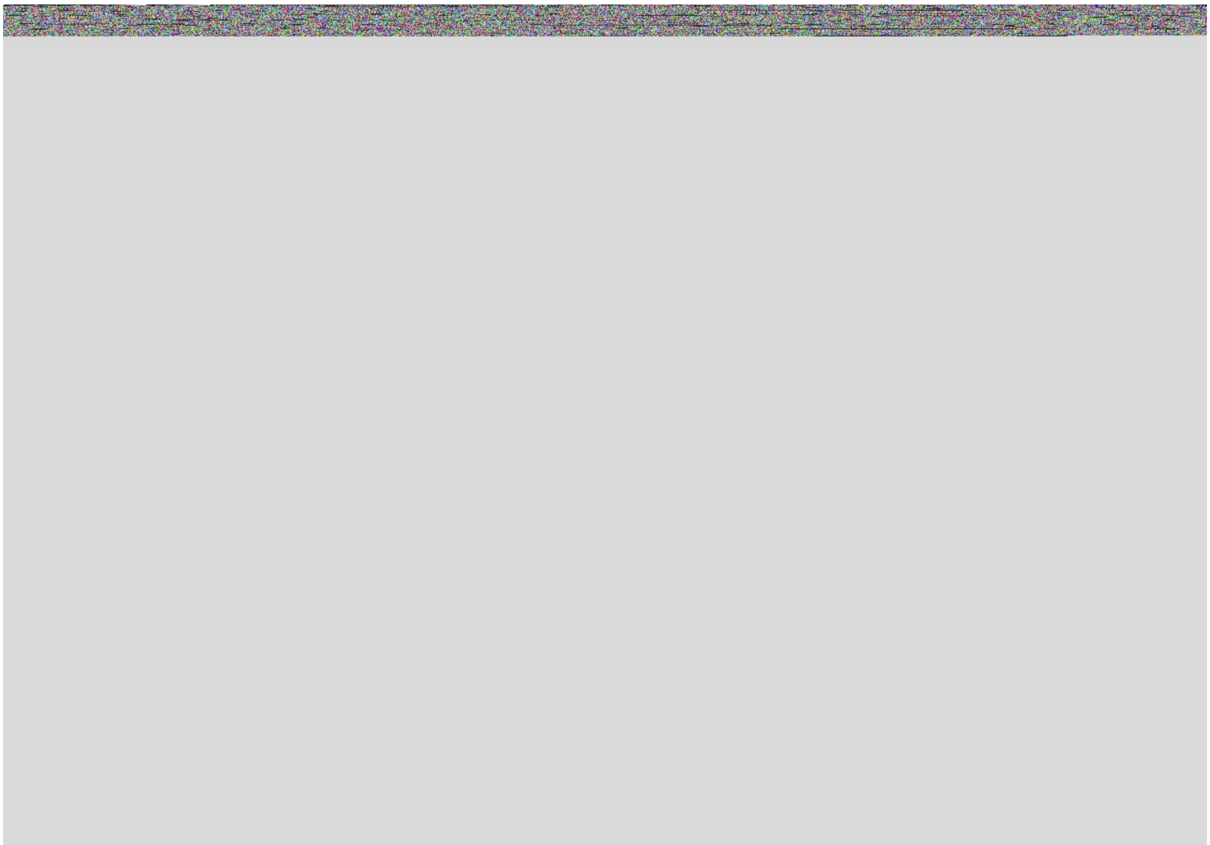

Figure S22. *^{1}H NMR comparison between Ag(**L2**) based complexes and anion effect.*

*Compound **5***

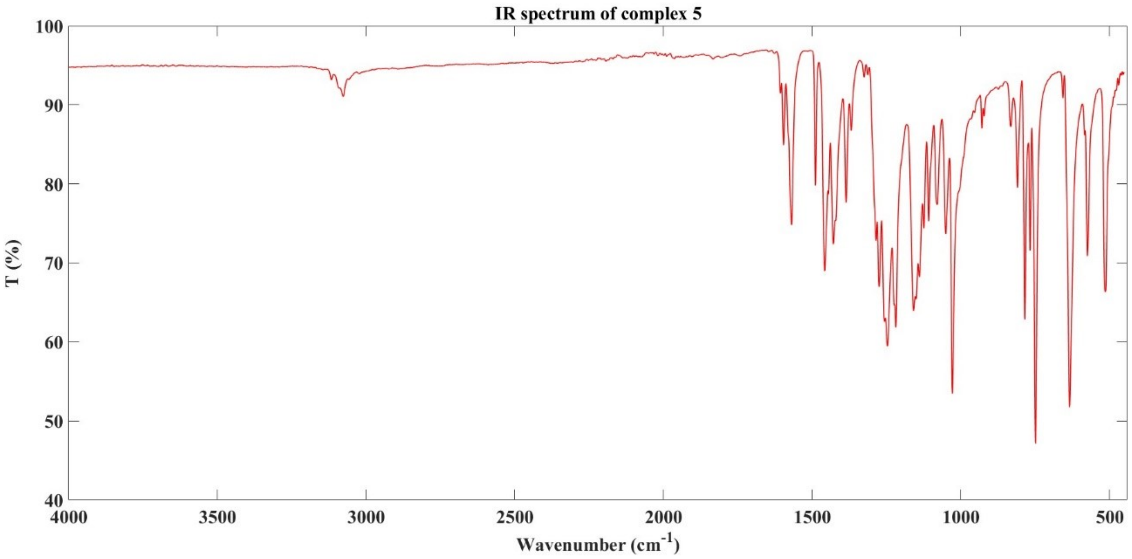

^{1}H NMR for **C5**

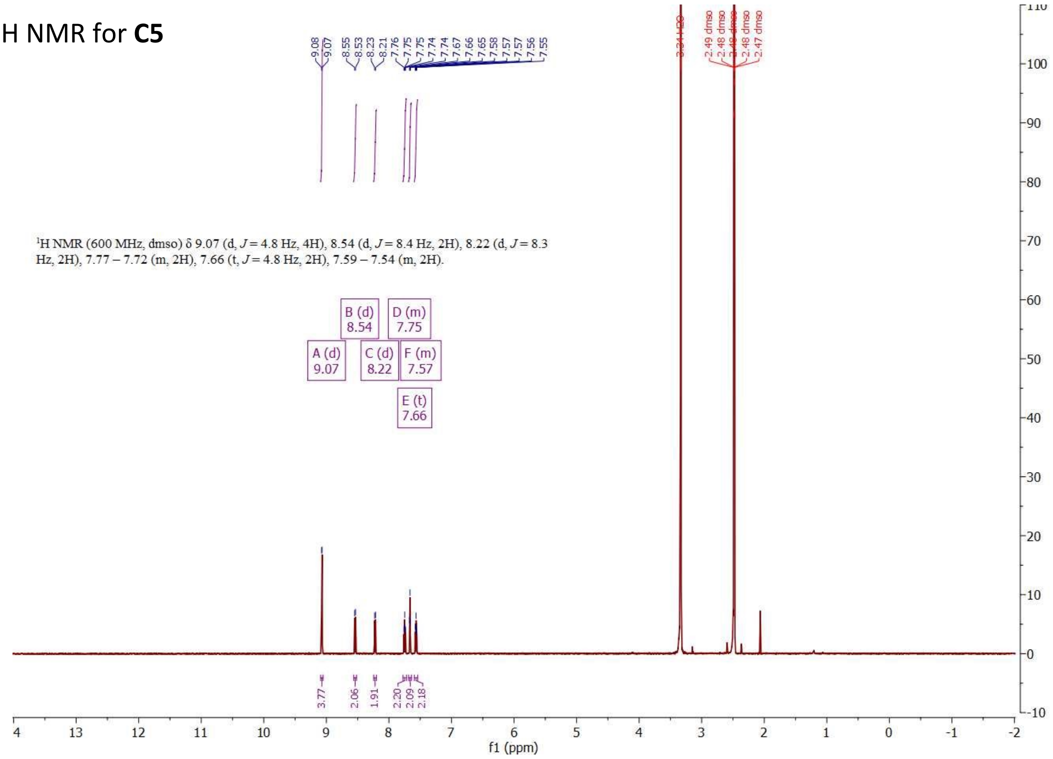

^{19}F NMR for **C5**

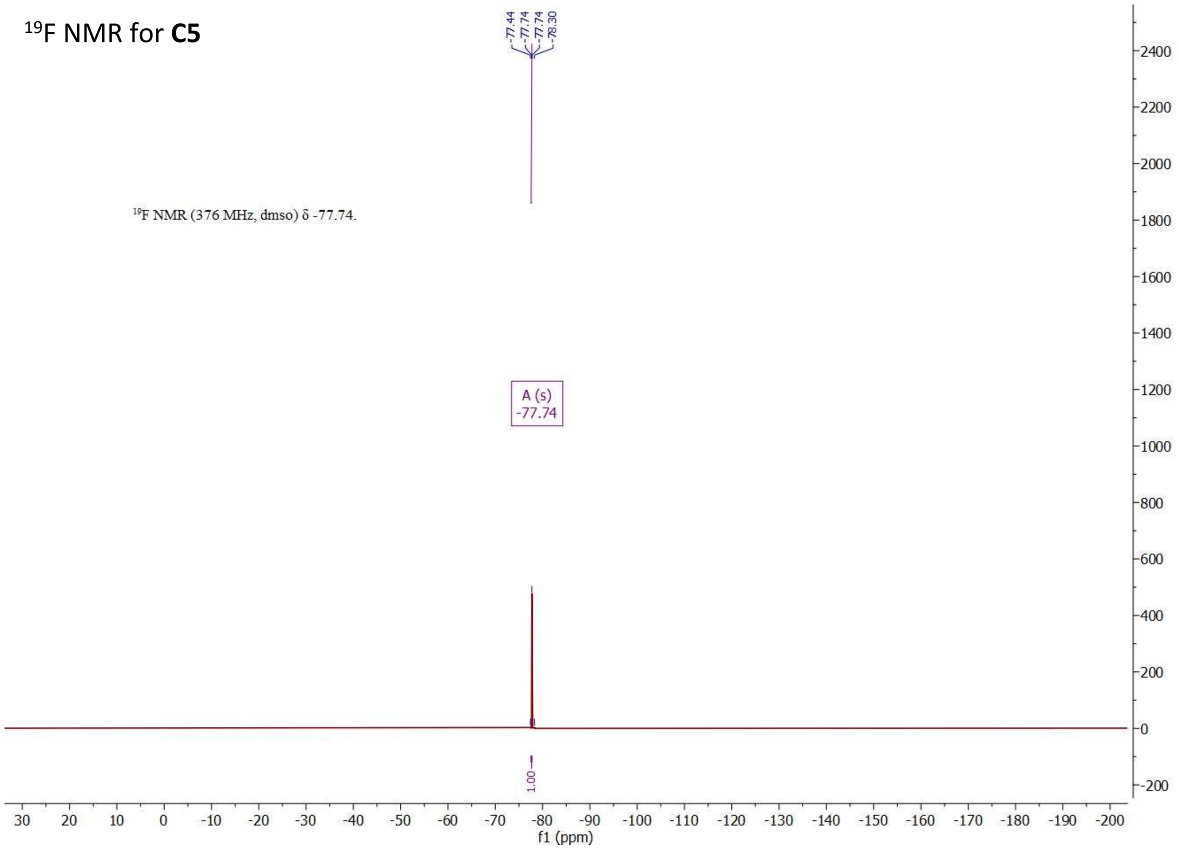

Mass spectrum for **C5**

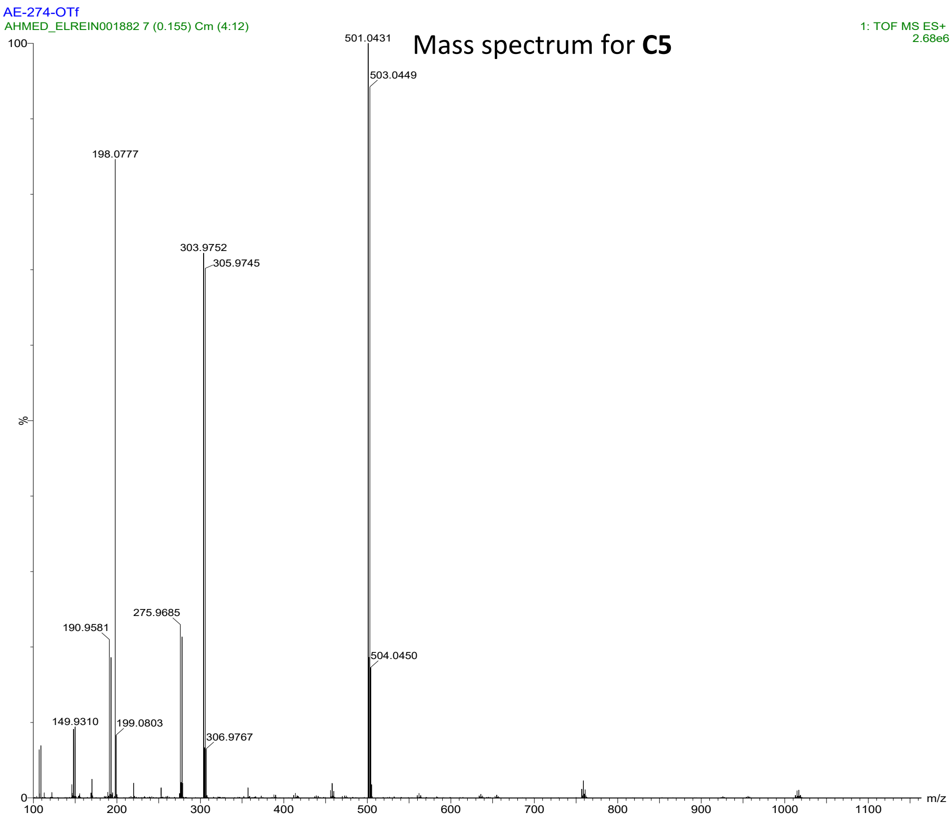

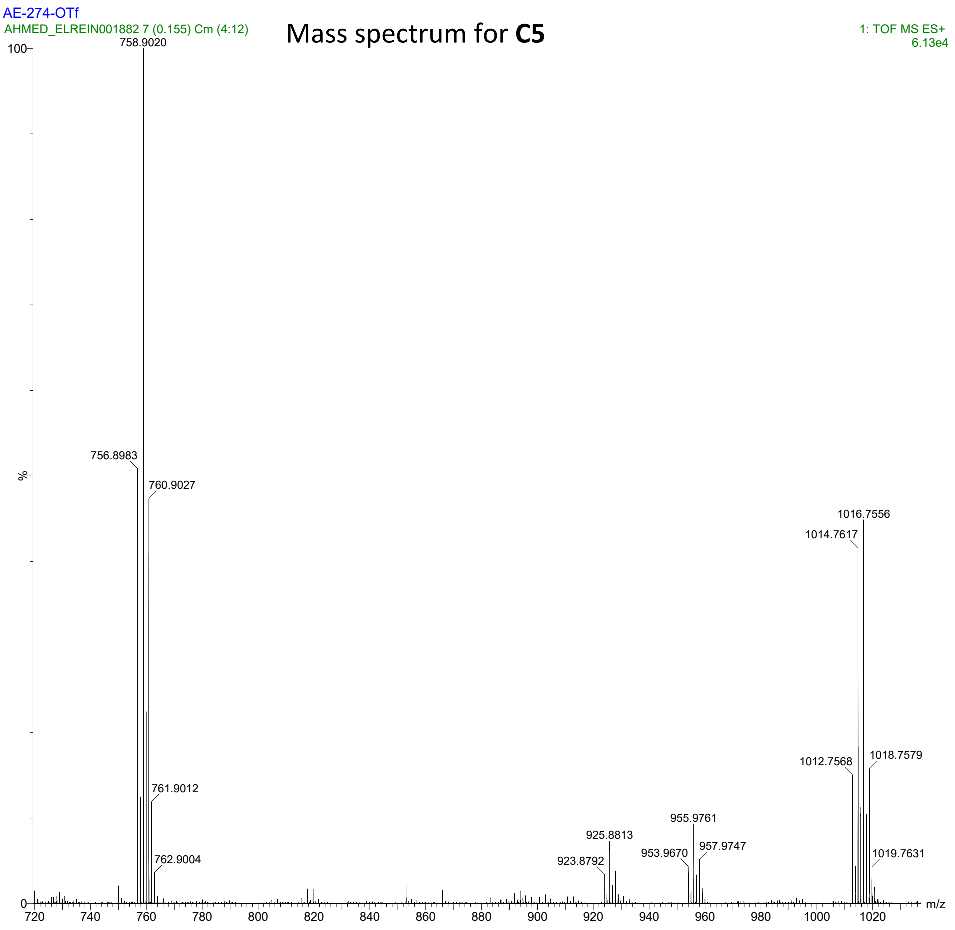

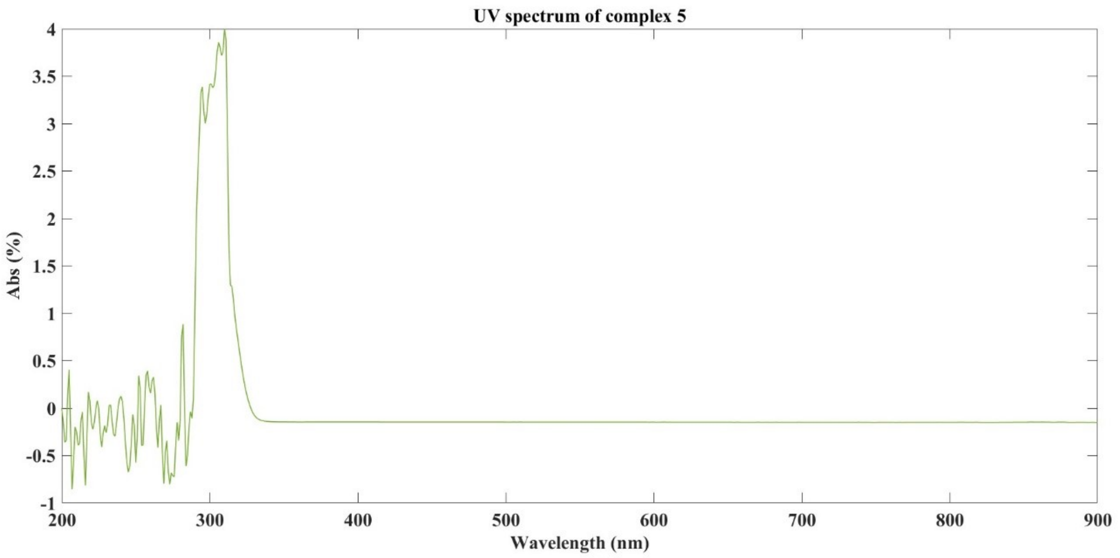

Figure S23. *Characterisation data for complex* ***5****.*

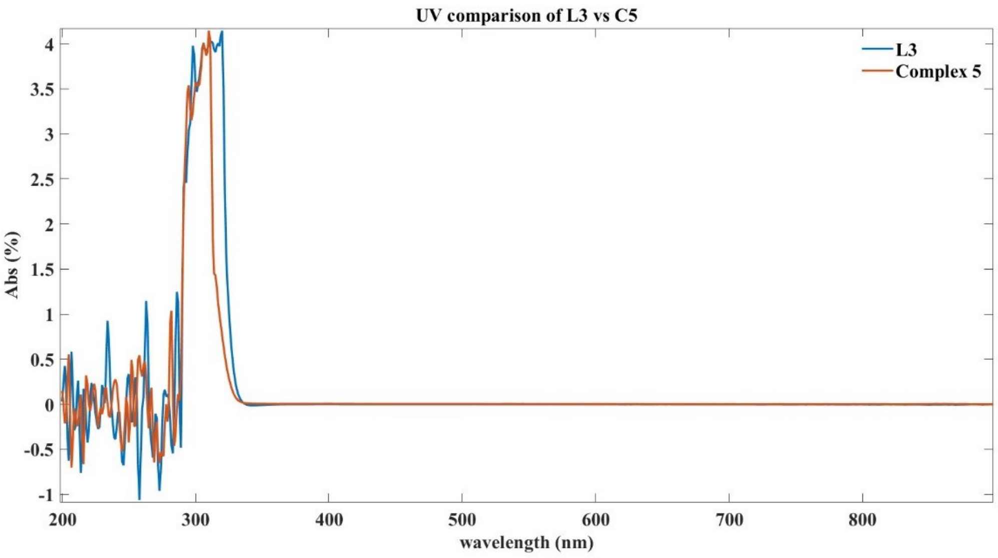

Figure S24. *UV studies comparison between **L3** vs Ag(**L3**).*

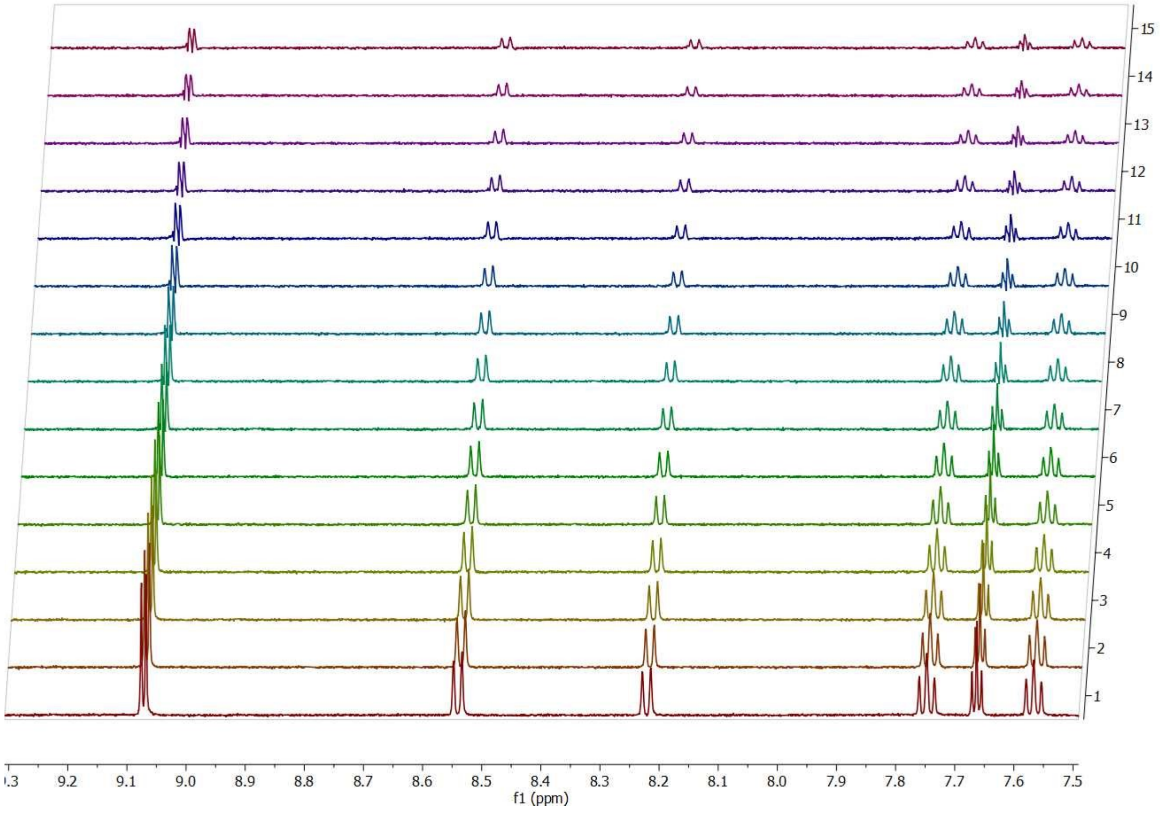

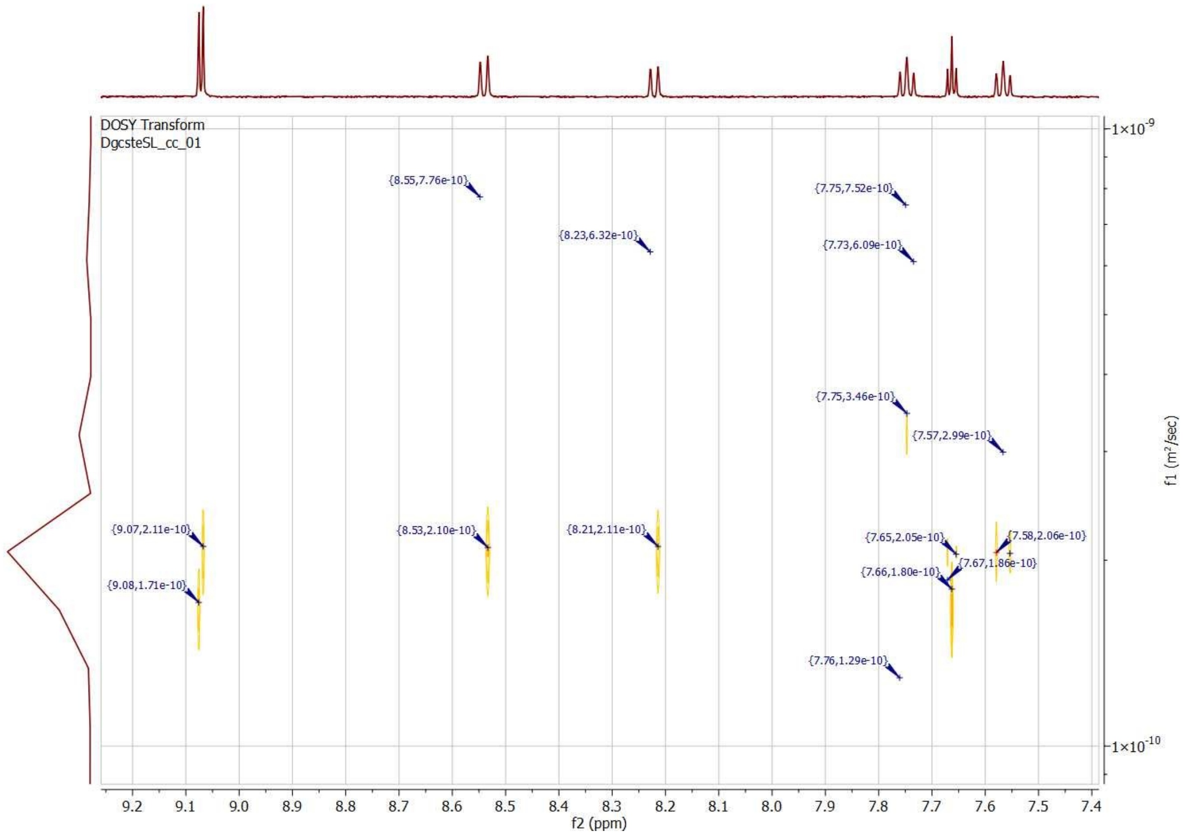

Figure S25.*.** *Diffusion* ^{1}*H NNMR studies for complex* ***5*.*

9. References

1. C. Banti, M. Kapetana, C. Papachristodoulou, C. Raptopoulou, V. Psycharis, P. Zoumpoulakis, T. Mavromoustakos and S. Hadjikakou, *Dalton Transactions*, 2021, 50, 13712-13727.
2. C. N. Banti and S. K. Hadjikakou, MDPI, 2024, vol. 13, p. 850.
3. C. N. Banti, C. Papatriantafyllopoulou, C. Papachristodoulou, A. G. Hatzidimitriou and S. K. Hadjikakou, *Journal of Medicinal Chemistry*, 2023, 66, 4131-4149.
4. I. Milionis, C. N. Banti, I. Sainis, C. P. Raptopoulou, V. Psycharis, N. Kourkoumelis, S. K. Hadjikakou, I. Milionis, C. N. Banti, I. Sainis, C. P. Raptopoulou, V. Psycharis, N. Kourkoumelis and S. K. Hadjikakou, *Journal of Biological Inorganic Chemistry*, 2018, 23, 705-723.
5. M.-E. K. Stathopoulou, C. N. Banti, N. Kourkoumelis, A. G. Hatzidimitriou, A. G. Kalampounias and S. K. Hadjikakou, *Journal of inorganic biochemistry*, 2018, 181, 41-55.
6. A. R. Katritzky and J. Wu, *Synthesis*, 1994, 1994, 597-600.
7. W. Peczynska-Czoch, F. Pognan, L. Kaczmarek and J. Boratynski, *Journal of medicinal chemistry*, 1994, 37, 3503-3510.
8. F. Tomas, J. Catalan, P. Perez and J. Elguero, *The Journal of Organic Chemistry*, 1994, 59, 2799-2802.
9. A. Habib, M. Nazari, M. A. Iqbal, H. N. Bhatti, M. K. Ahmed and A. A. Majid, *Journal of Saudi chemical society*, 2019, 23, 795-808.
10. J. Guo, S. Zhang, J. Ren, H. Li, S. Wang, Y. Hu and G. Zhou, *Molecular Catalysis*, 2023, 549, 113481.
11. C. Richardson and P. J. Steel, *Dalton Transactions*, 2003, 992-1000.
12. K. Griffiths and G. E. Kostakis, *Dalton Transactions*, 2018, 47, 12011-12034.
13. M. Kallitsakis, E. Loukopoulos, A. Abdul-Sada, G. J. Tizzard, S. J. Coles, G. E. Kostakis and I. N. Lykakis, *Advanced Synthesis & Catalysis*, 2017, 359, 138-145.
14. E. Loukopoulos, A. Abdul-Sada, G. Csire, C. Kállay, A. Brookfield, G. J. Tizzard, S. J. Coles, I. N. Lykakis and G. E. Kostakis, *Dalton Transactions*, 2018, 47, 10491-10508.
15. E. Loukopoulos, A. Abdul-Sada, E. M. Viseux, I. N. Lykakis and G. E. Kostakis, *Crystal Growth & Design*, 2018, 18, 5638-5651.
16. E. Loukopoulos, N. F. Chilton, A. Abdul-Sada and G. E. Kostakis, *Crystal Growth & Design*, 2017, 17, 2718-2729.
17. S. I. Sampani, V. Zdorichenko, J. Devonport, G. Rossini, M. C. Leech, K. Lam, B. Cox, A. Abdul-Sada, A. Vargas and G. E. Kostakis, *Chemistry–A European Journal*, 2021, 27, 4394-4400.
18. C.-X. An, X.-L. Han, P.-B. Wang, Z.-H. Zhang, H.-K. Zhang and Z.-J. Fan, *Transition Metal Chemistry*, 2008, 33, 835-841.
19. J. Hu, Y. Zhao, F. Yang, C. Liao and J. a. Zhao, *Journal of Coordination Chemistry*, 2018, 71, 1368-1379.
20. L. Wan, Y. Chen, Y. Zhang, X. Peng, S. Chen and L. Jia, *CrystEngComm*, 2022, 24, 6662-6668.
21. S. Pandey and T. Mandal, *European Journal of Inorganic Chemistry*, 2021, 2021, 1763-1769.
22. S. Pandey, T. Mandal and S. K. Mandal, *Polyhedron*, 2023, 231, 116249.
23. P. Vera-Luque, R. Alajarín, J. Alvarez-Builla and J. J. Vaquero, *Organic Letters*, 2006, 8, 415-418.
